# A Computational Bayesian Framework for Entropy-Based Inference in the Inverse Gaussian Distribution Under Progressive Type-II Censoring

**DOI:** 10.3390/e28080871

**Published:** 2026-08-02

**Authors:** Mohamed A. T. El-Shahat, Reman Abo Hashem, Doaa Basalamah, Tmader Alballa, Wael S. Abu El Azm

**Affiliations:** 1 Department of Statistics and Insurance, Faculty of Commerce, Zagazig University, Zagazig 44519, Egypt; maelshahat@commerce.zu.edu.eg (M.A.T.E.-S.); remanabohashem@gmail.com (R.A.H.); drwaelshehta1982@gmail.com (W.S.A.E.A.); 2 Mathematics Department, Faculty of Science, Umm Al-Qura University, P.O. Box 24231, Makka 21955, Saudi Arabia; dabasalamah@uqu.edu.sa; 3 Department of Mathematical Sciences, College of Science, Princess Nourah bint Abdulrahman University, P.O. Box 84428, Riyadh 11671, Saudi Arabia

**Keywords:** Shannon entropy, Rényi entropy, inverse Gaussian distribution, progressive Type-II censoring, maximum likelihood, Bayes estimation, Lindley’s approximation, importance sampling, Monte Carlo Markov Chain

## Abstract

Estimating entropy measures under progressive censoring poses a significant challenge in reliability and lifetime analysis. Despite the widespread use of the inverse Gaussian distribution in modeling skewed lifetime data, a comprehensive inferential framework for its Shannon and Rényi entropies under progressive Type-II censoring remains absent. This paper develops a unified Bayesian framework integrating maximum likelihood and Bayesian inference under squared error, general entropy, and LINEX loss functions, employing Lindley’s approximation, importance sampling, and Markov chain Monte Carlo methods. Two parameter configurations were examined to assess robustness under varying likelihood surface complexity, along with prior sensitivity analysis. Results demonstrate that Markov chain Monte Carlo and importance sampling are the only consistently reliable methods across all scenarios, whereas maximum likelihood suffered severe bias and collapse of Wald interval coverage under challenging settings, and Lindley’s approximation exhibited numerical instability at small samples. Shannon entropy proved substantially more sensitive to parameter variation than Rényi entropy, with the LINEX and general entropy loss functions showing superior performance. The study offers clear practical guidance, validated on real lifetime data.

## 1. Introduction

Entropy is a fundamental measure of uncertainty in probability distributions, reflecting the average information content of a sample. Shannon entropy, introduced by Shannon [1], is the most widely used formulation of this concept, later generalized by Rényi through an additional parameter α that provides flexibility in tuning the measure’s sensitivity. Entropy is particularly valuable in reliability and lifetime data analysis, outperforming traditional measures such as the mean and variance in characterizing skewed and heavy-tailed distributions. Shannon entropy is defined as:    

(1) $$H=-E\left[\ln f(x)\right]=-\int_{0}^{\infty }\left[\ln f(x)\right]f\left(x\right),dx$$

Rényi [2] extended this measure through an order parameter α, defined as:

(2) $$H_{\alpha }=\frac{1}{1-\alpha }\ln\left[\int_{0}^{\infty }f{\left(x\right)}^{\alpha },dx\right]\;,\;\alpha \left(\neq 1\right)>0$$

Entropy has been applied in diverse fields, including password security [3], agricultural economics [4], seismology [5], and software reliability [6]. Shannon entropy serves disciplines ranging from ecology to economics, while Rényi entropy has become essential in reliability engineering, life testing, and information theory.

Life-tests typically involve constraints on overall testing duration, necessitating censoring schemes to reduce time and cost. In such schemes, the experiment terminates before all units fail. This study employs progressive Type-II censoring, where the stopping rule depends on a pre-specified number of observed failures. In this scheme, *n* units are placed on test at time 0, with *m* failures to be observed. At the first failure $X_{\left(1\right)}$, $R_{1}$ surviving units are randomly withdrawn; at the second failure $X_{\left(2\right)}$, $R_{2}$ units are withdrawn, and so on. At the $m^{th}$ failure, all remaining units $R_{m}=n-\sum_{i=1}^{m-1}R_{i}-m$ are removed. The observed data consist of the pairs $\left(X_{\left(i\right)},R_{i}\right)$ with pre-specified removal values. Although more complex to design and analyze than traditional censoring, this scheme offers greater cost and experimental efficiency while maintaining the desired estimation accuracy.

**Figure 1:**
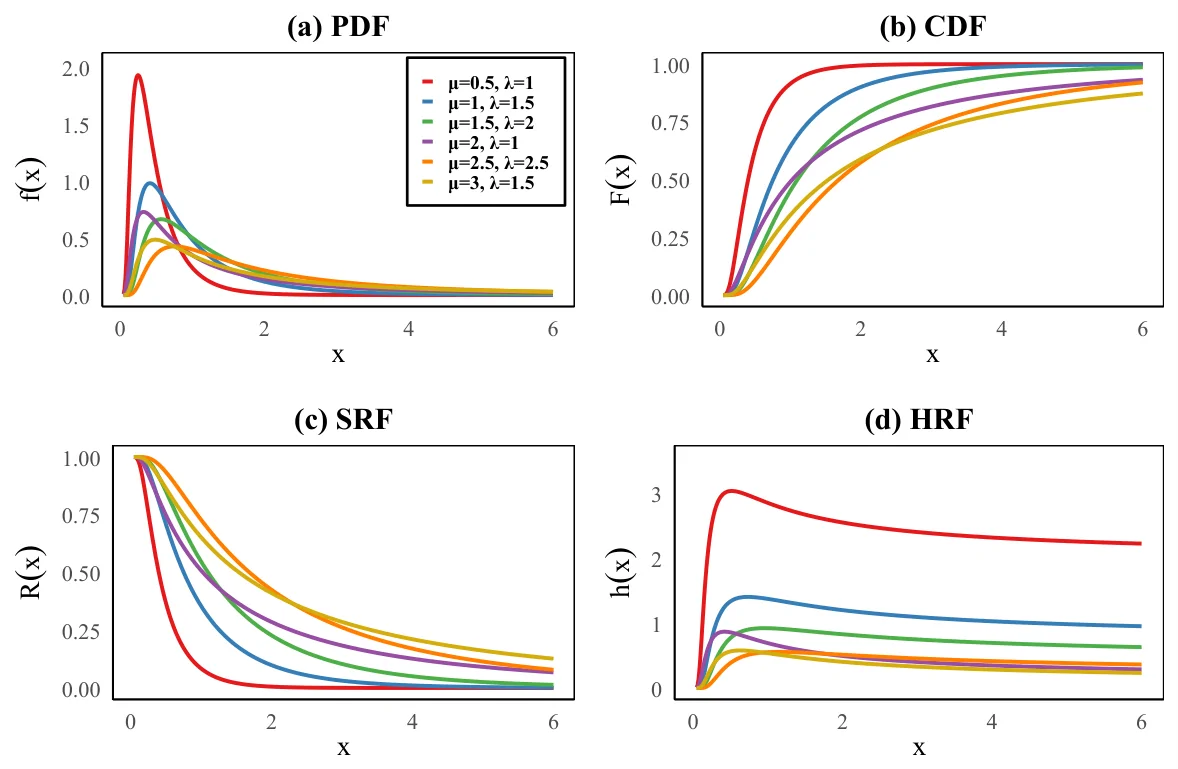
Inverse Gaussian: (**a**) probability density function (PDF), (**b**) cumulative density function (CDF), (**c**) survival function (SRF), (**d**) hazard rate function (HRF).

The inverse Gaussian distribution (IG) has attracted considerable attention due to its usefulness in statistical analysis, see Figure 1. The origin of the IG dates back to Tweedie [7] and Wald [8] through studies on Brownian motion. Later, Tweedie [9,10,11] provided a more detailed development of this distribution; with scale parameter λ and location parameter (mean) μ, the following probability density function (PDF), cumulative density function (CDF), survival function R(x) and hazard rate function r(x) are defined, respectively, as follows:

(3) $$\begin{array}{cc} f(x;\mu ,\lambda ) & =\sqrt{\frac{\lambda }{2\pi x^{3}}}e^{-\frac{\lambda {\left(x-\mu \right)}^{2}}{2\mu^{2}x}}\;\;,\mu ,\lambda >0 \end{array}$$

(4) $$\begin{array}{cc} F(x;\mu ,\lambda ) & =\Phi \left[\sqrt{\frac{\lambda }{x}}\left(\frac{x}{\mu }-1\right)\right]+e^{\frac{2\lambda }{\mu }}\Phi \left[-\sqrt{\frac{\lambda }{x}}\left(\frac{x}{\mu }+1\right)\right] \end{array}$$

(5) $$\begin{array}{cc} R(x) & =1-\left(\Phi \left[\sqrt{\frac{\lambda }{x}}\left(\frac{x}{\mu }-1\right)\right]+e^{\frac{2\lambda }{\mu }}\Phi \left[-\sqrt{\frac{\lambda }{x}}\left(\frac{x}{\mu }+1\right)\right]\right) \end{array}$$

(6) $$\begin{array}{cc} r(x) & =\frac{\sqrt{\frac{\lambda }{2\pi x^{3}}}e^{-\frac{\lambda {\left(x-\mu \right)}^{2}}{2\mu^{2}x}}}{1-\left(\Phi \left[\sqrt{\frac{\lambda }{x}}\left(\frac{x}{\mu }-1\right)\right]+e^{\frac{2\lambda }{\mu }}\Phi \left[-\sqrt{\frac{\lambda }{x}}\left(\frac{x}{\mu }+1\right)\right]\right)} \end{array}$$

where Φ denotes the CDF of a standard normal distribution and can be expressed using the error function $\Phi \left(x\right)=\frac{1}{2}\left[1+erf\left(\frac{x}{\sqrt{2}}\right)\right]$. The HRF of IG decreases in *x* for λ,μ>0.

Due to the extensive use of the IG distribution, numerous authors have examined the estimation of its parameters and related properties under various censoring schemes. Nath [12] introduced generalized censored samples and obtained maximum likelihood estimates. Whitmore [13] employed the EM algorithm for progressively right-censored data. Anaya and O’Reilly [14] assessed goodness-of-fit under censoring. Ismail and Auda [15] proposed kernel estimation under Type-II censoring. Jia et al. [16] developed progressive hybrid Type-I censoring and derived MLE and Bayesian estimators. Rostamian and Nematollahi [17] assessed stress-strength reliability under progressive Type-II censoring. Jayalath and Chhikara [18] performed Bayesian and fiducial survival analysis using Gibbs sampling for various censoring types. Roy et al. [19] considered progressive Type-I interval censoring. Bera and Jana [20] examined stress-strength reliability with bootstrap and Bayes estimators. Xavier et al. [21] formulated goodness-of-fit tests based on a fixed-point characterization for complete and right-censored data.

This section reviews entropy estimation under various censoring schemes. Kang et al. [22] and Cho et al. [23,24] estimated Shannon entropy for double exponential, Rayleigh, and Weibull distributions under different censoring types. Anis and De [25] studied Shannon and Rényi entropies for the Unit-Gompertz distribution. Hashem and Alyami [26] analyzed entropies for the exponential doubly Poisson distribution. Okasha and Nassar [27] and Shrahili et al. [28] estimated entropy for inverse Weibull and log-logistic distributions under progressive Type-II censoring. Maiti et al. [29] compared MLE and Bayesian estimates for generalized exponential distributions. Patra et al. [30] proposed improved entropy estimators for exponential distributions. Ahmed et al. [31] studied gamma distributions under progressive Type-I censoring, while Gong et al. [32] considered generalized inverse exponential distributions under Stepwise Type-II truncated samples.

Despite the numerous studies addressing entropy estimation for lifetime distributions under various censoring schemes, these efforts have primarily focused on distributions such as the Weibull, exponential, generalized exponential, and log-logistic models. To the best of our knowledge, no previous study has investigated the estimation of Shannon and Rényi entropies for the IG distribution under progressive Type-II censoring, despite the importance of this distribution in reliability analysis and degradation modeling, and the flexibility of this censoring scheme in life-testing experiments. This gap is particularly consequential given that the IG distribution poses well-documented estimation challenges. Folks and Chhikara [33] reported instability of maximum likelihood estimators for its parameters, particularly under small sample sizes or extreme parameter values, while Giner and Smyth [34] highlighted numerical difficulties in handling the IG likelihood within the statmod package in R, including issues related to flatness and numerical optimization. How these challenges may propagate or become amplified when estimating functions of parameters such as entropy measures has remained unexplored, and constitutes the starting point of the present study. To address this gap, this paper presents a comprehensive computational Bayesian framework that combines maximum likelihood estimation with Bayesian inference under multiple loss functions, employing Lindley’s approximation, importance sampling, and Markov chain Monte Carlo techniques. The primary contributions of this work can be summarized as follows:

- Deriving closed-form Shannon and Rényi entropy expressions for the IG distribution, and developing a unified Bayesian computational framework under progressive Type-II censoring.
- Documenting the breakdown of maximum likelihood estimation for Shannon entropy under certain parameter configurations, and providing practical, validated guidance for reliable entropy inference using Monte Carlo Markov Chain and importance sampling.

In this paper, we address the estimation of Shannon and Rényi entropies for the IG distribution under progressive Type-II censoring. We begin by deriving the Shannon entropy and the Rényi entropy of the IG distribution by substituting in Equation (1) and Equation (2), respectively, using Equation (3) as the basis for substitution (detailed in Supplementary Materials Section S1):

(7) $$\begin{array}{cc} H & =-\int_{0}^{\infty }f\left(x\right)\left[\ln\left(\sqrt{\frac{\lambda }{2\pi x^{3}}}\;e^{-\frac{\lambda {\left(x-\mu \right)}^{2}}{2\mu^{2}x}}\right)\right]\;dx\\ \; & =-\frac{1}{2}\ln\left(\frac{\lambda }{2\pi }\right)+\frac{3}{2}\;E\left[\ln(x)\right]+\frac{\lambda }{2\mu^{2}}\;E\left[\frac{{\left(x-\mu \right)}^{2}}{x}\right]\\ \; & \;\;\;\;\;\;\vdots \\ \; & =\frac{1}{2}\ln\frac{2\pi e}{\lambda }+\frac{3}{2}\ln\mu -\frac{3}{2}\;e^{\frac{2\lambda }{\mu }}E_{1}\left(\frac{2\lambda }{\mu }\right) \end{array}$$

and

(8) $$\begin{array}{cc} H_{\alpha } & =\frac{1}{1-\alpha }\ln\left(\int_{0}^{\infty }{\left[\sqrt{\frac{\lambda }{2\pi x^{3}}}\;e^{-\frac{\lambda {\left(x-\mu \right)}^{2}}{2\mu^{2}x}}\right]}^{\alpha }\;dx\right)\\ \; & =\frac{1}{1-\alpha }\left[\ln\int_{0}^{\infty }{\left(\frac{\lambda }{2\pi }\right)}^{\frac{\alpha }{2}}x^{-\frac{3\alpha }{2}}e^{-\frac{\alpha \lambda {\left(x-\mu \right)}^{2}}{2\mu^{2}x}}\;dx\right]\\ \; & \;\;\;\;\;\;\;\vdots \\ \; & =\frac{1}{1-\alpha }\left[\frac{\alpha }{2}\;\ln\frac{\lambda }{2\pi }+\frac{\alpha \lambda }{\mu }+\ln2+\left(1-\frac{3\alpha }{2}\right)\ln\mu +\ln K_{1-\frac{3\alpha }{2}}\left(\frac{\alpha \lambda }{\mu }\right)\right] \end{array}$$

where K(.) is the Bessel function and $E_{1}\left(.\right)$ is the exponential integral.

The remainder of this paper is organized as follows. In Section 2, we obtain maximum likelihood estimators for the Shannon and Rényi entropies and the confidence interval (CIs). Section 3 presents Bayesian estimates using different loss functions: the squared error loss function (SE), the general entropy loss function (GE), and the LINEX loss function. Due to the absence of clear formulas for the Bayes estimates, we employ Lindley’s approximation method in Section 4, while in Section 5, we use importance sampling (IS) to derive credible intervals. In Section 6, the Monte Carlo Markov Chain (MCMC) is used to determine credible intervals and the highest posterior density (HPD). The simulation study is analyzed in Section 7 with the attachment of its resulting tables and figures and commentary on the tables. As for Section 8, it presents the real data and attaches the resulting tables and figures along with their interpretation. Finally, we review the recommendations based on the simulation results and real data, as they are linked to a previous study, to illustrate the advantages.

## 2. Maximum Likelihood Estimation

Let $X=(X_{1},X_{2},\ldots ,X_{m})$ denote the progressive Type-II censored sample, with $X_{i}=X_{i:m:n}$ representing the $i^{th}$ failure time. Although the MLEs for the parameters of the IG distribution under progressive Type-II censoring have been derived previously, we briefly restate the derivation here because the subsequent entropy estimators depend directly on these MLEs through the invariance property. The likelihood function is given by:

(9) $$L\left(\mu ,\lambda |x\right)=C\prod_{i=1}^{m}f\left(x_{\left(i\right)};\mu ,\lambda \right){\left[1-F(x_{\left(i\right)};\mu ,\lambda )\right]}^{R_{i}}$$

By using Equations (3) and (4) and substituting them into Equation (9), we obtain:

(10) $$\begin{array}{c} L\left(\mu ,\lambda |x\right)\propto {\left[\frac{\lambda }{2\pi }\right]}^{\frac{m}{2}}\prod_{i=1}^{m}\frac{1}{x_{i}^{3}}\left[e^{-\sum_{i=1}^{m}B_{i}}\right]\prod_{i=1}^{m}A_{i}^{R_{i}} \end{array}$$

where $A_{i}=1-\left(\Phi \left[\sqrt{\frac{\lambda }{x_{i}}}\left(\frac{x_{i}}{\mu }-1\right)\right]+e^{\frac{2\lambda }{\mu }}\Phi \left[-\sqrt{\frac{\lambda }{x_{i}}}\left(\frac{x_{i}}{\mu }+1\right)\right]\right)$, $B_{i}=\frac{\lambda {\left(x_{i}-\mu \right)}^{2}}{2\mu^{2}x_{i}}$.

The log-likelihood function is expressed as:

(11) $$\begin{array}{c} \ell \left(\mu ,\lambda |x\right)\propto \frac{m}{2}\ln\left(\frac{\lambda }{2\pi }\right)+\sum_{i=1}^{m}\ln\left[\frac{1}{x_{i}}\right]-\sum_{i=1}^{m}B_{i}+\sum_{i=1}^{m}R_{i}\ln A_{i} \end{array}$$

We obtain the MLEs of the unknown parameters μ and λ by maximizing the log-likelihood function given in Equation (11) with respect to μ and λ, respectively. Differentiating Equation (11) with respect to μ, and then equating the result to 0, we obtain the normal equation for μ, which is given by:

(12) $$\begin{array}{c} -\sum_{i=1}^{m}{\hat{B}}_{i,\mu }+\sum_{i=1}^{m}R_{i}{\hat{A}}_{i}^{-1}\left\{\Phi \sqrt{\hat{\lambda }}\left[\frac{\sqrt{x_{i}}}{{\hat{\mu }}^{2}}(e^{\frac{2\hat{\lambda }}{\hat{\mu }}}-1)+e^{\frac{2\hat{\lambda }}{\hat{\mu }}}(\frac{2\hat{\lambda }\sqrt{x_{i}}}{{\hat{\mu }}^{3}}-\frac{2\hat{\lambda }}{{\hat{\mu }}^{2}\sqrt{x_{i}}})\right]\right\}=0 \end{array}$$

Similarly, the normal equation of λ is as follows:

(13) $$\begin{array}{c} \frac{m}{2\hat{\lambda }}-\sum_{i=1}^{m}{\hat{B}}_{i,\lambda }+\sum_{i=1}^{m}\frac{R_{i}}{{\hat{A}}_{i}}\left\{\Phi \left[\frac{1}{2\sqrt{\hat{\lambda }}}\left(\frac{\sqrt{x_{i}}}{\hat{\mu }}-\frac{1}{\sqrt{x_{i}}}\right)+e^{\frac{2\hat{\lambda }}{\hat{\mu }}}\left(\frac{2\sqrt{\hat{\lambda }}}{\hat{\mu }}+\frac{1}{2\sqrt{\hat{\lambda }}}\right)\left(\frac{1}{\sqrt{x_{i}}}-\frac{\sqrt{x_{i}}}{\hat{\mu }}\right)\right]\right\}=0 \end{array}$$

where $B_{i,\lambda }=\frac{\partial B_{i}}{\partial \lambda }$, $B_{i,\mu }=\frac{\partial B_{i}}{\partial \mu }$. We simultaneously solve Equations (12) and (13) for μ and λ to determine their maximum likelihood estimates (MLEs). Nevertheless, explicit solutions for these equations are nonexistent, indicating that the maximum likelihood estimators cannot be articulated in a closed form. We employ numerical methods to ascertain solutions to Equations (12) and (13). Let $\left(\hat{\mu },\hat{\lambda }\right)$ denote the joint maximum likelihood estimators (MLEs) of μ and λ. The Shannon and Rényi entropy measures are nonlinear functions of (μ,λ). By substituting the joint MLEs $\left(\hat{\mu },\hat{\lambda }\right)$ into these functions, we obtain the corresponding profile maximum likelihood estimators (Pr. MLEs) of the entropies as follows:

(14) $$\hat{H}=\frac{1}{2}\ln\frac{2\pi e}{\hat{\lambda }}+\frac{3}{2}\ln\hat{\mu }-\frac{3}{2}\;e^{2\hat{\lambda }/\hat{\mu }}E_{1}\left(\frac{2\hat{\lambda }}{\hat{\mu }}\right)$$

and

(15) $${\hat{H}}_{\alpha }=\frac{1}{1-\alpha }\left[\frac{\alpha }{2}\ln\frac{\hat{\lambda }}{2\pi }+\left(1-\frac{3\alpha }{2}\right)\ln\hat{\mu }+\ln2+\frac{\alpha \hat{\lambda }}{\hat{\mu }}+\ln K_{1-\frac{3\alpha }{2}}\;\left(\frac{\alpha \hat{\lambda }}{\hat{\mu }}\right)\right]$$

It is important to emphasize that these are profile MLEs (plug-in estimates) of the entropy measures, not true MLEs. This distinction is critical because profile MLEs of nonlinear functions may not inherit the optimal properties of MLEs and can exhibit bias and instability, particularly under censoring and for complex entropy measures such as Shannon entropy.

To compute the CIs for unknown parameters μ and λ, we utilize the asymptotic characteristics of the MLEs. In numerous real scenarios, precisely estimating these predicted values can prove challenging. We typically adopt a more straightforward method: we eliminate the expectation and compute the second derivatives directly, subsequently substituting the estimated values of μ and λ. This provides us with an estimated representation of the Fisher information matrix:

$$I\left(\hat{\mu },\hat{\lambda }\right)\approx {\left[\begin{array}{cc} -\frac{\partial^{2}\ln L}{\partial \mu^{2}} & -\frac{\partial^{2}\ln L}{\partial \mu \;\partial \lambda }\\ -\frac{\partial^{2}\ln L}{\partial \lambda \;\partial \mu } & -\frac{\partial^{2}\ln L}{\partial \lambda^{2}} \end{array}\right]}_{\mu =\hat{\mu },\;\lambda =\hat{\lambda }}$$

which we used to get an approximate variance-covariance matrix for the estimators. That helps us to understand how much uncertainty there is in each estimate and how the two parameters might be statistically related. The variance-covariance matrix of IG estimators is calculated as follows:

$$I\left(\hat{\mu },\hat{\lambda }\right)=-\left[\begin{array}{cc} {\hat{\ell }}_{\mu \mu } & {\hat{\ell }}_{\mu \lambda }\\ {\hat{\ell }}_{\lambda \mu } & {\hat{\ell }}_{\lambda \lambda } \end{array}\right]$$

The inverse of the matrix:

(16) $$I^{-1}=\frac{1}{{\hat{\ell }}_{\mu \mu }{\hat{\ell }}_{\lambda \lambda }-{\left({\hat{\ell }}_{\mu \lambda }\right)}^{2}}\left[\begin{array}{cc} {\hat{\ell }}_{\lambda \lambda } & -{\hat{\ell }}_{\lambda \mu }\\ -{\hat{\ell }}_{\mu \lambda } & {\hat{\ell }}_{\mu \mu } \end{array}\right]=\left[\begin{array}{cc} {\hat{\sigma }}_{\mu \mu } & -{\hat{\sigma }}_{\mu \lambda }\\ -{\hat{\sigma }}_{\lambda \mu } & {\hat{\sigma }}_{\lambda \lambda } \end{array}\right]$$

The second derivatives with respect to the parameters μ and λ are computed (Section S2),

$$\begin{array}{cc} \ell_{\mu \mu } & =\sum_{i=1}^{m}B_{i,\mu \mu }+\sum_{i=1}^{m}R_{i}\left(\frac{A_{i,\mu \mu }A_{i}-A_{i,\mu }^{2}}{A_{i}^{2}}\right),\;\ell_{\lambda \lambda }=C_{\lambda \lambda }-\sum_{i=1}^{m}B_{i,\lambda \lambda }+\sum_{i=1}^{m}R_{i}\left(\frac{A_{i,\lambda \lambda }A_{i}-A_{i,\lambda }^{2}}{A_{i}^{2}}\right),\\ \ell_{\mu \lambda } & =\sum_{i=1}^{m}B_{i,\mu \lambda }+\sum_{i=1}^{m}R_{i}\left(\frac{A_{i,\mu \lambda }A_{i}-A_{i,\mu }A_{i,\lambda }}{A_{i}^{2}}\right),\;\ell_{\lambda \mu }=C_{\lambda \mu }-\sum_{i=1}^{m}B_{i,\lambda \mu }+\sum_{i=1}^{m}R_{i}\left(\frac{A_{i,\lambda \mu }A_{i}-A_{i,\lambda }A_{i,\mu }}{A_{i}^{2}}\right). \end{array}$$

where $C=\frac{m}{2}\ln\left(\frac{\lambda }{2\pi }\right)$. Subsequently, we substitute in Matrix (16). Furthermore, we utilize the delta approach to obtain estimated confidence intervals for the Shannon and Rényi entropy metrics. For a comprehensive elucidation of this strategy, we direct the reader to Greene [35]. Assume that

$$\tau_{H}^{T}=\left(\frac{\partial H(\mu ,\lambda )}{\partial \mu },\frac{\partial H(\mu ,\lambda )}{\partial \lambda }\right)\;\mathrm{and}\;\tau_{H_{\alpha }}^{T}=\left(\frac{\partial H_{\alpha }\left(\mu ,\lambda \right)}{\partial \mu },\frac{\partial H_{\alpha }\left(\mu ,\lambda \right)}{\partial \lambda }\right).$$

Moreover, the approximate estimated variances in $\hat{H}$ and ${\hat{H}}_{\alpha }$ were given by

$$\hat{\mathrm{Var}}\left(\hat{H}\right)=\left[\tau_{H}^{T}\hat{I}{\left(\hat{\mu },\hat{\lambda }\right)}^{-1}\tau_{H}\right]\;\mathrm{and}\;\hat{\mathrm{Var}}\left({\hat{H}}_{\alpha }\right)=\left[\tau_{H_{\alpha }}^{T}\hat{I}{\left(\hat{\mu },\hat{\lambda }\right)}^{-1}\tau_{H_{\alpha }}\right].$$

Therefore, $\left[\left(\hat{H}-H\right)/\sqrt{\hat{\mathrm{Var}}\left(\hat{H}\right)}\;\right]$ and $\left[\left({\hat{H}}_{\alpha }-H_{\alpha }\right)/\sqrt{\hat{\mathrm{Var}}\left({\hat{H}}_{\alpha }\right)}\;\right]$ asymptotically followed the standard normal distribution. Thus, the asymptotic CIs (Wald interval) $100(1-\tilde{p})\%$ for the Shannon and Rényi entropies were given, respectively, by

$$\hat{H}\pm Z_{\tilde{p}/2}\sqrt{\hat{\mathrm{Var}}\left(\hat{H}\right)}\;\mathrm{and}\;{\hat{H}}_{\alpha }\pm Z_{\tilde{p}/2}\sqrt{\hat{\mathrm{Var}}\left({\hat{H}}_{\alpha }\right)}.$$

where $Z_{\tilde{p}/2}$ denoted the upper ${\left(\tilde{p}/2\right)}^{th}$ percentile of the standard normal distribution. The final result is obtained numerically because the equations involved are complex to solve analytically. It is important to note that the Wald-CIs constructed via the delta method are asymptotic in nature, and their finite sample performance depends on the shape of the likelihood function, the nonlinearity of the entropy formula, and the stability of the variance-covariance matrix. It is well documented that Wald intervals can perform poorly when the likelihood surface is irregular or the sample size is small, as exemplified by the binomial success probability [36].

Although the MLEs are straightforward to compute via the invariance principle, their finite-sample performance under progressive censoring (particularly for the entropy functions) remains to be evaluated. This motivates the development of Bayesian alternatives in the following section.

## 3. Bayesian Estimation

After deriving the MLEs of the uncertainty measures in Equations (7) and (8) in the preceding section, we now proceed to Bayesian inference for the Shannon and Rényi entropy measures. This Bayesian approach commences with the definition of a prior distribution and a likelihood function, which collectively yield the posterior distribution of the quantities of interest. Choosing an appropriate prior distribution for unknown parameters is a fundamental challenge in Bayesian inference. In the absence of strong prior knowledge, a gamma distribution was adopted as the prior for both μ and λ, consistent with prior specifications previously employed in the context of the IG distribution [37]. The specific numerical values of the prior hyperparameters, along with the corresponding sensitivity analysis, are detailed in the simulation section, where practical performance is assessed under multiple prior scenarios.

A random variable *X* is considered to adhere to a gamma distribution, assuming independent gamma priors for μ and λ, respectively, in this context:

$$g_{1}\left(\mu ;a,b\right)=\frac{b^{a}\mu^{a-1}e^{-\mu b}}{\Gamma (a)},\;\;g_{2}\left(\lambda ;c,d\right)=\frac{d^{c}\lambda^{c-1}e^{-\lambda d}}{\Gamma (c)}.\;\;\lambda >0,\;a,b,c,d>0$$

We give the joint prior distribution for μ and λ as:

(17) $$g\left(\mu ,\lambda \right)=\frac{b^{a}d^{c}}{\Gamma (a)\Gamma (c)}\left(\mu^{a-1}\right)\left(\lambda^{c-1}\right)\left(e^{-(\mu b+\lambda d)}\right)\;\;\;\mu ,\lambda >0,\;a,b,c,d>0$$

We take the log-joint prior distribution for μ and λ as:

(18) ρ(μ,λ)=aln(b)+cln(d)+(a−1)ln(μ)+(c−1)ln(λ)−μb−λd−lnΓ(a)−lnΓ(c)

where ρ(μ,λ)=lng(μ,λ). After making calculations using Equations (10) and (17), the joint posterior distribution of μ and λ can be established as follows:

(19) $$\Pi \left(\mu ,\lambda |\underline{x}\right)=\frac{1}{\tilde{z}}(\mu^{a-1})(\lambda^{\frac{m}{2}+c-1})(e^{-(\mu b+\lambda d)})\prod_{i=1}^{m}\sqrt{\frac{1}{x_{i}^{3}}}\;e^{-B_{i}}\;A_{i}^{R_{i}}$$

where $\tilde{z}$ is a normalizing constant defined by,

$$\tilde{z}=\int_{0}^{\infty }\int_{0}^{\infty }(\mu^{a-1})(\lambda^{\frac{m}{2}+c-1})(e^{-(\mu b+\lambda d)})\prod_{i=1}^{m}\sqrt{\frac{1}{x_{i}^{3}}}\;e^{-B_{i}}\;A_{i}^{R_{i}}\;d\mu \;d\lambda$$

The posterior density function of μ,λ is determined by Bayes’ method:

(20) $$\Pi \left(\mu ,\lambda |\underline{x}\right)=\frac{L(\mu ,\lambda )\;g(\mu ,\lambda )}{\int_{\left(\mu ,\lambda \right)}L\left(\mu ,\lambda \right)\;g\left(\mu ,\lambda \right)d\left(\mu ,\lambda \right)}$$

This paper analyzes three forms of loss function: the SE loss function, the GE loss function, and the LINEX loss function. Let $\hat{H}$ denote an estimator for the entropy functions *H*. Subsequently, we develop the loss functions, as illustrated in Table 1.

The Bayes estimator under loss function can be expressed as:

(21) $${\hat{H}}_{B}=T^{-1}\left(E\left[ð\left(H\right)|\underline{x}\right]\right)=T^{-1}\left(\int_{\left(\mu ,\lambda \right)}ð\left(H\right)\;\Pi \left(\mu ,\lambda |\underline{x}\right)\;d\left(\mu ,\lambda \right)\right)$$

where ð(H) is the transformation applied to the entropy before taking the posterior expectation, and $T^{-1}$ is the inverse operation that recovers ${\hat{H}}_{B}$ from the expected transformed value. For SE loss function, ð(H)=H and $T^{-1}$ is the identity. For GE loss function, $ð\left(H\right)=H^{-q}$ and $T^{-1}\left(x\right)=x^{-1/q}$. For LINEX loss function, $ð\left(H\right)=e^{-pH}$ and $T^{-1}\left(x\right)=-\frac{1}{p}\ln\left(x\right)$.

Using Equation (21) and Equation (19), the Bayes estimates of the Shannon entropy *H* with respect to the GE and LINEX loss functions are obtained, respectively, as follows:

(22) $$H={\left[\frac{1}{\tilde{z}}\int_{0}^{\infty }\int_{0}^{\infty }{\left(H\right)}^{-q}\left(\mu^{a-1}\right)(\lambda^{\frac{m}{2}+c-1})(e^{-(\mu b+\lambda d)})\prod_{i=1}^{m}\sqrt{\frac{1}{x_{i}^{3}}}\;A_{i}^{R_{i}}\;d\mu \;d\lambda \right]}^{-\frac{1}{q}}$$

(23) $$H=-\frac{1}{p}\ln\left[\frac{1}{\tilde{z}}\int_{0}^{\infty }\int_{0}^{\infty }\left(\mu^{a-1}\right)\left(\lambda^{\frac{m}{2}+c-1}\right)\left(e^{-(\mu b+\lambda d+p\;H)}\right)\prod_{i=1}^{m}\sqrt{\frac{1}{x_{i}^{3}}}\;A_{i}^{R_{i}}\;d\mu \;d\lambda \right]$$

By replacing q=−1 into Equation (22), we obtain the Bayes estimate of Shannon entropy *H* concerning the SE loss function. Likewise, we employ Equation (21) and Equation (19) to derive the Bayes estimates of the Rényi entropy $H_{\alpha }$ based on the aforementioned loss functions.

Computing closed-form solutions for the ratio of the two integrals presented in Equations (22) and (23) is intricate. To tackle this issue, we utilize Lindley’s approximation technique.

## 4. Lindley’s Approximation

In this section, we derive Lindley’s approximation for Bayesian estimates, which depend on two parameters, μ and λ. The main equation was proposed by Lindley [38]. It is expressed as follows:

(24) $$I\left(x\right)=E\left[u\left(\mu ,\lambda |\underline{x}\right)\right]=\frac{\int_{\left(\mu ,\lambda \right)}u\left(\mu ,\lambda \right)e^{\ell (\mu ,\lambda )+\rho (\mu ,\lambda )}d\left(\mu ,\lambda \right)}{\int_{\left(\mu ,\lambda \right)}e^{\ell (\mu ,\lambda )+\rho (\mu ,\lambda )}d\left(\mu ,\lambda \right)}$$

where

$$\begin{array}{cc} u(\mu ,\lambda )=\mathrm{The}\;\mathrm{objective}\;\mathrm{function}\;\mathrm{of}\;\mu \;\mathrm{and}\;\lambda ,\;\ell (\mu ,\lambda ) & =\mathrm{The}\;\log\;\mathrm{of}\;\mathrm{likelihood}\;\mathrm{function},\\ \rho (\mu ,\lambda )=\mathrm{The}\;\log\;\mathrm{of}\;\mathrm{joint}\;\mathrm{prior}\;\mathrm{of}\; & \mu \;\mathrm{and}\;\lambda . \end{array}$$

can be evaluated as

(25) $$\begin{array}{c} \;I\left(x\right)=u\left(\hat{\mu },\hat{\lambda }\right)+\frac{1}{2}\left[\left({\hat{u}}_{\lambda \lambda }+2{\hat{u}}_{\lambda }{\hat{\rho }}_{\lambda }\right){\hat{\sigma }}_{\lambda \lambda }+\left({\hat{u}}_{\mu \lambda }+2{\hat{u}}_{\mu }{\hat{\rho }}_{\lambda }\right){\hat{\sigma }}_{\mu \lambda }+\left({\hat{u}}_{\lambda \mu }+2{\hat{u}}_{\lambda }{\hat{\rho }}_{\mu }\right){\hat{\sigma }}_{\lambda \mu }+\left({\hat{u}}_{\mu \mu }+2{\hat{u}}_{\mu }{\hat{\rho }}_{\mu }\right){\hat{\sigma }}_{\mu \mu }\right]\\ \;+\frac{1}{2}[\left({\hat{u}}_{\lambda }{\hat{\sigma }}_{\lambda \lambda }+{\hat{u}}_{\mu }{\hat{\sigma }}_{\lambda \mu }\right)\left({\hat{\ell }}_{\lambda \lambda \lambda }{\hat{\sigma }}_{\lambda \lambda }+{\hat{\ell }}_{\lambda \mu \lambda }{\hat{\sigma }}_{\lambda \mu }+{\hat{\ell }}_{\mu \lambda \lambda }{\hat{\sigma }}_{\mu \lambda }+{\hat{\ell }}_{\mu \mu \lambda }{\hat{\sigma }}_{\mu \mu }\right)+\left({\hat{u}}_{\lambda }{\hat{\sigma }}_{\mu \lambda }+{\hat{u}}_{\mu }{\hat{\sigma }}_{\mu \mu }\right)\\ \left({\hat{\ell }}_{\mu \lambda \lambda }{\hat{\sigma }}_{\lambda \lambda }+{\hat{\ell }}_{\lambda \mu \mu }{\hat{\sigma }}_{\lambda \mu }+{\hat{\ell }}_{\mu \lambda \mu }{\hat{\sigma }}_{\mu \lambda }+{\hat{\ell }}_{\mu \mu \mu }{\hat{\sigma }}_{\mu \mu })\right] \end{array}$$

where

$$\begin{array}{cccccccccc} \hat{\mu } & =\mathrm{MLE}\;\mathrm{of}\;\mu ,\; & \hat{\lambda } & =\mathrm{MLE}\;\mathrm{of}\;\lambda ,\; & {\hat{u}}_{\lambda } & =\frac{\partial u(\hat{\mu },\hat{\lambda })}{\partial \hat{\lambda }},\; & {\hat{u}}_{\mu } & =\frac{\partial u(\hat{\mu },\hat{\lambda })}{\partial \hat{\mu }},\; & {\hat{u}}_{\lambda \lambda } & =\frac{\partial^{2}u\left(\hat{\mu },\hat{\lambda }\right)}{\partial {\hat{\lambda }}^{2}},\\ {\hat{u}}_{\mu \mu } & =\frac{\partial^{2}u\left(\hat{\mu },\hat{\lambda }\right)}{\partial {\hat{\mu }}^{2}},\; & {\hat{u}}_{\lambda \mu } & =\frac{\partial^{2}u\left(\hat{\mu },\hat{\lambda }\right)}{\partial \hat{\lambda }\;\partial \hat{\mu }},\; & {\hat{u}}_{\mu \lambda } & =\frac{\partial^{2}u\left(\hat{\mu },\hat{\lambda }\right)}{\partial \hat{\mu }\;\partial \hat{\lambda }},\; & {\hat{\ell }}_{\lambda \lambda \mu } & =\frac{\partial^{3}\ell \left(\hat{\mu },\hat{\lambda }\right)}{\partial {\hat{\lambda }}^{2}\;\partial \hat{\mu }},\; & {\hat{\ell }}_{\lambda \lambda \lambda } & =\frac{\partial^{3}\ell \left(\hat{\mu },\hat{\lambda }\right)}{\partial {\hat{\lambda }}^{3}},\\ {\hat{\ell }}_{\lambda \mu \lambda } & =\frac{\partial^{3}\ell \left(\hat{\mu },\hat{\lambda }\right)}{\partial \hat{\lambda }\;\partial \hat{\mu }\;\partial \hat{\lambda }},\; & {\hat{\ell }}_{\mu \mu \lambda } & =\frac{\partial^{3}\ell \left(\hat{\mu },\hat{\lambda }\right)}{\partial {\hat{\mu }}^{2}\;\partial \hat{\lambda }},\; & {\hat{\ell }}_{\mu \lambda \lambda } & =\frac{\partial^{3}\ell \left(\hat{\mu },\hat{\lambda }\right)}{\partial \hat{\mu }\;\partial {\hat{\lambda }}^{2}},\; & {\hat{\ell }}_{\lambda \mu \mu } & =\frac{\partial^{3}\ell \left(\hat{\mu },\hat{\lambda }\right)}{\partial \hat{\lambda }\;\partial {\hat{\mu }}^{2}},\; & {\hat{\ell }}_{\mu \mu \mu } & =\frac{\partial^{3}\ell \left(\hat{\mu },\hat{\lambda }\right)}{\partial {\hat{\mu }}^{3}},\\ {\hat{\ell }}_{\mu \lambda \mu } & =\frac{\partial^{3}\ell \left(\hat{\mu },\hat{\lambda }\right)}{\partial \hat{\mu }\;\partial \hat{\lambda }\;\partial \hat{\mu }},\; & {\hat{\rho }}_{\lambda } & =\frac{\partial \rho (\hat{\mu },\hat{\lambda })}{\partial \hat{\lambda }},\; & {\hat{\rho }}_{\mu } & =\frac{\partial \rho (\hat{\mu },\hat{\lambda })}{\partial \hat{\mu }}. & & & & \end{array}$$

That approximation method has been employed by numerous researchers, including Nassar et al. [39], Kundu and Pradhan [40], Xu et al. [41], Kim et al. [42], Sultan and Ahmad [43], Sana and Faizan [44], and Ren and Hu [45].

⇒ Firstly;  To apply Lindley’s approximation, the function u(μ,λ) appearing in the posterior expectation must be specified for each loss function. From the general form given in Equation (21), we obtain the following (detailed derivations and the required partial derivatives are provided in Section S2):

- Bayesian estimators of the Shannon entropy under the SE, GE, and LINEX loss function, respectively, as follows:
  $$u_{\mathrm{SE}}\left(\mu ,\lambda \right)=H,\;u_{\mathrm{GE}}\left(\mu ,\lambda \right)=H^{-q}\;\;\mathrm{and}\;\;u_{\mathrm{LINEX}}\left(\mu ,\lambda \right)=e^{-H\;p}.$$
- Bayesian estimators of the Rényi entropy under the SE, GE, and LINEX loss function, respectively, as follows:
  $$u_{\mathrm{SE}}\left(\mu ,\lambda \right)=H_{\alpha },\;u_{\mathrm{GE}}\left(\mu ,\lambda \right)=H_{\alpha }^{-q}\;\;\mathrm{and}\;\;u_{\mathrm{LINEX}}\left(\mu ,\lambda \right)=e^{-H_{\alpha }\;p}.$$

⇒ Secondly;  we obtain the derivatives of the ML equations (in Section S2):
  $$\begin{array}{ccc} {\hat{\ell }}_{\mu \mu \mu } & = & \sum_{i=1}^{m}B_{i,\mu \mu \mu }+\sum_{i=1}^{m}R_{i}\left[\frac{A_{i,\mu \mu \mu }A_{i}^{2}-3A_{i,\mu }A_{i,\mu \mu }A_{i}+2A_{i,\mu }^{3}}{A_{i}^{3}}\right]\\ {\hat{\ell }}_{\mu \mu \lambda } & = & \sum_{i=1}^{m}B_{i,\mu \mu \lambda }+\sum_{i=1}^{m}R_{i}\left[\frac{A_{i,\mu \mu \lambda }A_{i}^{2}-A_{i}A_{i,\mu \mu }A_{i,\lambda }-2A_{i}A_{i,\mu }A_{i,\mu \lambda }+2A_{i,\mu }^{2}A_{i,\lambda }}{A_{i}^{3}}\right]\\ {\hat{\ell }}_{\mu \lambda \mu } & = & \sum_{i=1}^{m}B_{i,\mu \lambda \mu }+\sum_{i=1}^{m}R_{i}\left[\frac{A_{i,\mu \lambda \mu }A_{i}^{2}-2A_{i}A_{i,\mu }A_{i,\mu \lambda }-A_{i}A_{i,\mu \mu }A_{i,\lambda }+2A_{i,\mu }^{2}A_{i,\lambda }}{A_{i}^{3}}\right]\\ {\hat{\ell }}_{\mu \lambda \lambda } & = & \sum_{i=1}^{m}R_{i}\left[\frac{A_{i,\mu \lambda \lambda }A_{i}^{2}-2A_{i}A_{i,\mu \lambda }A_{i,\lambda }-A_{i}A_{i,\mu }A_{i,\lambda \lambda }+2A_{i,\mu }A_{i,\lambda }^{2}}{A_{i}^{3}}\right]\\ {\hat{\ell }}_{\lambda \lambda \lambda } & = & C_{\lambda \lambda \lambda }+\sum_{i=1}^{m}R_{i}\left[\frac{A_{i,\lambda \lambda \lambda }A_{i}^{2}-3A_{i,\lambda }A_{i,\lambda \lambda }A_{i}+2A_{i,\lambda }^{3}}{A_{i}^{3}}\right]\\ {\hat{\ell }}_{\lambda \lambda \mu } & = & \sum_{i=1}^{m}R_{i}\left[\frac{A_{i,\lambda \lambda \mu }A_{i}^{2}-A_{i,\lambda \lambda }A_{i,\mu }A_{i}-2A_{i,\lambda \mu }A_{i,\lambda }A_{i}+2A_{i,\lambda }^{2}A_{i,\mu }}{A_{i}^{3}}\right]\\ {\hat{\ell }}_{\lambda \mu \lambda } & = & \sum_{i=1}^{m}R_{i}\left[\frac{A_{i,\lambda \lambda \mu }A_{i}^{2}-A_{i,\lambda \lambda }A_{i,\mu }A_{i}-2A_{i,\lambda \mu }A_{i,\lambda }A_{i}+2A_{i,\lambda }^{2}A_{i,\mu }}{A_{i}^{3}}\right]\\ {\hat{\ell }}_{\lambda \mu \mu } & = & \;-\sum_{i=1}^{m}B_{i,\lambda \mu \mu }+\sum_{i=1}^{m}R_{i}\left[\frac{A_{i,\lambda \mu \mu }A_{i}^{2}-A_{i,\lambda }A_{i,\mu \mu }A_{i}-2A_{i,\lambda \mu }A_{i,\mu }A_{i}+2A_{i,\lambda }A_{i,\mu }^{2}}{A_{i}^{3}}\right] \end{array}$$
⇒ Thirdly;  we obtain the derivatives of the log-joint prior distribution from Equation (18):
  $${\hat{\rho }}_{\mu }=\frac{a-1}{\mu }-b,\;{\hat{\rho }}_{\lambda }=\frac{c-1}{\lambda }-d.$$
⇒ Fourthly;  we substitute it into Equation (25) to obtain the result. We change the objective function ($u(\hat{\mu },\hat{\lambda })$) and its derivatives (${\hat{u}}_{\lambda }$, ${\hat{u}}_{\mu }$, ${\hat{u}}_{\lambda \lambda }$, ${\hat{u}}_{\mu \mu }$, ${\hat{u}}_{\lambda \mu }$ and ${\hat{u}}_{\mu \lambda }$) each time due to the different loss functions every time, while keeping the other derivatives related to the maximum likelihood function (${\hat{\ell }}_{\mu \mu \mu }$, ${\hat{\ell }}_{\mu \mu \lambda }$, ${\hat{\ell }}_{\mu \lambda \mu }$, ${\hat{\ell }}_{\mu \lambda \lambda }$, ${\hat{\ell }}_{\lambda \lambda \lambda }$, ${\hat{\ell }}_{\lambda \lambda \mu }$, ${\hat{\ell }}_{\lambda \mu \lambda }$ and ${\hat{\ell }}_{\lambda \mu \mu }$), Fisher matrix elements (${\hat{\sigma }}_{\lambda \lambda }$, ${\hat{\sigma }}_{\mu \lambda }$, ${\hat{\sigma }}_{\lambda \mu }$, and ${\hat{\sigma }}_{\mu \mu }$) and also the derivatives of joint prior distribution (${\hat{\rho }}_{\lambda }$ and ${\hat{\rho }}_{\mu }$) fixed.

Thus, the Bayes estimate of Shannon entropy *H* with respect to the GE loss function is obtained as;

(26) $$\begin{array}{c} {\left[E\left({\left[H\right]}^{-q}|\underline{x}\right)\right]}^{-\frac{1}{q}}=[H^{-q}+\frac{1}{2}[\left({\hat{u}}_{\lambda \lambda }+2{\hat{u}}_{\lambda }{\hat{\rho }}_{\lambda }\right){\hat{\sigma }}_{\lambda \lambda }+\left({\hat{u}}_{\mu \lambda }+2{\hat{u}}_{\mu }{\hat{\rho }}_{\lambda }\right){\hat{\sigma }}_{\mu \lambda }+\left({\hat{u}}_{\lambda \mu }+2{\hat{u}}_{\lambda }{\hat{\rho }}_{\mu }\right){\hat{\sigma }}_{\lambda \mu }\\ \;+\left({\hat{u}}_{\mu \mu }+2{\hat{u}}_{\mu }{\hat{\rho }}_{\mu }\right){\hat{\sigma }}_{\mu \mu }]+\frac{1}{2}[\left({\hat{u}}_{\lambda }{\hat{\sigma }}_{\lambda \lambda }+{\hat{u}}_{\mu }{\hat{\sigma }}_{\lambda \mu }\right)\left({\hat{\ell }}_{\lambda \lambda \lambda }{\hat{\sigma }}_{\lambda \lambda }+{\hat{\ell }}_{\lambda \mu \lambda }{\hat{\sigma }}_{\lambda \mu }+{\hat{\ell }}_{\mu \lambda \lambda }{\hat{\sigma }}_{\mu \lambda }+{\hat{\ell }}_{\mu \mu \lambda }{\hat{\sigma }}_{\mu \mu }\right)\\ +{\left({\hat{u}}_{\lambda }{\hat{\sigma }}_{\mu \lambda }+{\hat{u}}_{\mu }{\hat{\sigma }}_{\mu \mu }\right)\left({\hat{\ell }}_{\mu \lambda \lambda }{\hat{\sigma }}_{\lambda \lambda }+{\hat{\ell }}_{\lambda \mu \mu }{\hat{\sigma }}_{\lambda \mu }+{\hat{\ell }}_{\mu \lambda \mu }{\hat{\sigma }}_{\mu \lambda }+{\hat{\ell }}_{\mu \mu \mu }{\hat{\sigma }}_{\mu \mu }\right)]]}^{-\frac{1}{q}} \end{array}$$

By substituting q=−1 in Equation (26), we can obtain the Bayes estimate of *H* with respect to the SE loss function. Using a similar procedure, we can obtain the Bayes estimates of the Rényi entropy $H_{\alpha }$ with respect to the above loss functions.

Therefore, the Bayes estimate of Shannon entropy *H* with respect to the LINEX loss function is obtained as;

(27) $$\begin{array}{c} \left[E\left(e^{-[H]p}|\underline{x}\right)\right]=\frac{1}{p}\ln[e^{-H\;p}+\frac{1}{2}[\left({\hat{u}}_{\lambda \lambda }+2{\hat{u}}_{\lambda }{\hat{\rho }}_{\lambda }\right){\hat{\sigma }}_{\lambda \lambda }+\left({\hat{u}}_{\mu \lambda }+2{\hat{u}}_{\mu }{\hat{\rho }}_{\lambda }\right){\hat{\sigma }}_{\mu \lambda }+\left({\hat{u}}_{\lambda \mu }+2{\hat{u}}_{\lambda }{\hat{\rho }}_{\mu }\right){\hat{\sigma }}_{\lambda \mu }\\ \;+\left({\hat{u}}_{\mu \mu }+2{\hat{u}}_{\mu }{\hat{\rho }}_{\mu }\right){\hat{\sigma }}_{\mu \mu }]+\frac{1}{2}[\left({\hat{u}}_{\lambda }{\hat{\sigma }}_{\lambda \lambda }+{\hat{u}}_{\mu }{\hat{\sigma }}_{\lambda \mu }\right)\left({\hat{\ell }}_{\lambda \lambda \lambda }{\hat{\sigma }}_{\lambda \lambda }+{\hat{\ell }}_{\lambda \mu \lambda }{\hat{\sigma }}_{\lambda \mu }+{\hat{\ell }}_{\mu \lambda \lambda }{\hat{\sigma }}_{\mu \lambda }+{\hat{\ell }}_{\mu \mu \lambda }{\hat{\sigma }}_{\mu \mu }\right)\\ +\left({\hat{u}}_{\lambda }{\hat{\sigma }}_{\mu \lambda }+{\hat{u}}_{\mu }{\hat{\sigma }}_{\mu \mu }\right)\left({\hat{\ell }}_{\mu \lambda \lambda }{\hat{\sigma }}_{\lambda \lambda }+{\hat{\ell }}_{\lambda \mu \mu }{\hat{\sigma }}_{\lambda \mu }+{\hat{\ell }}_{\mu \lambda \mu }{\hat{\sigma }}_{\mu \lambda }+{\hat{\ell }}_{\mu \mu \mu }{\hat{\sigma }}_{\mu \mu }\right)]] \end{array}$$

Using a similar procedure, we can obtain the Bayes estimate of the Rényi entropy $H_{\alpha }$ with respect to the above loss function by using Equation (27).

Although Lindley’s approximation provides a computationally efficient way to obtain Bayesian estimates, it does not easily produce credible intervals for entropy measures. So, we now introduce the importance sampling procedure, which makes it easy to build HPD in addition to point estimates.

## 5. Importance Sampling

This paper examines the importance sampling approach for calculating Bayes estimates of unknown parametric functions as defined in Equations (7) and (8). The exception in IG is that we need to set a different proposal for each parameter μ and λ, while in most classical distributions, a conjugate distribution is enough to cover everything. First, we define the proposal distribution (p) from which we generate the parameters related to the IG μ and λ. Note that it is not necessary for the proposal distribution to be the same as the prior distribution. We need to determine the distribution of the proposal using the joint posterior distribution of μ and λ as follows:

$$\Pi \left(\mu ,\lambda |\underline{x}\right)\propto \mu^{a-1}e^{-\mu b}\;\lambda^{\frac{m}{2}+c-1}e^{-\lambda d}\;e^{-\sum_{i=1}^{m}B_{i}}\prod_{i=1}^{m}\sqrt{\frac{1}{x_{i}^{3}}}\;A_{i}^{R_{i}}$$

We focus on the parts that contain λ:

$$\Pi \left(\mu ,\lambda |\underline{x}\right)\propto \lambda^{\frac{m}{2}+c-1}\;\exp\left[(-\lambda \left(d+\frac{1}{2}\sum_{i=1}^{m}\frac{{\left(x_{i}-\mu \right)}^{2}}{\mu^{2}x_{i}}\right)\right]\;\Rightarrow \lambda >0$$

This represents the form of the gamma distribution, so the most suitable proposal distribution $\left(p_{\lambda }\right)$ for λ is the gamma distribution (G).

We focus on the parts that contain μ:

$$\Pi \left(\mu ,\lambda |\underline{x}\right)\propto \mu^{a-1}\;\exp\left(-\mu b-\frac{\lambda }{2}\sum_{i=1}^{m}\frac{{\left(x_{i}-\mu \right)}^{2}}{2\mu^{2}x_{i}}\right)\;\Rightarrow \mu >0$$

It seems that μ is more complicated because it contains this specific part ${\left(x-\mu \right)}^{2}/2\mu^{2}x$, since it does not resemble the gamma distribution. Instead, the log-posterior density can be locally approximated in the neighborhood of the maximum likelihood estimator $\hat{\mu }$ using a second-order Taylor expansion. Consequently, the posterior distribution is approximately normal around $\hat{\mu }$, which motivates the use of a normal proposal distribution. To make it clear, we follow these steps:

$$L\left(\mu \right)\propto \mu^{a-1}\;\exp\left(-\mu b-\frac{\lambda }{2}\sum_{i=1}^{m}\frac{{\left(x_{i}-\mu \right)}^{2}}{2\mu^{2}x_{i}}\right)$$

We take the logarithm:

$$\begin{array}{cc} \ell (\mu )=\ln L(\mu ) & =\left(a-1\right)\ln\left(\mu \right)-\mu b-\frac{\lambda }{2}+\frac{1}{2\mu^{2}}\sum_{i=1}^{m}x_{i}-\frac{m}{\mu }+\frac{1}{2}\sum_{i=1}^{m}\frac{1}{x_{i}} \end{array}$$

We find the first derivative and equating it to zero:

$$\begin{array}{cc} \frac{d\;\ell (\mu )}{d\mu }=\frac{a-1}{\mu }-b-\frac{\sum_{i=1}^{m}x_{i}}{\mu^{3}}+\frac{m}{\mu^{2}}\;\Rightarrow \;\frac{a-1}{\mu }-b-\frac{\sum_{i=1}^{m}x_{i}}{\mu^{3}}+\frac{m}{\mu^{2}} & =0\\ m\;\mu -\sum_{i=1}^{m}x_{i}=\mu^{2}\;(b\;\mu & -(a-1))\;\to ➀ \end{array}$$

We find the second derivative to determine the maximum and minimum values:

$$\frac{d^{2}\;\ell \left(\mu \right)}{d\mu^{2}}=-\frac{a-1}{\mu^{2}}+\frac{m}{\mu^{3}}-\frac{3\left[m\mu -\sum_{i=1}^{m}x_{i}\right]}{\mu^{4}}\;\;\to ➁$$

from ➀ and ➁

$$\begin{array}{cc} ∴\frac{d^{2}\;\ell \left(\mu \right)}{d\mu^{2}} & =\frac{2\left(a-1\right)\;\mu -3b\mu^{2}+m}{\mu^{3}} \end{array}$$

If $\left[\;{\left.\frac{d^{2}\ell \left(\mu \right)}{d\mu^{2}}\right|}_{\mu =\hat{\mu }}>0\;\right]$ means that point is a minimum, then $\left[\;{\left.\frac{d^{2}\ell \left(\mu \right)}{d\mu^{2}}\right|}_{\mu =\hat{\mu }}<0\;\right]$ also means that point is a maximum (saddle point).

Looking at the previous expression $\left(2\left(a-1\right)\;\mu -3b\mu^{2}+m\right)/\mu^{3}$, what determines whether it is positive or negative is the numerator $\left(2\left(a-1\right)\;\mu -3b\mu^{2}+m\right)$, because the denominator $\mu^{3}$ is always positive anyway. So, we plug in numbers into the numerator and look for the values that make it negative so the curve goes down, which means that point is a maximum. The larger the value of the quantity $3b\mu^{2}$, the more negative the second derivative.

Because the functional form of the proposal distribution $\left(p_{\mu }\right)$ for μ is unknown, we use Laplace approximation to determine an appropriate Gaussian form, where the logarithm of the likelihood function is expanded around the maximum likelihood estimate of μ using a second-order Taylor series, which leads to a normal approximation of the proposal distribution given by $\mu ∼N\left(\hat{\mu },{\left[I\left(\hat{\mu }\right)\right]}^{-1}\right)$, where $I(\hat{\mu })$ denotes the observed Fisher information. This approximation provides a convenient and efficient proposal distribution for the importance sampling procedure.

However, since μ>0, it is more appropriate to propose a truncated normal distribution because the standard normal distribution extends from [−∞,∞], so if $\mu ∼N(\mu_{0},\sigma^{2})$, it could take a positive or negative value, which would be illogical in this context. Therefore, $\mu ∼\mathrm{Trunc}.\mathrm{Normal}\;(\mu_{0},\sigma^{2},A,B)$ where [A,B] represents the truncation limits (that is, values outside the interval are not possible). In this approach, it is necessary to reformulate the joint posterior distribution of μ and λ as presented in Equation (19). It is provided by

$$\Pi \left(\mu ,\lambda |\underline{x}\right)\;\propto \;\left[G_{\lambda }\left(\frac{m}{2}+c\;,\;d+\frac{1}{2}\sum_{i=1}^{m}\frac{{\left(x_{i}-\mu \right)}^{2}}{\mu^{2}x_{i}}\right)\right]\;\left[N_{\mu |\lambda }{\left(\hat{\mu },{\left[I\left(\hat{\mu }\right)\right]}^{-1}\right)}_{\left[A,B\right]}\right]\;w\left(\mu ,\lambda \right)$$

For each pair generated $\left(\mu_{i},\lambda_{i}\right)$, the importance weight is computed as

$$w_{i}=\frac{L\left(x|\mu_{i},\lambda_{i}\right)\;g\left(\mu_{i},\lambda_{i}\right)}{p_{\mu }\left(\mu_{i}\right)\;p_{\lambda }\left(\lambda_{i}\right)}$$

where $L\left(x|\mu_{i},\lambda_{i}\right)\;g\left(\mu_{i},\lambda_{i}\right)=\mathrm{Posterior}\left(\mu_{i},\lambda_{i}\right)$ (before normalization). The normalized weights are then obtained as

$${\tilde{w}}_{i}=\frac{w_{i}}{\sum_{j=1}^{N}w_{j}}$$

Later, we will use these weights to calculate Bayesian estimates of any parametric function h(μ,λ) as

$${\hat{I}}_{\mathrm{weight}}=\sum_{i=1}^{N}{\tilde{w}}_{i}\;h\left(\mu_{i},\lambda_{i}\right)$$

The following procedure can be used to obtain Bayes estimates of a function, denoted as g(μ,λ), with respect to the GE and LINEX loss functions:

Step 1. Generate λ from a gamma distribution with shape parameter $\left(\frac{m}{2}+c\right)$ and scale parameter $\left(d+\frac{1}{2}\sum_{i=1}^{m}\frac{{\left(x_{i}-\mu \right)}^{2}}{\mu^{2}x_{i}}\right)$, denoted as $\left[G_{\lambda }\left(\frac{m}{2}+c\;,\;d+\frac{1}{2}\sum_{i=1}^{m}\frac{{\left(x_{i}-\mu \right)}^{2}}{\mu^{2}x_{i}}\right)\right]$.
Step 2. For the given λ obtained in Step 1., approximate the MLE round for μ, the form approaching $\left[N_{\mu |\lambda }{\left(\hat{\mu },{\left[I\left(\hat{\mu }\right)\right]}^{-1}\right)}_{\left[A,B\right]}\right]$.
Step 3. Execute Step 1. and Step 2. for *N* iterations to derive $\left(\mu_{1},\lambda_{1}\right),\left(\mu_{2},\lambda_{2}\right)$, $\ldots ,(\mu_{N},\lambda_{N})$.
Step 4. The Bayes estimates of the parametric function h(μ,λ) under the GE and LINEX loss functions are derived, respectively, as follows:
  (28) $${\hat{I}}_{\mathrm{GE}}={\left\{\sum_{i=1}^{N}h{\left(\mu_{i},\lambda_{i}\right)}^{-q}\;w\left(\mu_{i},\lambda_{i}\right)\right\}}^{-\frac{1}{q}}{\left(\sum_{i=1}^{N}w\left(\mu_{i},\lambda_{i}\right)\right)}^{\frac{1}{q}}$$
  and
  (29) $${\hat{I}}_{\mathrm{LINEX}}=-\frac{1}{p}\ln\left[\left(\sum_{i=1}^{N}e^{-p\;h(\mu_{i},\lambda_{i})}\;w\left(\mu_{i},\lambda_{i}\right)\right){\left(\sum_{i=1}^{N}w\left(\mu_{i},\lambda_{i}\right)\right)}^{-1}\right]$$
  The Bayes estimate of h(μ,λ) concerning the SE loss function ${\hat{I}}_{\mathrm{SE}}$ can be obtained from Equation (28) for q=−1. Furthermore, within the framework of the GE loss function, Bayesian estimates of the Shannon and Rényi entropy functions can be derived from Equation (28). Similarly, for the LINEX loss function, Bayesian estimates of the Shannon and Rényi entropy functions can be obtained from Equation (29), where h(μ,λ)=H corresponds to the Shannon entropy and $h\left(\mu ,\lambda \right)=H_{\alpha }$ pertains to the Rényi entropy.
Step 5. Propose intervals for the estimation of the Shannon entropy function and the Rényi entropy function. This method was used by Chen and Shao [46] for this purpose. Define
  $$\eta_{i}=\eta \left(\mu^{\left(i\right)},\lambda^{\left(i\right)}\right)$$
  and
  $$w_{i}=\frac{\mathrm{Posterior}(\mu^{\left(i\right)},\lambda^{\left(i\right)})}{p(\mu^{\left(i\right)},\lambda^{\left(i\right)})}\;\Rightarrow \;{\tilde{w}}_{i}=\frac{w(\mu^{\left(i\right)},\lambda^{\left(i\right)})}{\sum_{i=1}^{N}w\left(\mu^{\left(i\right)},\lambda^{\left(i\right)}\right)}$$
  where $\lambda^{\left(i\right)}$ and $\mu^{\left(i\right)}$ for i=1,2,…,N are posterior samples generated from previous steps for λ and μ, respectively. Each sample represents a part of the posterior distribution, and the size of that part (its proportion) is given by the normalized weight, ${\tilde{w}}_{i}$.
Step 6. Estimate the $\gamma^{th}$ quantile of $\eta_{i}=\eta \left(\mu^{\left(i\right)},\lambda^{\left(i\right)}\right)$:
  (30) $${\hat{\eta }}^{\left(\gamma \right)}=\left\{\begin{array}{cc} \eta_{\left(1\right)}, & \gamma =0\;\\ \eta_{\left(i\right)}, & \sum_{j=1}^{i-1}{\tilde{w}}_{\left(j\right)}<\gamma \leq \sum_{j=1}^{i}{\tilde{w}}_{\left(j\right)} \end{array}\right.$$
  Let $\eta_{\left(i\right)}$ be the ordered values of $\eta_{i}$, and let ${\tilde{w}}_{\left(j\right)}$ be the corresponding normalized weights for these ordered values.
Step 7. Establish the typical credible interval with a $100(1-\tilde{p})\%$ confidence level: Let *p* be the target coverage probability (the confidence level), where $p=1-\tilde{p}$. For a 95% credible interval, p=0.95 and $\tilde{p}=0.05$. The bottom bound of the credible interval is the $\tilde{p}/2$ quantile of the posterior distribution of η. The top limit of the credible interval is the $1-\tilde{p}/2$ quantile of the posterior distribution of η. The standard credible interval, sometimes referred to as the equal-tail interval, is computed utilizing the quantile estimations from Equation (30).
  (31) $$\left({\hat{\eta }}^{\left(\tilde{p}/2\right)},\;{\hat{\eta }}^{\left(1-\tilde{p}/2\right)}\right)$$
  where, ${\hat{\eta }}^{\left(\tilde{p}/2\right)}$ is the estimated quantile below which $100(\tilde{p}/2)\%$ of the posterior distribution of the entropy lies. ${\hat{\eta }}^{\left(1-\tilde{p}/2\right)}$ is the estimated quantile below which $100(1-\tilde{p}/2)\%$ of the posterior distribution lies.

Although importance sampling provides accurate estimates when the proposal distribution is well-chosen, it does not automatically explore the full posterior. We therefore also employ MCMC, which constructs a Markov chain targeting the exact posterior and serves as the gold-standard simulation-based method in this study.

## 6. Monte Carlo Markov Chain

MCMC algorithms offer a versatile approach to create approximate samples from any desired distribution. They are extensively utilized in contemporary statistics, particularly in Bayesian analysis. In Bayesian inference, the posterior distribution is the primary emphasis; nevertheless, it frequently cannot be expressed in a closed form, as is the case here. Two prevalent MCMC methods utilized in Bayesian estimation include the Metropolis–Hastings algorithm, presented by Hastings [47], and the Gibbs sampler, proposed by Geman and Geman [48]. Both methodologies utilize marginal posterior distributions throughout the sampling procedure. In this approach, it is necessary to reformulate the joint posterior distribution of μ and λ as presented in Equation (19). It is provided by

$$\Pi \left(\mu ,\lambda |\underline{x}\right)\propto \mu^{a-1}\lambda^{\frac{m}{2}+c-1}e^{-(\mu b+\lambda d)}\prod_{i=1}^{m}\sqrt{\frac{1}{x_{i}^{3}}}e^{-B_{i}}A_{i}^{R_{i}}$$

When examining the posterior distribution in Equation (19), the complete conditional posterior distributions for the parameter μ and the parameter λ are derived utilizing the informative gamma priors, as delineated below.    

(32) $$\Pi \left(\mu |\lambda ,\underline{x}\right)\propto \mu^{a-1}\;e^{-\mu b}\prod_{i=1}^{m}\exp\left(-\frac{\lambda {\left(x_{i}-\mu \right)}^{2}}{2\mu^{2}x_{i}}\right)A_{i}^{R_{i}}$$

and

(33) $$\Pi \left(\lambda |\mu ,\underline{x}\right)\propto \lambda^{\frac{m}{2}+c-1}\;e^{-\lambda d}\prod_{i=1}^{m}\exp\left(-\frac{\lambda {\left(x_{i}-\mu \right)}^{2}}{2\mu^{2}x_{i}}\right)A_{i}^{R_{i}}$$

Due to the inability to derive the marginal distributions $\Pi (\mu |\lambda ,\underline{x})$ and $\Pi (\lambda |\mu ,\underline{x})$ in closed form, we employ the Metropolis–Hastings algorithm, which facilitates the estimation of Bayesian point estimates and credible intervals for unknown parameters μ and λ, in addition to the entropy measures *H* and $H_{\alpha }$. A cyclic implementation of the Metropolis–Hastings sampling process to acquire approximate samples from the posterior distribution Π. This can be acquired using the subsequent steps:

Step 1. Specify starting values $\mu^{\left(0\right)}$ and $\lambda^{\left(0\right)}$ (assume MLEs of μ and λ) and specify J=1.
Step 2. Generate $\mu^{*}$ and $\lambda^{*}$ from the normal proposal distributions $N\left(\mu^{\left(J-1\right)},\sigma_{\mu }^{2}\right)$ and $N\left(\lambda^{\left(J-1\right)},\sigma_{\lambda }^{2}\right)$, respectively, where $\left[\sigma_{\mu }^{2}\right]$ and $\left[\sigma_{\lambda }^{2}\right]$ are the variances in μ and λ, which can be estimated from the inverse of Fisher information matrix.
Step 3. Determine the following acceptance probabilities:
  $$r_{\mu }=\min\left[\frac{\Pi \;(\mu^{*}|\lambda^{\left(J-1\right)},\underline{x})}{\Pi \;(\mu^{\left(J-1\right)}|\lambda^{\left(J-1\right)},\underline{x})},1\right]\;\mathrm{and}\;r_{\lambda }=\min\left[\frac{\Pi \;(\lambda^{*}|\mu^{\left(J-1\right)},\underline{x})}{\Pi \;(\lambda^{\left(J-1\right)}|\mu^{\left(J-1\right)},\underline{x})},1\right].$$
Step 4. Generate samples $u_{1}$ and $u_{2}$ from uniform distribution U(0,1).
Step 5. If $u_{1}\leq r_{\mu }$ (acceptance probability), accept the proposal and specify $\mu^{\left(J\right)}=\mu^{*}$, otherwise $\mu^{\left(J\right)}=\mu^{\left(J-1\right)}$. Similarly, if $u_{2}\leq r_{\lambda }$, accept the proposal and specify $\lambda^{\left(J\right)}=\lambda^{*}$, otherwise $\lambda^{\left(J\right)}=\lambda^{\left(J-1\right)}$.
Step 6. Compute the Shannon entropy and Rényi entropy indicators from Equation (7) and Equation (8), respectively:
  (34) $$H^{\left(J\right)}=\frac{1}{2}\ln\frac{2\pi e}{\lambda^{\left(J\right)}}+\frac{3}{2}\ln\mu^{\left(J\right)}-\frac{3}{2}\;e^{2{\left(\frac{\lambda }{\mu }\right)}^{\left(J\right)}}E_{1}\left(\frac{2\lambda^{\left(J\right)}}{\mu^{\left(J\right)}}\right)$$
  and
  (35) $$\;H_{\alpha }^{\left(J\right)}=\frac{1}{1-\alpha }\left[\frac{\alpha }{2}\ln\left(\frac{\lambda^{\left(J\right)}}{2\pi }\right)+\frac{\alpha \lambda^{\left(J\right)}}{\mu^{\left(J\right)}}+\ln2+\left(1-\frac{3\alpha }{2}\right)\ln\mu^{\left(J\right)}+\ln K_{1-\frac{3\alpha }{2}}\left(\frac{\alpha \lambda^{\left(J\right)}}{\mu^{\left(J\right)}}\right)\right]$$
Step 7. Specify J=J+1
Step 8. Repeat Step 1. to Step 6., $N_{\mathrm{MC}}$ (the overall number of iterations) times to obtain MCMC samples. $\left(H^{\left(1\right)},H_{\alpha }^{\left(1\right)}\right)$, $\left(H^{\left(2\right)},H_{\alpha }^{\left(2\right)}\right)$, …, $\left(H^{\left(N_{\mathrm{MC}}\right)},H_{\alpha }^{\left(N_{\mathrm{MC}}\right)}\right)$ represent the generated samples obtained from the MH algorithm. After discarding the first *M* number of burn-in samples, the remaining $N_{\mathrm{MC}}-M$ samples are used to compute Bayesian estimates of *H* and $H_{\alpha }$. The Bayes estimate of $\eta =\left(H,H_{\alpha }\right)$ under the SE, GE, and LINEX loss functions can now be computed as:
  (36) $${\hat{\eta }}_{\mathrm{GE}}={\left[\frac{1}{N_{\mathrm{MC}}-M}\sum_{j=M+1}^{N_{\mathrm{MC}}}{\left(\eta^{j}\right)}^{-q}\right]}^{-\frac{1}{q}}$$
  and
  (37) $${\hat{\eta }}_{\mathrm{LINEX}}=-\frac{1}{p}\ln\left[\frac{1}{N_{\mathrm{MC}}-M}\sum_{j=M+1}^{N_{\mathrm{MC}}}e^{-p\eta^{j}}\right]$$
  The Bayes estimate of ${\hat{\eta }}_{\mathrm{SE}}$ with respect to the SE loss function can be derived from Equation (36) when q=−1.
Step 9. Order the values $\eta^{\left(M+1\right)},\eta^{\left(M+2\right)},\ldots ,\eta^{\left(N_{\mathrm{MC}}\right)}$ ascendingly as $\eta^{\left(1\right)}<\eta^{\left(2\right)}<\cdots <\eta^{\left(N_{\mathrm{MC}}-M\right)}$. Assuming the desired confidence level is $100(1-\tilde{p})\%$ (where $\tilde{p}$ is the significance level), the standard Bayesian credible interval for the parameter η is given by
  (38) $$\left[\eta_{\left(\lfloor \left(N_{\mathrm{MC}}-M\right)\;\cdot \;\left(\tilde{p}/2\right)\rfloor \right)}\;,\;\eta_{\left(\lfloor \left(N_{\mathrm{MC}}-M\right)\;\cdot \;\left(1-\tilde{p}/2\right)\rfloor \right)}\right]$$
  where ⌊.⌋ denotes the integer part (floor function). $\eta_{\left(k\right)}$ is the $k^{th}$ element in the ordered sequence. $\tilde{p}/2$ is the left-tail probability, and $1-\tilde{p}/2$ is the right-tail probability. Therefore, the HPD of η can be constructed.

With all three Bayesian computational methods now defined (Lindley’s approximation, importance sampling, and MCMC). This methodological summary is illustrated in Algorithm 1, their practical performance in estimating Shannon and Rényi entropies under progressive Type-II censoring is assessed through extensive simulations in the following section.
**Algorithm 1:** Bayesian estimation of *H(μ, λ)* and *H_α_(μ, λ)* for the IG distribution under progressive Type-II censoring
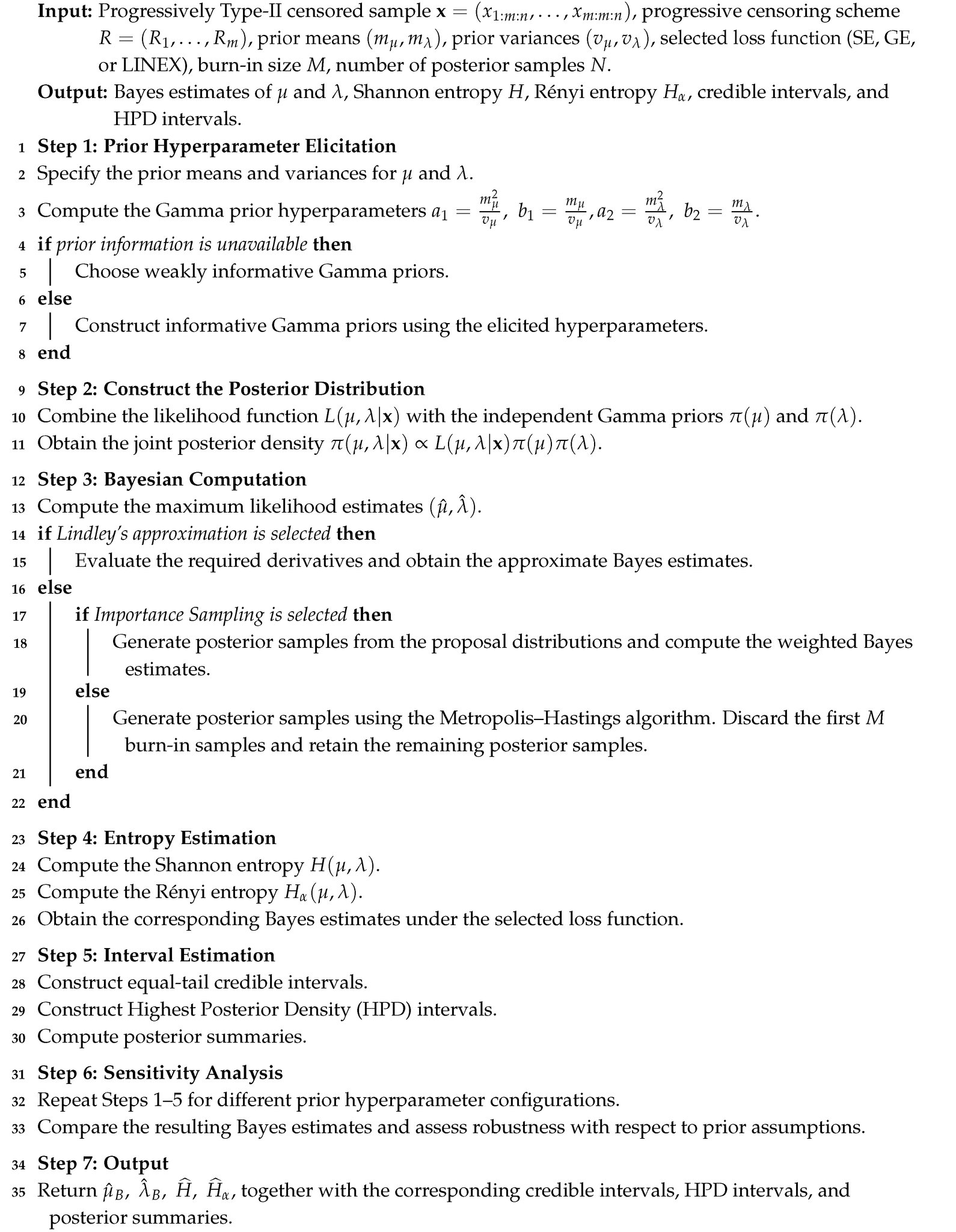



## 7. Simulation Study

A comprehensive simulation study was carried out to assess the performance of the proposed estimation methods under progressive Type-II censoring of the entropy functions given by Equations (7) and (8). The study was based on 1000 Monte Carlo replications. Let *n* be the total sample size and *m* (m≤n) be the effective sample size after censoring. Progressive Type-II censored schemes are defined by the removal vector $R=(R_{1},R_{2},\ldots ,R_{m})$, where $R_{i}$ units are randomly removed at the $i^{th}$ failure time. Three censoring schemes (S1, S2, and S3) were examined for different sample sizes (n=30,50,100) and effective sample sizes (m=20,40,60,75). The schemes are summarized in Table 2. Two parameter settings were considered to evaluate performance under different distributional shapes: Setting I with μ=1.5, λ=2.0, yielding true Shannon entropy H=1.2481 and Rényi entropy $H_{\alpha }=1.6894$ (at α=0.5); and Setting II with μ=2.0, λ=1.0, yielding true Shannon entropy H=1.5641 and Rényi entropy $H_{\alpha }=2.2997$ (at α=0.5). For the Bayesian procedures, the informative priors μ∼Gamma(3,2) and λ∼Gamma(10,5) were adopted, yielding prior means of 1.5 and 2, respectively, consistent with the parameter values of Setting I. Notably, these prior means do not match the parameter values of Setting II, thereby providing an implicit robustness check: the Bayesian procedures were tested under a prior specification centered away from the true values in Setting II. The results confirmed the stability of the MCMC and importance sampling methods across both settings, reflecting the dominance of the likelihood over the prior.

In each replication, samples were generated from an IG distribution with parameters (μ=1.5,λ=2) and (μ=2,λ=1) using the progressive Type-II censoring algorithm presented by Balakrishnan and Sandhu [49] as follows:

Step 1. Generate *m* independent Uniform (0,1) observations $w_{1}^{*},w_{2}^{*},\ldots ,w_{m}^{*}$.
Step 2. For a chosen censoring scheme from Table 2, set the removal vector $R_{i}$ for i=1,2,…,m.
Step 3. Set $E_{i}^{*}={\left(i+\sum_{j=m-i+1}^{m}R_{j}\right)}^{-1}$ for i=1,2,…,m.
Step 4. Set $V_{i}^{*}={\left(w_{i}^{*}\right)}^{E_{i}^{*}}$ for i=1,2,…,m.
Step 5. Set $U_{i,m,n}^{*}=U_{i}^{*}=1-\;\prod_{k=m-i+1}^{m}\;V_{k}^{*}$ for i=1,2,…,m. Then, $U_{1}^{*},U_{2}^{*},\ldots ,U_{m}^{*}$ is the required progressive Type-II censored sample from the Uniform(0,1) distribution.
Step 6. Finally, the progressive Type-II censored sample from the IG distribution is obtained by setting $X_{i}=F^{-1}\left(U_{i}^{*}\right)$, where $F^{-1}\left(\;\cdot \;\right)$ is the inverse CDF from Equation (4), which must be solved numerically.

For the MCMC method, we ran the parameter generation chain for 10,000 iterations, discarding the first 2000 as the burn-in period. For importance sampling, we generated 5000 parameter samples and then subjected them to the specified progressive censoring scheme. For each replication, point and interval estimates were calculated. Summary measures, such as mean estimates, bias, and coverage probabilities, are obtained by averaging over the 1000 Monte Carlo runs. The simulation results are summarized in Table 3, Table 4 and Table 5 as shown:

In Table 3, Pr. MLE exhibited poor performance, with a substantial positive bias (0.33–0.41) and a high mean relative error (0.30–0.34), showing only limited improvement as the sample size increased. In contrast, the IS, MCMC, and Lindley methods (under the GE and LINEX loss functions) produced highly accurate estimates, with low mean relative errors (0.07–0.14) and biases close to zero, whereas the Lindley estimator under the SE loss function was the least efficient among these superior methods. The inferior performance of the Pr. MLE can be attributed to the Shannon entropy formula, which contains the highly sensitive term $e^{2\lambda /\mu }E_{1}\left(2\lambda /\mu \right)$. This term amplifies parameter estimation errors arising from the complex likelihood surface of the IG distribution. By contrast, the Bayesian methods and IS overcome this limitation by relying on the entire posterior (or sampling) distribution of the entropy rather than on a single point estimate.

In Table 4, performance of the Pr. MLE improved considerably compared with the Shannon entropy results. It yielded a smaller negative bias −0.02 to −0.09 and an acceptable mean relative error (0.074–0.174), with clear improvement as the sample size increased. Nevertheless, the IS, MCMC, and Lindley methods remained more accurate, achieving MRE values ranging from 0.068 to 0.126. However, the differences among all estimation methods became less pronounced, and the performance of the different loss functions within the Lindley approach was remarkably similar. This improvement can be explained by the mathematical form of Rényi entropy, which depends on the modified Bessel function $K_{\nu }$. This function is smoother and less prone to magnifying estimation errors, while the parameter moderates the influence of variations in the ratio λ/μ, making the entropy less sensitive to parameter estimation errors.

In Table 5, Wald-CIs consistently produced excessive coverage (with the coverage probability often reaching 1.000), but at the expense of excessively wide and impractical interval lengths. For example, the interval length reached 4.33 for Shannon entropy when n=30 and remained approximately three to four times longer than those of the competing methods even when n=100. In contrast, the IS and MCMC approaches (using both equal-tail confidence intervals and HPD credible intervals) generated substantially shorter intervals (0.46–0.53 for Shannon entropy when n=100) while maintaining coverage probabilities close to the nominal 0.95 level. The excessive width of the Wald intervals reflects the instability of the covariance matrix caused by the irregular likelihood surface. In comparison, the simulation-based methods produce more efficient interval estimates because they are constructed from the full empirical distribution of the entropy, thereby capturing the dependence structure between the model parameters. It is also noteworthy that the interval lengths for Rényi entropy were consistently shorter than those for Shannon entropy across all methods, further confirming the greater numerical stability of the Rényi entropy formulation.

The simulation results are summarized in Table 6, Table 7 and Table 8 as shown:

In Table 6, Pr. MLE exhibited a positive bias ranging from 0.74 to 0.90 and a mean relative error between 0.49 and 0.58, both of which were higher than those observed under the first set of simulation settings. The Lindley method showed some variability in performance, with relatively large mean squared error values at small sample sizes, particularly under Scenario S1, although its accuracy improved noticeably as the sample size increased. In contrast, the IS and MCMC methods produced more accurate estimates, with mean relative errors ranging from 0.067 to 0.12 and biases closer to zero, while maintaining stable performance across all simulation scenarios.

In Table 7, Pr. MLE performed relatively better than it did for Shannon entropy, yielding a negative bias ranging from −0.015 to −0.126 and a mean relative error between 0.077 and 0.207. The stability issues observed for the Lindley method were limited to the SE loss function at small sample sizes, whereas the performance of the different loss functions became increasingly similar as the sample size grew. The IS and MCMC methods maintained consistently stable performance, with mean relative errors ranging from 0.068 to 0.113 and no notable outlying values.

In Table 8, for Shannon entropy, the Wald-CIs were relatively wide, and their coverage probability declined as the sample size increased, reaching as low as 0.500 in some scenarios. In contrast, the Wald-CIs for Rényi entropy maintained relatively high coverage probabilities but remained wider than the corresponding intervals produced by the competing methods. The IS and MCMC interval estimators achieved coverage probabilities close to the nominal level in most cases while producing considerably shorter intervals than the Wald-CIs, although slight reductions in coverage were observed for certain sample sizes. Overall, the interval estimation results for Rényi entropy were more stable than those for Shannon entropy across all estimation methods.

**Figure 2:**
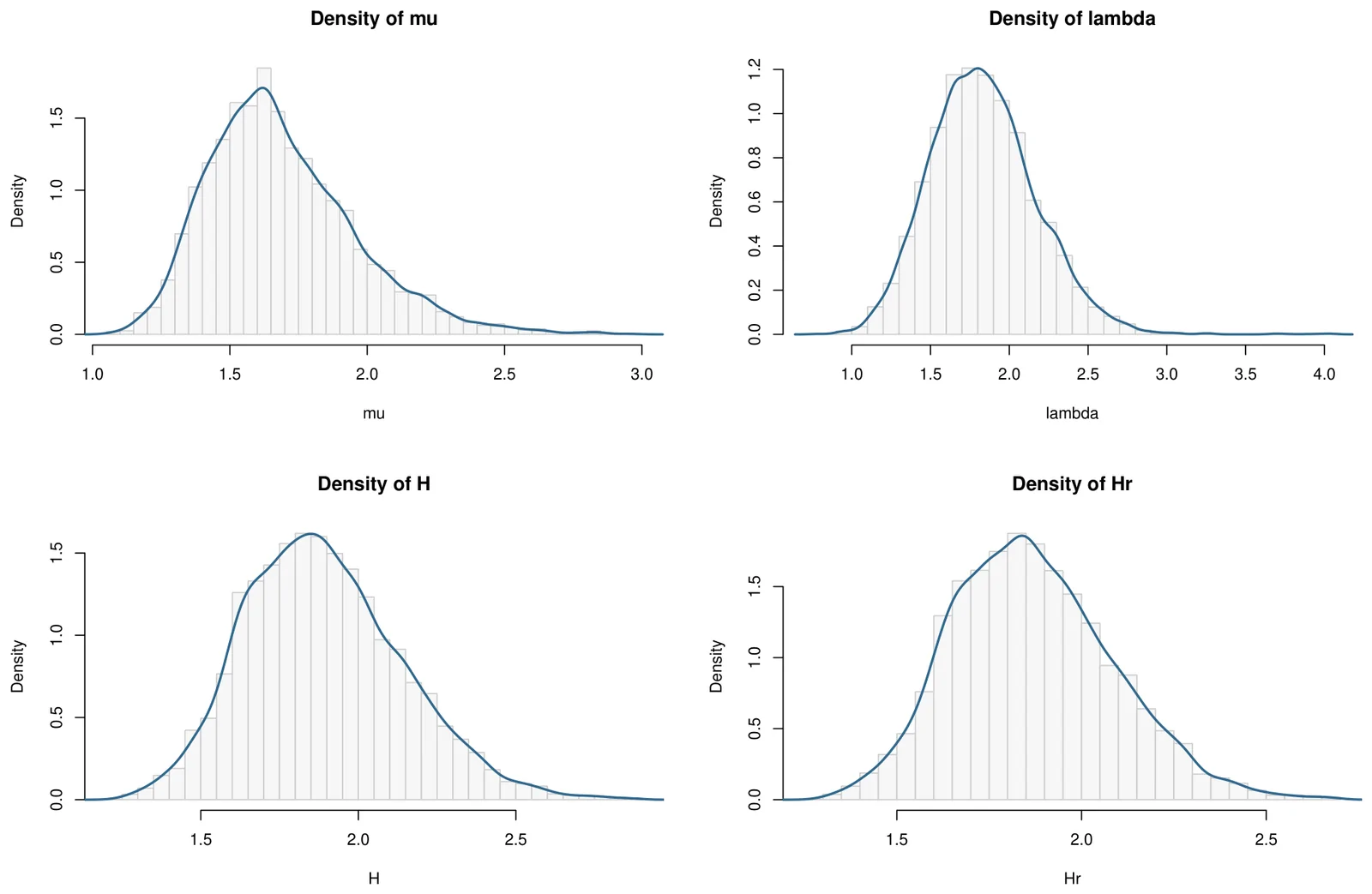
IG MCMC diagnostics density.

**Figure 3:**
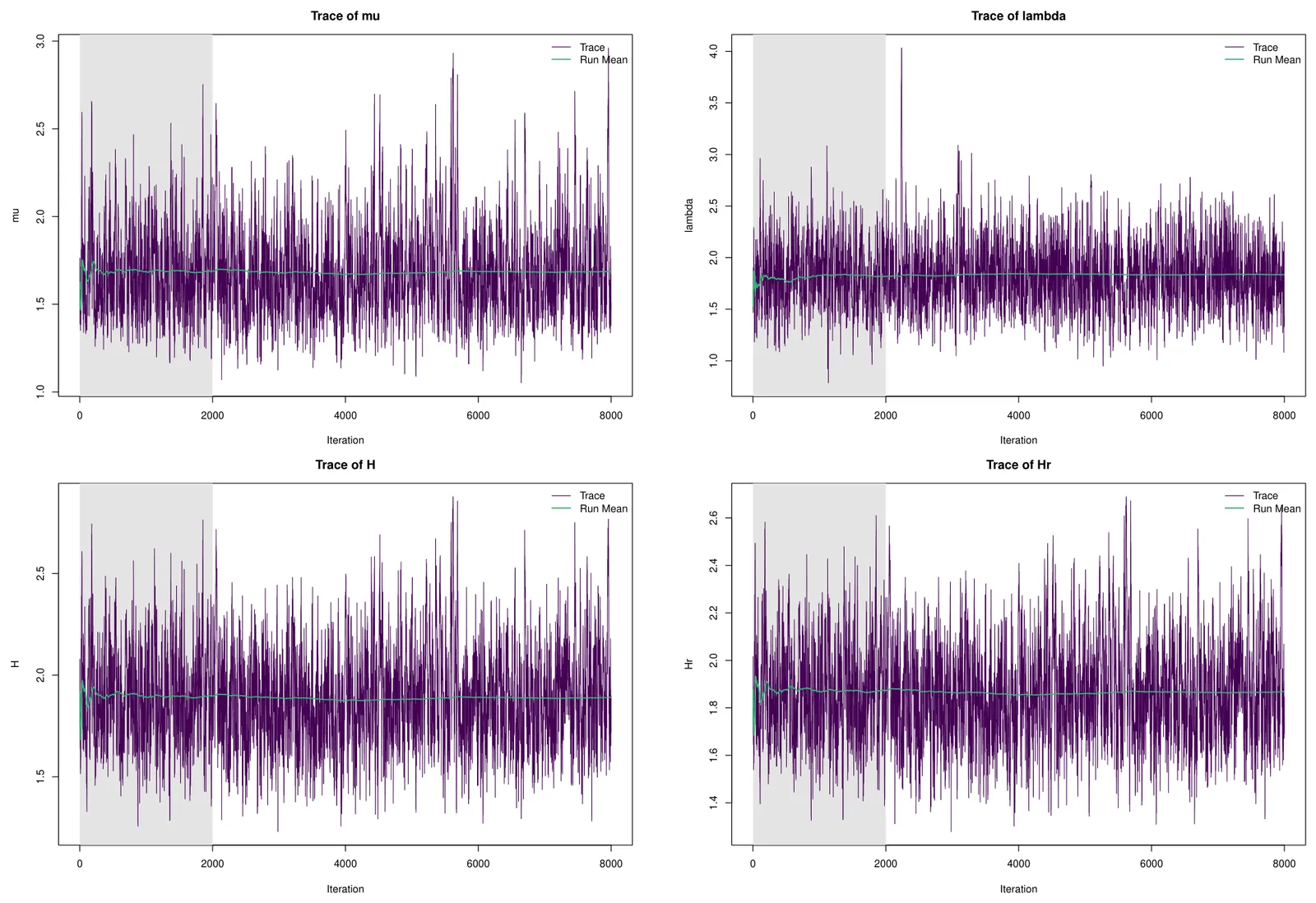
IG MCMC diagnostics grid.

**Figure 4:**
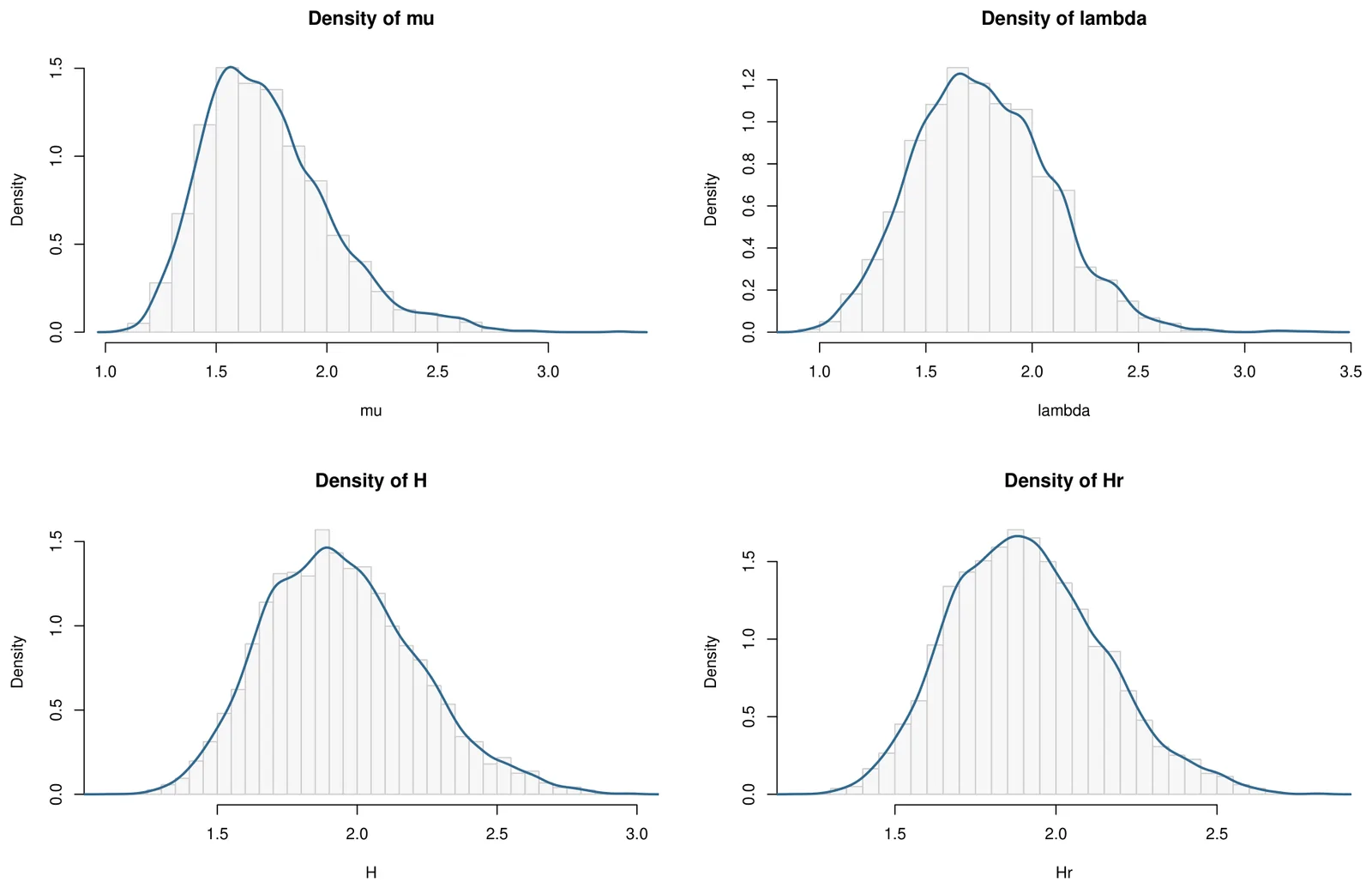
IG MCMC diagnostics density n50 m40 R1.

**Figure 5:**
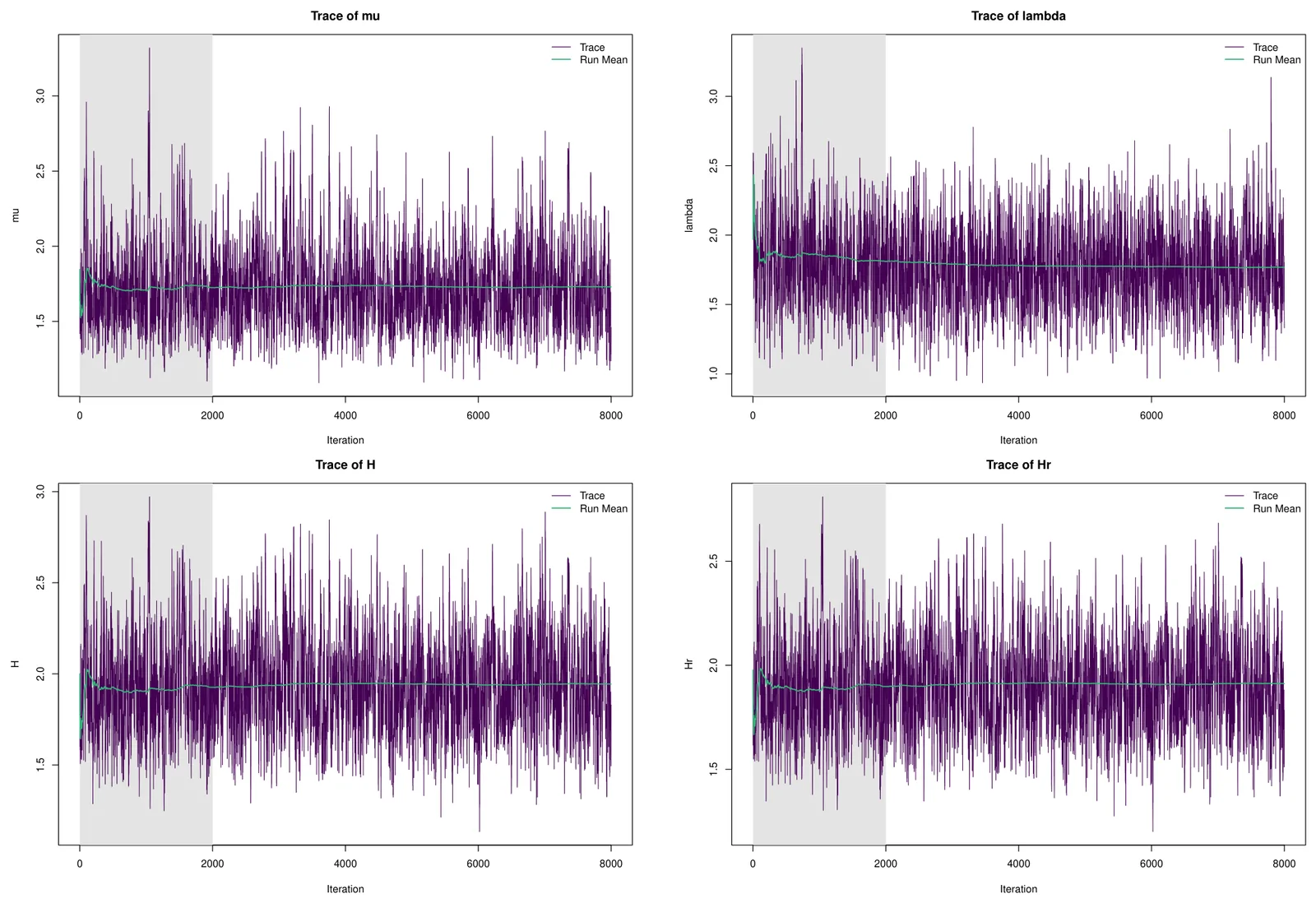
IG MCMC diagnostics grid n50 m40 R1.

The results of Figure 2, Figure 3, Figure 4 and Figure 5 show the effectiveness of the MCMC approach for a variety of parameters and entropy measures settings. This suggests that the MCMC approach is a powerful and reliable method to sample complex distributions.

## 8. Real Data

Across both Dataset I and Dataset II, the IG distribution consistently demonstrated superior performance compared to the following distributions based on both the AIC criterion and the Kolmogorov–Smirnov goodness-of-fit test results; Epanechnikov–Weibull (EpW) distribution; Perks (Perk) distribution; Gompertz–Lindley (GomLin) distribution; Gamma (Gam) distribution; Power Muth (PMuth) distribution; Chen distribution; Type-II Heavy-Tailed Weibull (T2HTW) distribution; Generalized Exponential (GExp) distribution; Power Lindley (PLin) and Weibull (W) distribution; and Power Rayleigh (PRay) distribution.

### 8.1. Data Set I

The dataset consists of the follow-up times (in months) for 35 children undergoing growth hormone therapy, measured from the initiation of treatment until reaching the targeted developmental age: 2.15, 2.20, 2.55, 2.56, 2.63, 2.74, 2.81, 2.90, 3.05, 3.41, 3.43, 3.43, 3.84, 4.16, 4.18, 4.36, 4.42, 4.51, 4.60, 4.61, 4.75, 5.03, 5.10, 5.44, 5.90, 5.96, 6.77, 7.82, 8.00, 8.16, 8.21, 8.72, 10.40, 13.20, 13.70. Data were obtained from the Minas Gerais Health Secretariat Hormonal Program, Brazil, and were previously used in a published study to apply new statistical distributions in biomedical and epidemiological contexts [50].

Table 9 presents parameter and entropy estimates under different removal scenarios. With complete data, the results are automatically identical. It is observed that removal from the beginning or middle of the data affects the estimates to a greater extent than removal from the end, particularly at higher deletion rates, as reflected in the variation in Shannon entropy values and the widening of confidence intervals for the parameter λ. These results suggest that the impact of data removal depends not only on the deletion rate but also on the position of the removed values, which should be taken into account when dealing with incomplete data.

Table 10 shows that the IG distribution achieves the lowest information criterion values (AIC = 153.96, BIC = 157.53) and the highest *p*-values for the goodness-of-fit tests (AD *p* = 0.987, KS *p* = 0.979), indicating a better fit to the data. It is followed by GomLin, while the Chen and PMuth distributions show the weakest performance. The information criteria and goodness-of-fit tests agree on the ranking of distributions without contradiction, with a clear gap between IG and its closest competitor.

**Figure 6:**
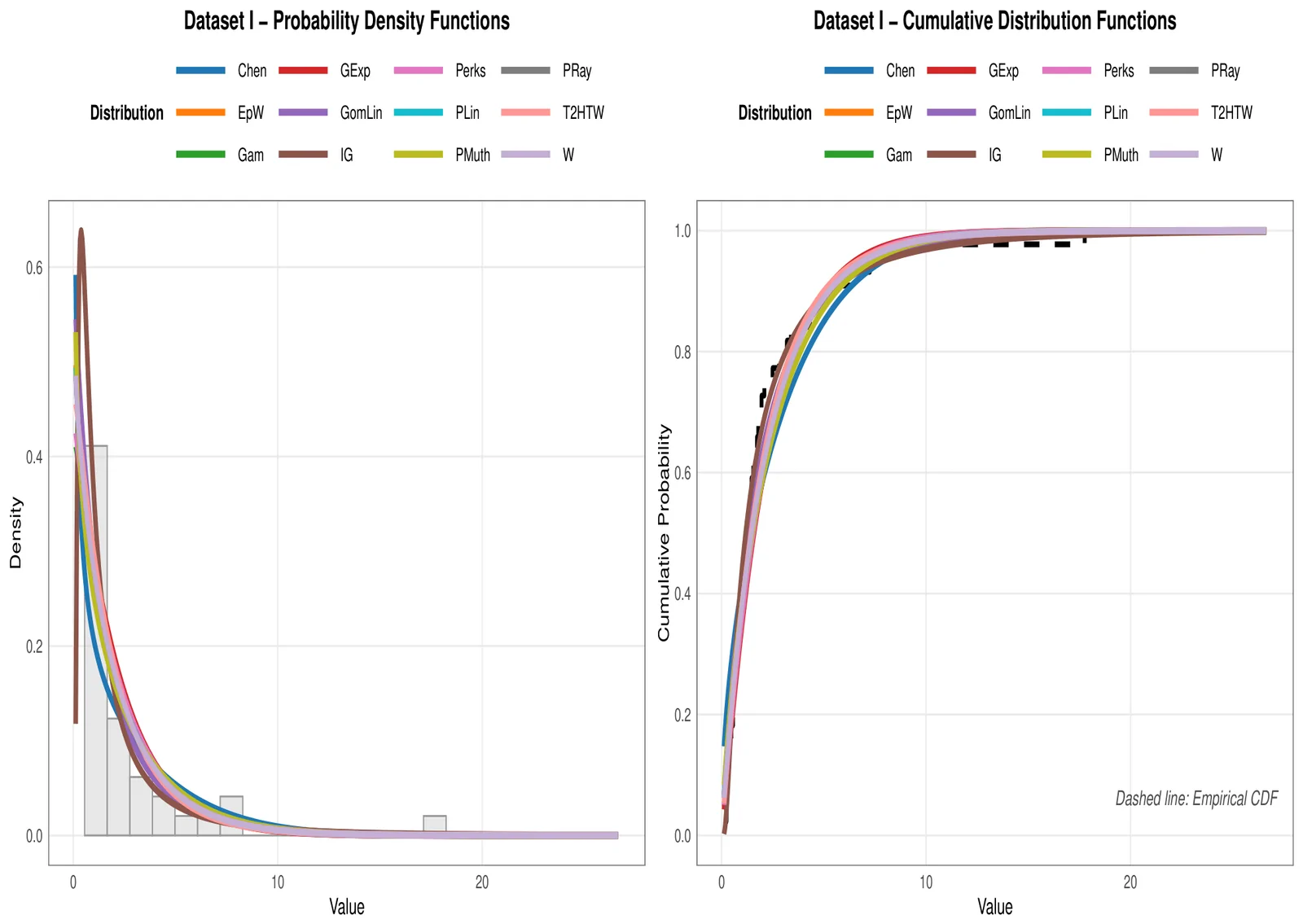
Fitted probability distributions for Dataset I.

Figure 6 provides a comprehensive comparison of all distributions in terms of probability density and cumulative distribution functions. Most curves converge around the lower values, but differences appear in the upper tail of the data, where the IG distribution maintains a better representation of the general behavior compared to distributions that deviate from the empirical histogram.

**Figure 7:**
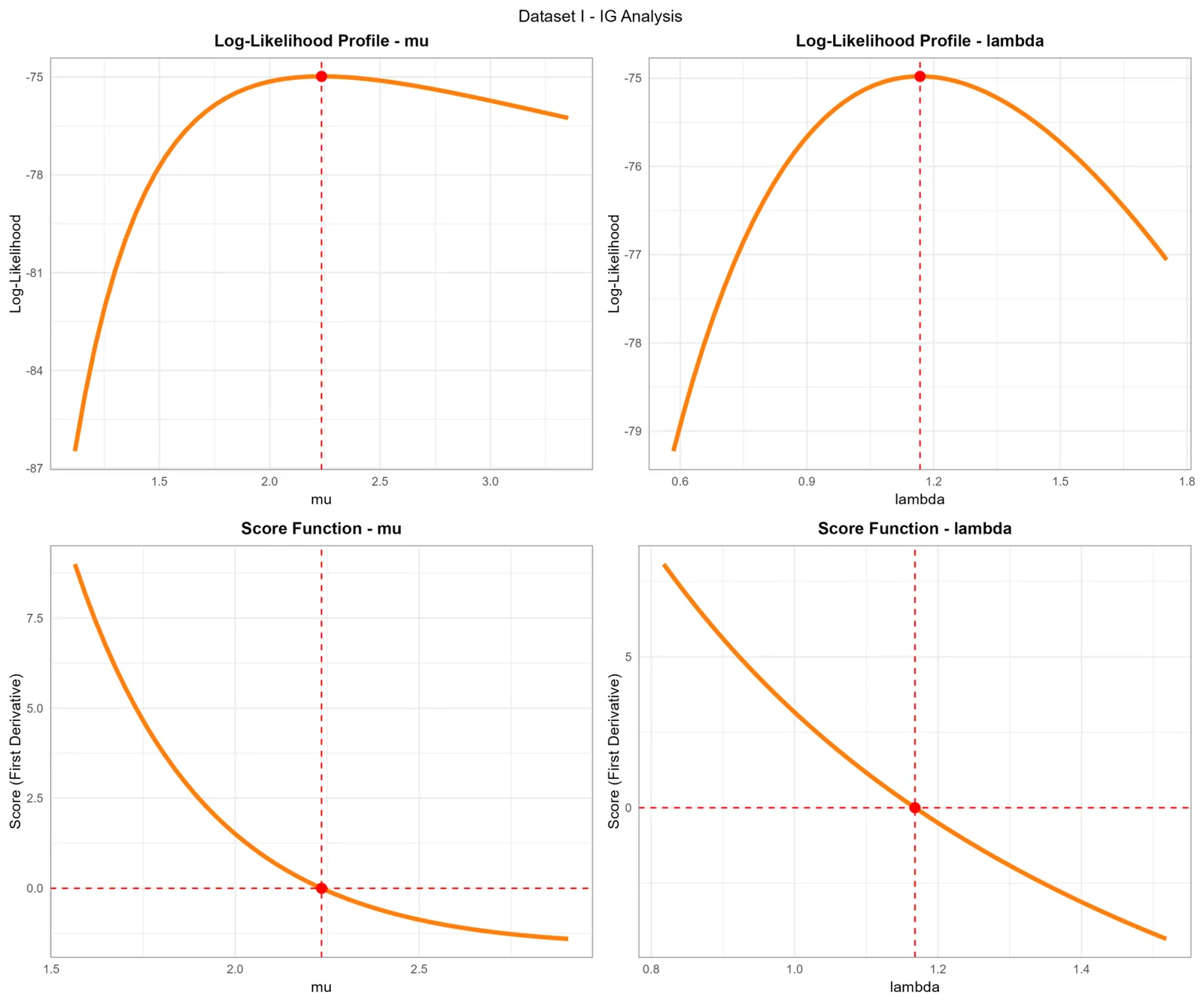
Inverse Gaussian distribution diagnostics for Dataset I: log-likelihood profiles and score functions for both parameters.

Figure 7 displays the parameter estimation for the IG distribution via MLE. The top plots show a convex logarithmic likelihood profile, while the bottom plots present the score function intersecting zero, confirming that the values chosen for the parameters μ and λ are the optimal estimates.

**Figure 8:**
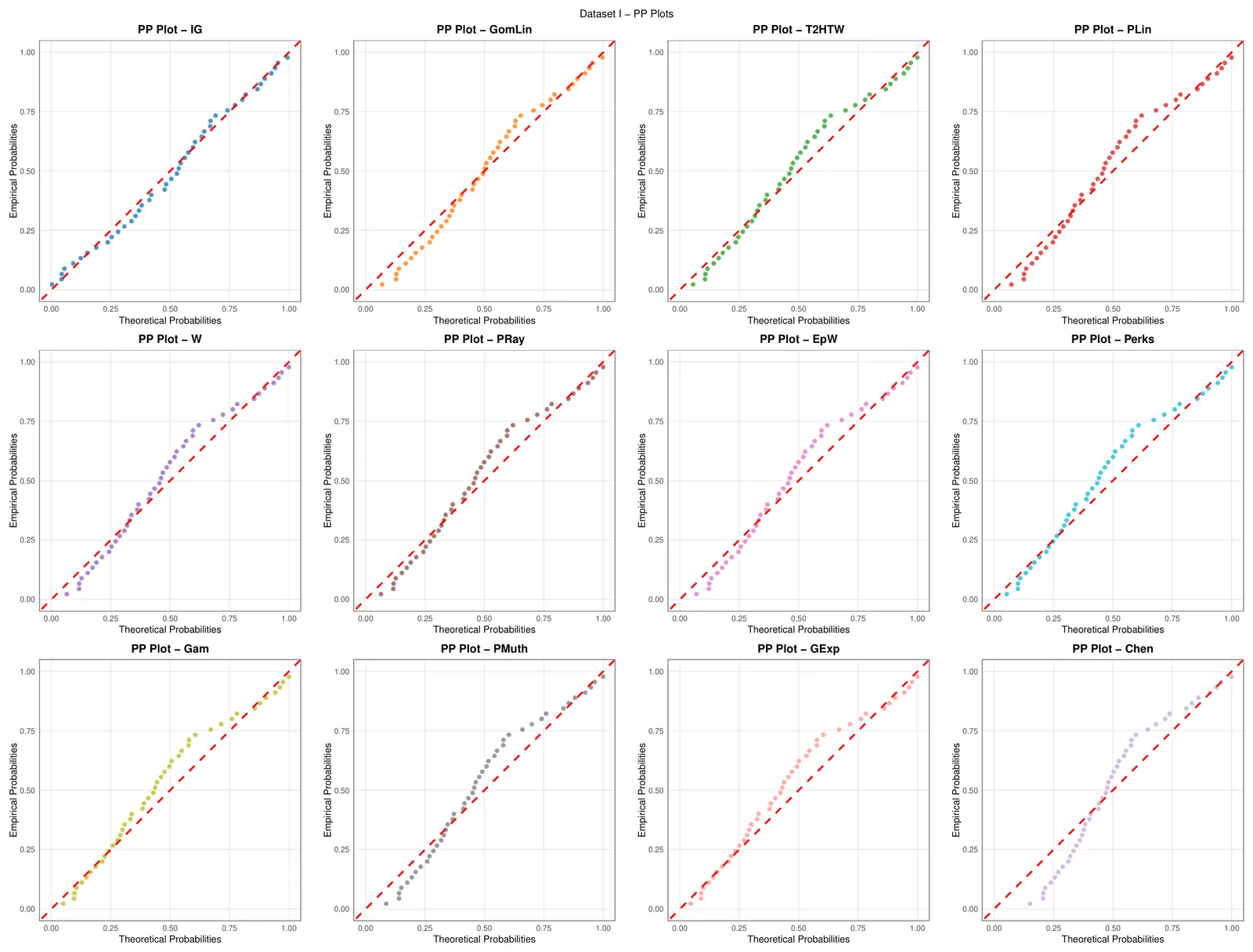
Probability– Probability (PP) plots for Dataset I under fitted candidate models.

Figure 8 compares empirical probabilities against theoretical probabilities for 12 distributions. The IG distribution exhibits close alignment with the red diagonal line, whereas other distributions, such as Chen and Plin, show noticeable deviation. These results indicate that the IG distribution provides a better fit for the data compared to the other models.

**Figure 9:**
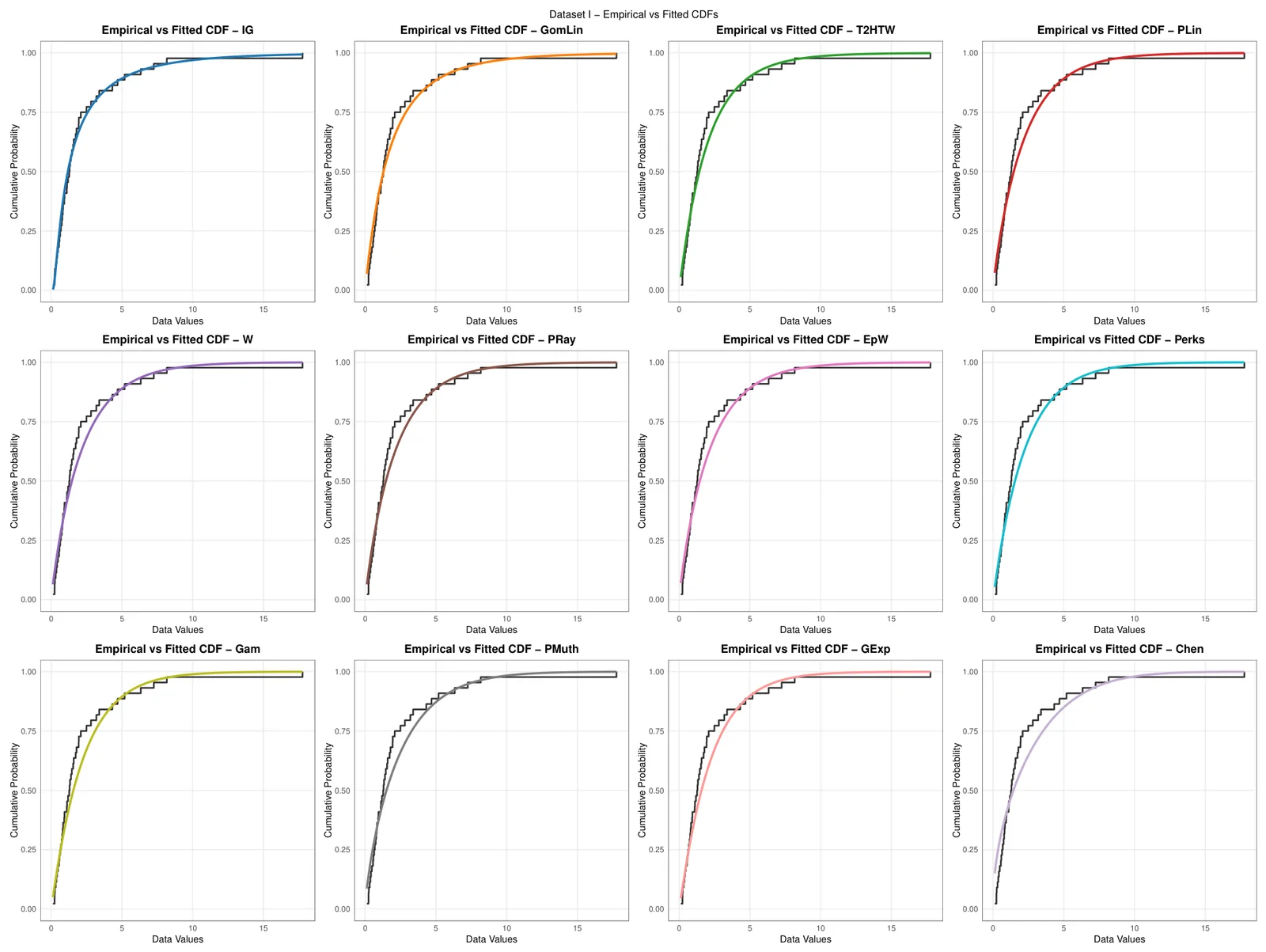
Empirical versus fitted CDF plots for Dataset I under fitted candidate models.

Figure 9 compares the empirical cumulative distribution function (black stepped line) with the theoretical functions of various distributions. Good agreement is observed for the IG and GomLin distributions with the empirical data, while other distributions show deviation in the tail regions, supporting the quality of the IG distribution in representing the cumulative structure of the data.

### 8.2. Data Set II

This dataset represents the survival times (in days) of 44 patients with Head and Neck Cancer (HNC) who underwent a combined radiation therapy and chemotherapy treatment regimen. 0.1220, 0.2356, 0.2374, 0.2587, 0.3198, 0.3700, 0.4135, 0.4738, 0.5546, 0.5836, 0.6347, 0.6846, 0.7447, 0.7826, 0.8143, 0.8400, 0.9200, 0.9400, 1.1000, 1.1200, 1.1900, 1.2700, 1.3000, 1.3300, 1.4000, 1.4600, 1.5500, 1.5900, 1.7300, 1.7900, 1.9400, 1.9500, 2.0900, 2.4900, 2.8100, 3.1900, 3.3900, 4.3200, 4.6900, 5.1900, 6.3300, 7.2500, 8.1700, 17.7600, showing a classic right-skewed distribution common in survival analysis, where most events occur early but a long tail extends to the right. Such data are typically modeled using heavy-tailed probability distributions (like the ZLindley or generalized Weibull models mentioned in the titles) to accurately capture the risk of mortality over time and to assess the efficacy of the simultaneous treatment protocol.

Table 11 presents parameter and entropy estimates under different removal scenarios. With complete data, the results are automatically identical. It is evident that removal from the middle (S2) and beginning (S3) affects estimates to a greater extent than removal from the end (S1), particularly at higher deletion rates, as reflected in the variation in Shannon entropy values and the widening of confidence intervals for the parameter μ. This pattern is consistent with what was observed for the growth hormone data, confirming that the impact of data removal depends on the position of the removed values and not only on the deletion rate. This should be taken into account when dealing with incomplete data.

Table 12 summarizes the comparative performance of the candidate probability distributions fitted to Dataset II. Among the competing models, the IG distribution consistently provides the best fit, as evidenced by the smallest information criteria (AIC = 160.38 and BIC = 163.49) and the largest goodness-of-fit *p*-values (AD *p* = 0.87, CvM *p* = 0.84, and KS *p* = 0.93). These findings identify the IG distribution as the most suitable model for Dataset II. The GExp and Gam distributions rank next in terms of model adequacy, whereas the Chen and PMuth distributions provide comparatively poorer fits. All criteria and tests agree on the ranking of distributions without contradiction, reinforcing confidence in the adoption of IG for modeling.

**Figure 10:**
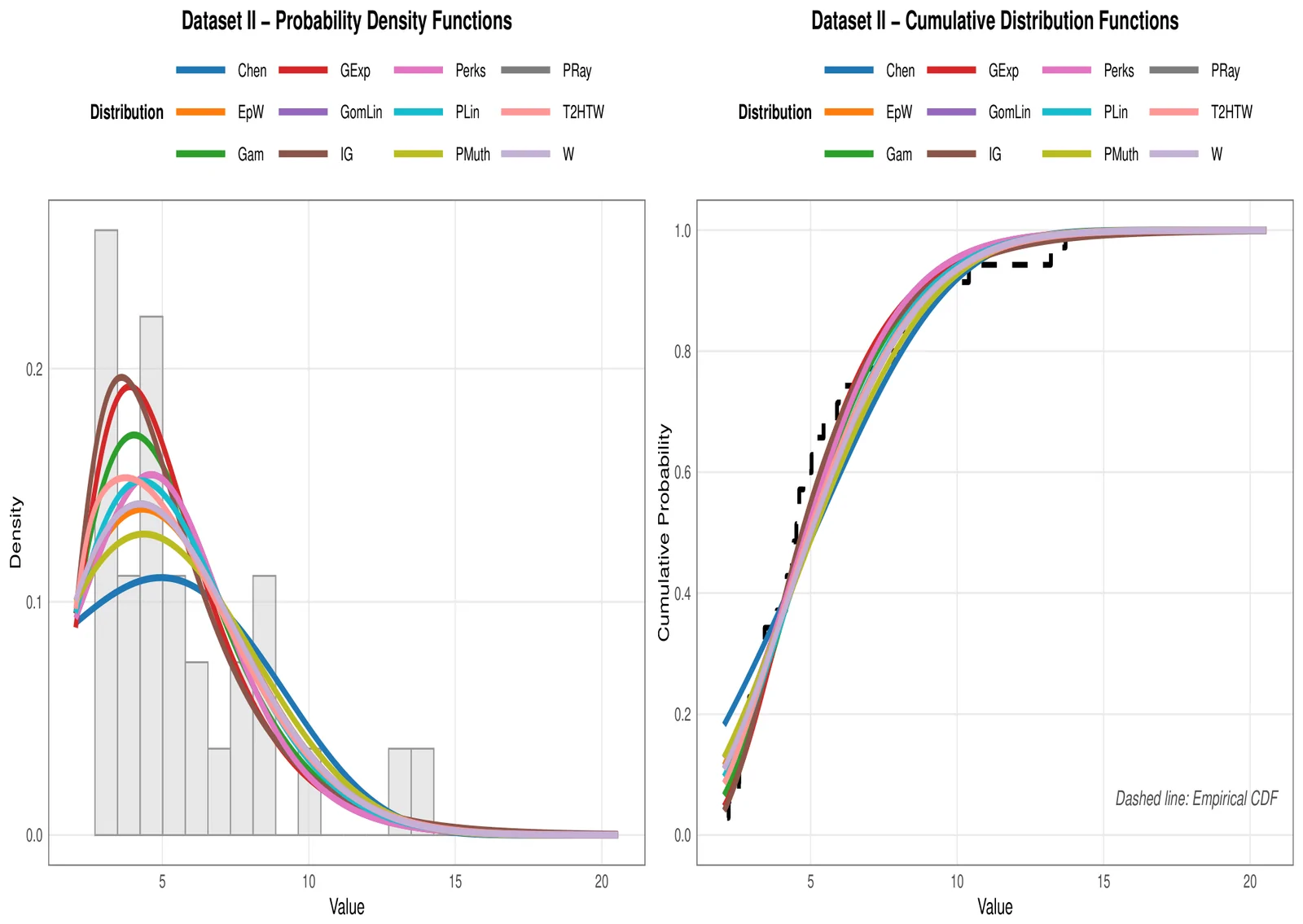
Fitted probability distributions for Dataset II.

**Figure 11:**
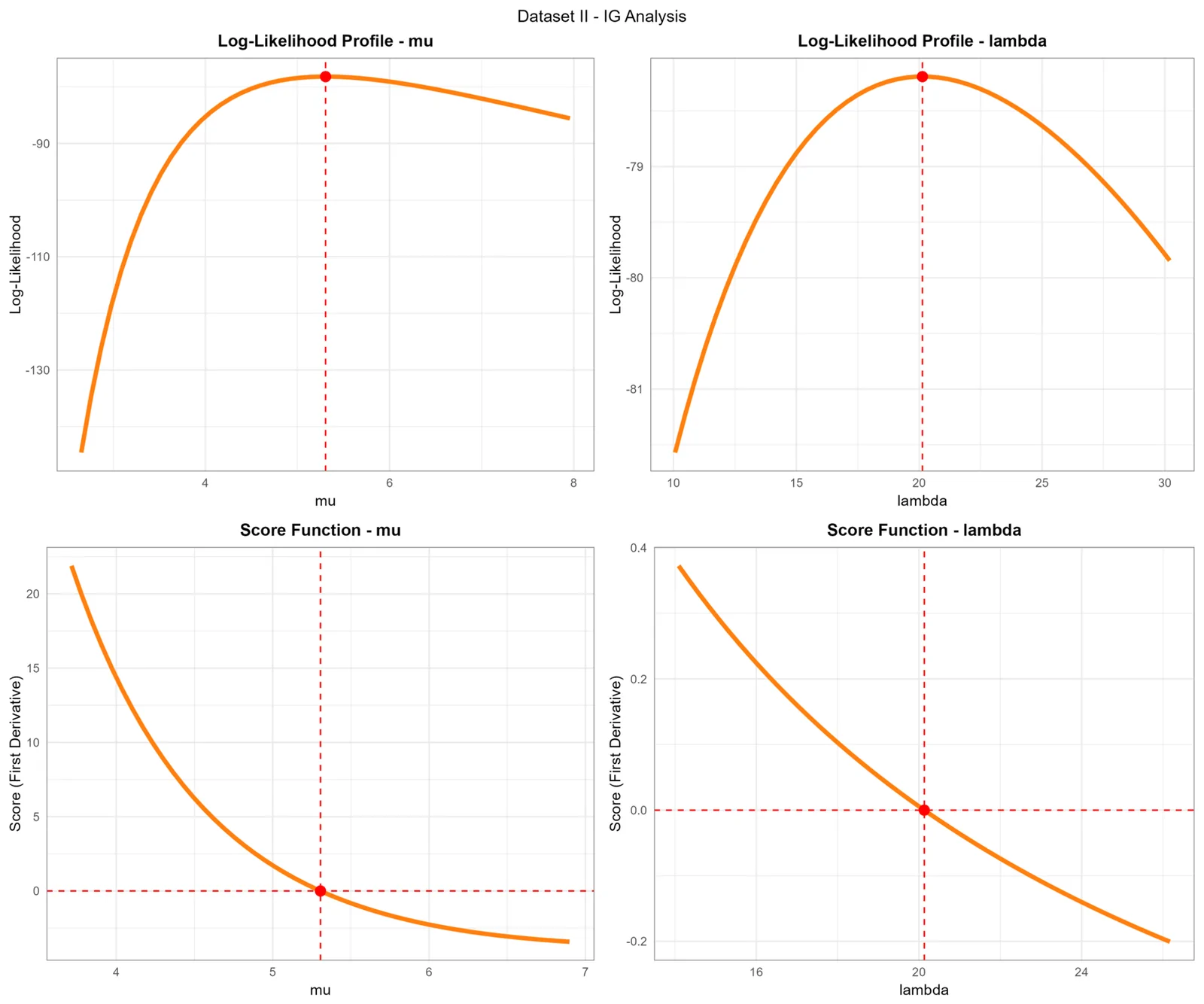
Inverse Gaussian distribution diagnostics for Dataset II: log-likelihood profiles and score functions for both parameters.

**Figure 12:**
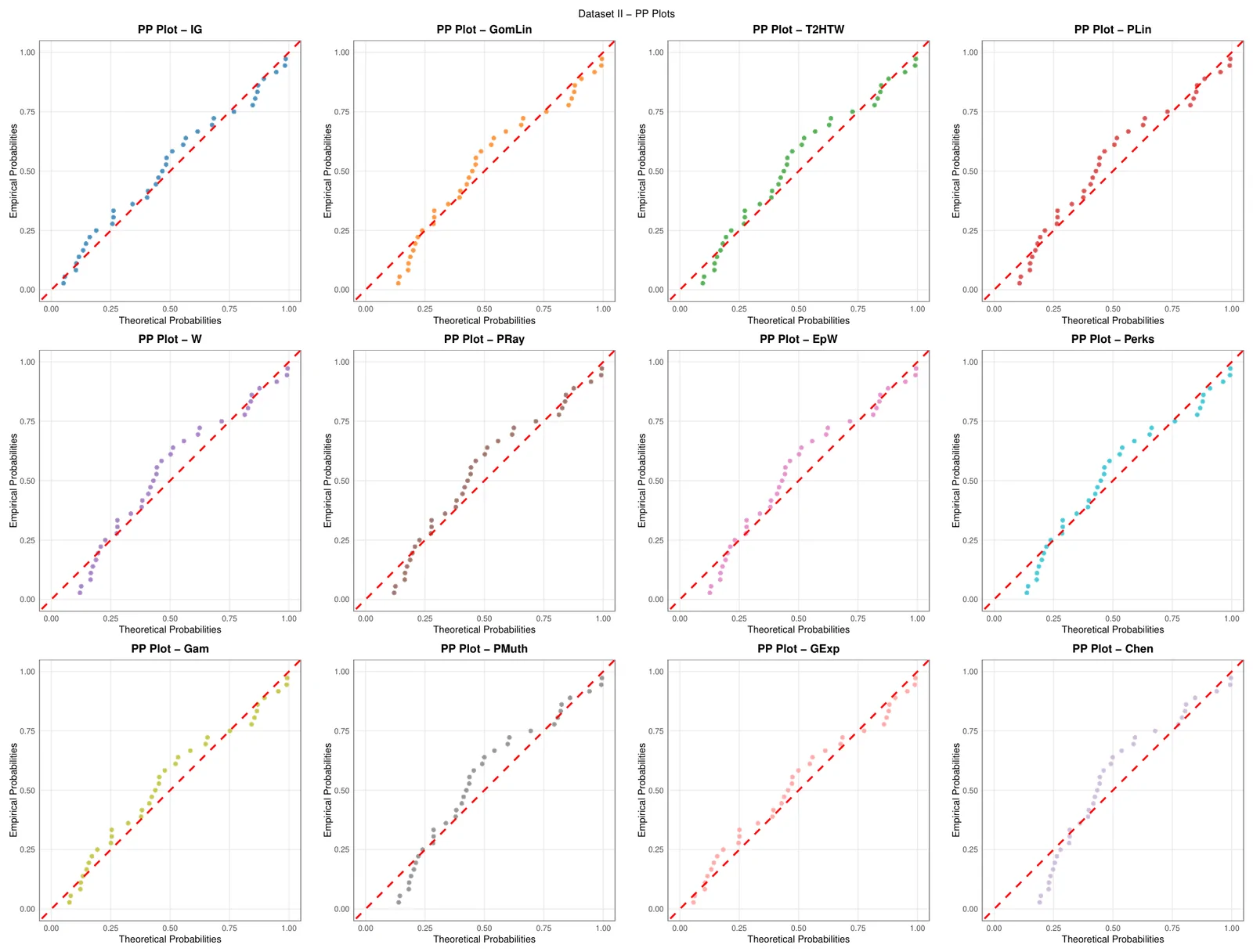
Probability–Probability (PP) plots for Dataset II under fitted candidate models.

**Figure 13:**
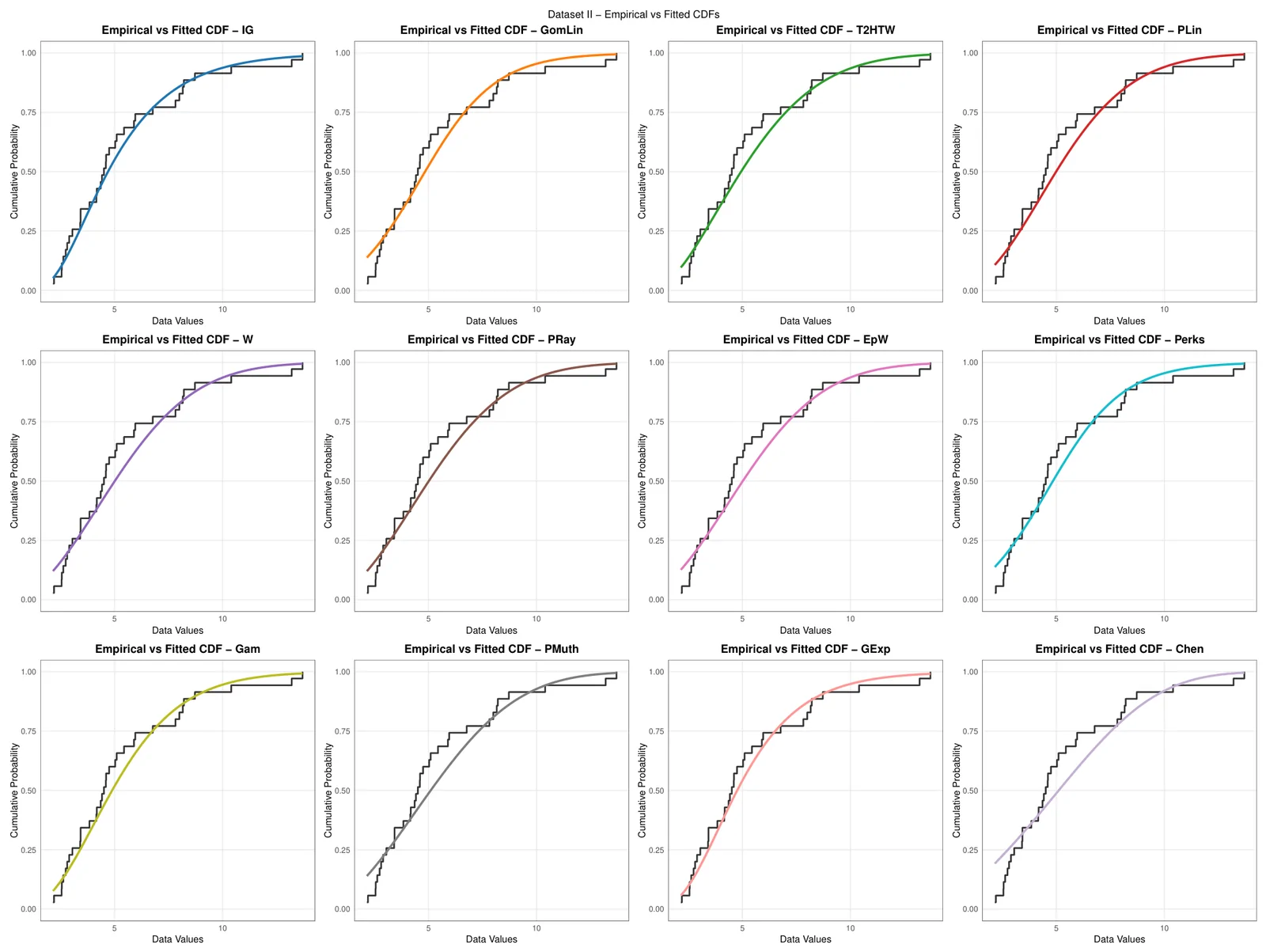
Empirical versus fitted CDF plots for Dataset II under fitted candidate models.

To avoid digression, Figure 10, Figure 11, Figure 12 and Figure 13 for dataset II also demonstrate the clear superiority of the Gauss inverse, as shown in Table 12 where results of dataset II confirm the pattern observed in dataset I, where the IG distribution is the best fit. All graphical displays (PP, CDF, Likelihood) showed consistent performance, in contrast to the clear poor fit of the other distributions.

## 9. Discussion

The simulation results, under the conditions considered in this study, showed that the MCMC and IS methods outperformed the MLE and Lindley methods in estimating both Shannon and Rényi entropies of the IG distribution. They achieved lower bias, smaller mean relative error, and more accurate confidence intervals. The performance of the MLE and its associated Wald confidence intervals deteriorated when the parameter values changed, particularly for Shannon entropy, whereas the MCMC and IS methods maintained relatively stable performance. The results also indicated, within the scope of this study, that Rényi entropy was less sensitive to changes in the parameter values than Shannon entropy, and that the GE and LINEX loss functions were more efficient than the SE loss function. Furthermore, the sensitivity analysis confirmed, for the settings examined, the robustness of the Bayesian estimates across the prior distributions considered.

## 10. Conclusions

The significance of this study is twofold:

- Scientifically, it provides the first comprehensive systematic comparison of four estimation methods for Shannon and Rényi entropies of the IG distribution under progressive censoring, a topic largely unexplored in the literature. The results reveal that estimator stability depends fundamentally on the mathematical form of the entropy, with the Rényi entropy showing to be substantially less sensitive to parameter variation, while the Shannon entropy deteriorated markedly when the λ/μ ratio changed. The study further documents the breakdown of maximum likelihood estimation and Wald confidence intervals under complex scenarios, as well as the numerical instability of Lindley’s approximation at small sample sizes.
- Practically, the study offers clear guidance in recommending MCMC and importance sampling as the optimal methods, with the general entropy and LINEX loss functions demonstrating superior performance. The stability of Bayesian estimates across different prior specifications further provides practitioners with flexibility even in the absence of precise prior information.

## 11. Future Research Directions

The findings of this study open several promising research avenues. First, the proposed methodological framework can be extended to other distributions with similar characteristics, such as generalized gamma distributions, to explore the performance of the different methods across broader distributional families. Second, extending the simulation framework to randomly distributed removal schemes, rather than single-stage withdrawals, would reveal whether our findings generalize to more realistic censoring scenarios.

## Figures and Tables

**Table 1:** Bayesian estimators under different loss functions.

Loss Function	Bayes Estimator
$\ell_{SE}\left(H,\hat{H}\right)={\left(\hat{H}-H\right)}^{2}$	${\hat{H}}_{B.SE}=E\left(H|\underline{x}\right)$
$\ell_{GE}\left(H,\hat{H}\right)={\left(\frac{\hat{H}}{H}\right)}^{q}-q\ln\left(\frac{\hat{H}}{H}\right)-1,\;q\neq 0$	${\hat{H}}_{B.GE}={\left[E\left(H^{-q}|\underline{x}\right)\right]}^{-\frac{1}{q}}$
$\ell_{LINEX}\left(H,\hat{H}\right)=e^{p\;(\hat{H}-H)}-p\;\left(\hat{H}-H\right)-1,\;p\neq 0$	${\hat{H}}_{B.LINEX}=-\frac{1}{p}\ln\left[E\left(e^{-Hp}|\underline{x}\right)\right]$

q>0, a positive error is more dangerous than a negative error. q<0, a negative error is more dangerous than a positive error.

**Table 2:** Progressive censoring schemes considered in the simulation study.

Scheme	Removal Vector R	Description
S1	$R_{I}=\left(0,0,\ldots ,0,n-m\right)$	$R_{i}=0$ for i=1,…,m−1, and $R_{m}=n-m$. All removals occur after the last observed failure.
S2	$R_{\mathrm{II}}=\left(0,\ldots ,0,n-m,0,\ldots ,0\right)$	$R_{i}=0$ for i≠⌈m/2⌉, and $R_{\left\lceil m/2\right\rceil }=n-m$. All removals occur at the middle failure time.
S3	$R_{\mathrm{III}}=\left(n-m,0,0,\ldots ,0\right)$	$R_{1}=n-m$, and $R_{i}=0$ for i=2,…,m. All removals occur after the first observed failure.

For all schemes, the condition $\sum_{i=1}^{m}R_{i}=n-m$ is satisfied.

**Table 3:** Mean, Bias, MSE, RMSE, and MRE of ML and Bayes estimates for Shannon entropy at Setting I (μ=1.5, λ=2).

*n*	*m*	Sc.	Metric	Pr. MLE	Lindley			IS			MCMC		
					SE	GE	LINEX	SE	GE	LINEX	SE	GE	LINEX
30	20	S1	Mean	1.5850	1.8794	2.0081	1.9272	1.2650	1.2152	1.2355	1.2645	1.2144	1.2350
			Bias	0.3369	0.6313	0.7599	0.6790	0.0169	−0.0329	−0.0126	0.0163	−0.0337	−0.0132
			MSE	0.2829	0.4684	1.2493	0.7253	0.0408	0.0451	0.0405	0.0406	0.0454	0.0403
			RMSE	0.5319	0.6844	1.1177	0.8517	0.2019	0.2124	0.2012	0.2014	0.2130	0.2008
			MRE	0.3379	0.5083	0.6138	0.5472	0.1293	0.1362	0.1293	0.1289	0.1358	0.1288
30	20	S2	Mean	1.5831	1.8128	1.8278	1.8053	1.2579	1.2139	1.2319	1.2607	1.2163	1.2343
			Bias	0.3349	0.5647	0.5797	0.5572	0.0098	−0.0342	−0.0163	0.0125	−0.0318	−0.0139
			MSE	0.2600	0.3994	0.4610	0.3979	0.0438	0.0483	0.0439	0.0439	0.0482	0.0440
			RMSE	0.5099	0.6320	0.6790	0.6308	0.2093	0.2198	0.2096	0.2095	0.2196	0.2097
			MRE	0.3263	0.4550	0.4696	0.4500	0.1349	0.1418	0.1352	0.1351	0.1416	0.1352
30	20	S3	Mean	1.6013	1.7779	1.7513	1.7516	1.2542	1.2135	1.2303	1.2558	1.2147	1.2315
			Bias	0.3532	0.5297	0.5032	0.5034	0.0061	−0.0346	−0.0178	0.0076	−0.0335	−0.0166
			MSE	0.2370	0.3629	0.3489	0.3385	0.0443	0.0490	0.0445	0.0444	0.0489	0.0445
			RMSE	0.4868	0.6024	0.5906	0.5818	0.2104	0.2214	0.2110	0.2106	0.2212	0.2110
			MRE	0.3274	0.4325	0.4166	0.4135	0.1323	0.1380	0.1324	0.1330	0.1380	0.1324
50	40	S1	Mean	1.6466	1.7580	1.7238	1.7291	1.2713	1.2465	1.2558	1.2701	1.2458	1.2549
			Bias	0.3985	0.5099	0.4757	0.4809	0.0231	−0.0016	0.0077	0.0220	−0.0024	0.0068
			MSE	0.2212	0.3017	0.2649	0.2687	0.0212	0.0212	0.0206	0.0211	0.0211	0.0205
			RMSE	0.4703	0.5493	0.5147	0.5183	0.1458	0.1456	0.1434	0.1453	0.1453	0.1431
			MRE	0.3276	0.4085	0.3811	0.3853	0.0949	0.0940	0.0927	0.0949	0.0942	0.0930
50	40	S2	Mean	1.6587	1.7464	1.7145	1.7199	1.2696	1.2475	1.2558	1.2693	1.2475	1.2557
			Bias	0.4106	0.4983	0.4664	0.4718	0.0214	−0.0006	0.0077	0.0212	−0.0006	0.0076
			MSE	0.2198	0.2875	0.2556	0.2593	0.0202	0.0203	0.0197	0.0205	0.0205	0.0200
			RMSE	0.4688	0.5362	0.5056	0.5093	0.1422	0.1424	0.1404	0.1433	0.1433	0.1414
			MRE	0.3306	0.3992	0.3737	0.3780	0.0902	0.0908	0.0893	0.0914	0.0916	0.0903
50	40	S3	Mean	1.6418	1.7233	1.6951	1.7000	1.2562	1.2354	1.2434	1.2557	1.2351	1.2430
			Bias	0.3937	0.4752	0.4470	0.4519	0.0081	−0.0127	−0.0047	0.0076	−0.0131	−0.0051
			MSE	0.2113	0.2699	0.2427	0.2459	0.0231	0.0238	0.0229	0.0231	0.0238	0.0229
			RMSE	0.4597	0.5195	0.4927	0.4958	0.1521	0.1543	0.1515	0.1521	0.1543	0.1514
			MRE	0.3234	0.3830	0.3604	0.3643	0.0960	0.0965	0.0949	0.0960	0.0966	0.0950
100	60	S1	Mean	1.6107	1.7124	1.6810	1.6864	1.2487	1.2285	1.2363	1.2498	1.2291	1.2370
			Bias	0.3626	0.4643	0.4329	0.4383	0.0005	−0.0197	−0.0118	0.0017	−0.0190	−0.0111
			MSE	0.1965	0.2626	0.2306	0.2345	0.0229	0.0236	0.0227	0.0232	0.0239	0.0230
			RMSE	0.4433	0.5124	0.4802	0.4842	0.1513	0.1535	0.1508	0.1523	0.1544	0.1517
			MRE	0.3112	0.3745	0.3493	0.3534	0.0965	0.0964	0.0954	0.0964	0.0970	0.0956
100	60	S2	Mean	1.6178	1.6865	1.6622	1.6668	1.2430	1.2267	1.2330	1.2425	1.2263	1.2326
			Bias	0.3696	0.4383	0.4140	0.4186	−0.0051	−0.0214	−0.0151	−0.0056	−0.0218	−0.0155
			MSE	0.1790	0.2270	0.2054	0.2085	0.0177	0.0184	0.0178	0.0178	0.0185	0.0179
			RMSE	0.4230	0.4764	0.4532	0.4566	0.1330	0.1355	0.1333	0.1333	0.1360	0.1336
			MRE	0.3014	0.3521	0.3328	0.3364	0.0847	0.0854	0.0844	0.0849	0.0856	0.0845
100	60	S3	Mean	1.6556	1.7044	1.6839	1.6875	1.2546	1.2407	1.2460	1.2551	1.2411	1.2464
			Bias	0.4075	0.4563	0.4358	0.4393	0.0065	−0.0075	−0.0022	0.0069	−0.0070	−0.0017
			MSE	0.1989	0.2362	0.2176	0.2201	0.0145	0.0147	0.0143	0.0143	0.0145	0.0142
			RMSE	0.4459	0.4861	0.4665	0.4692	0.1202	0.1211	0.1197	0.1196	0.1204	0.1191
			MRE	0.3289	0.3659	0.3496	0.3524	0.0760	0.0764	0.0756	0.0757	0.0761	0.0753
100	75	S1	Mean	1.6560	1.7134	1.6900	1.6939	1.2555	1.2412	1.2466	1.2566	1.2422	1.2476
			Bias	0.4078	0.4653	0.4419	0.4458	0.0074	−0.0069	−0.0015	0.0085	−0.0060	−0.0005
			MSE	0.2069	0.2500	0.2279	0.2306	0.0161	0.0162	0.0159	0.0163	0.0164	0.0161
			RMSE	0.4548	0.5000	0.4774	0.4802	0.1267	0.1274	0.1261	0.1277	0.1281	0.1268
			MRE	0.3273	0.3728	0.3540	0.3572	0.0831	0.0831	0.0823	0.0843	0.0840	0.0834
100	75	S2	Mean	1.6507	1.6960	1.6769	1.6803	1.2477	1.2353	1.2400	1.2473	1.2351	1.2398
			Bias	0.4026	0.4479	0.4288	0.4321	−0.0004	−0.0128	−0.0081	−0.0008	−0.0131	−0.0084
			MSE	0.1938	0.2283	0.2113	0.2136	0.0140	0.0143	0.0140	0.0141	0.0144	0.0141
			RMSE	0.4402	0.4778	0.4596	0.4622	0.1184	0.1197	0.1183	0.1186	0.1200	0.1186
			MRE	0.3245	0.3595	0.3443	0.3469	0.0748	0.0757	0.0748	0.0752	0.0761	0.0752
100	75	S3	Mean	1.6550	1.6942	1.6775	1.6803	1.2483	1.2371	1.2414	1.2491	1.2379	1.2422
			Bias	0.4068	0.4460	0.4293	0.4322	0.0002	−0.0110	−0.0068	0.0010	−0.0102	−0.0059
			MSE	0.1993	0.2288	0.2139	0.2159	0.0150	0.0152	0.0149	0.0149	0.0151	0.0148
			RMSE	0.4465	0.4784	0.4625	0.4647	0.1224	0.1235	0.1223	0.1220	0.1230	0.1218
			MRE	0.3273	0.3578	0.3445	0.3467	0.0790	0.0799	0.0790	0.0790	0.0798	0.0790

True value: Shannon entropy = 1.2481.

**Table 4:** Mean, Bias, MSE, RMSE, and MRE of ML and Bayes estimates for Rényi entropy estimates (α=0.5) at Setting I (μ=1.5, λ=2).

*n*	*m*	Sc.	Metric	Pr. MLE	Lindley			IS			MCMC		
					SE	GE	LINEX	SE	GE	LINEX	SE	GE	LINEX
30	20	S1	Mean	1.6026	1.8553	1.9126	1.8775	1.7323	1.6738	1.6834	1.7319	1.6734	1.6829
			Bias	−0.0868	0.1659	0.2232	0.1880	0.0429	−0.0156	−0.0060	0.0425	−0.0160	−0.0065
			MSE	0.1348	0.0846	0.2810	0.1511	0.0657	0.0659	0.0617	0.0655	0.0658	0.0617
			RMSE	0.3672	0.2909	0.5301	0.3887	0.2563	0.2568	0.2485	0.2560	0.2566	0.2483
			MRE	0.1742	0.1405	0.1790	0.1567	0.1207	0.1220	0.1179	0.1206	0.1216	0.1178
30	20	S2	Mean	1.6026	1.7982	1.7944	1.7874	1.7194	1.6690	1.6774	1.7229	1.6719	1.6803
			Bias	−0.0868	0.1087	0.1050	0.0980	0.0300	−0.0204	−0.0120	0.0335	−0.0175	−0.0091
			MSE	0.1201	0.0765	0.0874	0.0760	0.0696	0.0713	0.0673	0.0698	0.0713	0.0673
			RMSE	0.3466	0.2766	0.2956	0.2757	0.2639	0.2671	0.2594	0.2642	0.2669	0.2594
			MRE	0.1657	0.1330	0.1372	0.1301	0.1251	0.1268	0.1232	0.1254	0.1269	0.1232
30	20	S3	Mean	1.6191	1.7665	1.7395	1.7428	1.7136	1.6683	1.6760	1.7157	1.6698	1.6776
			Bias	−0.0703	0.0771	0.0501	0.0534	0.0242	−0.0211	−0.0134	0.0263	−0.0196	−0.0119
			MSE	0.0935	0.0733	0.0740	0.0702	0.0653	0.0675	0.0638	0.0657	0.0676	0.0639
			RMSE	0.3058	0.2707	0.2721	0.2649	0.2555	0.2597	0.2525	0.2562	0.2600	0.2528
			MRE	0.1417	0.1271	0.1253	0.1226	0.1192	0.1202	0.1171	0.1199	0.1203	0.1172
50	40	S1	Mean	1.6598	1.7527	1.7264	1.7305	1.7301	1.7002	1.7044	1.7287	1.6991	1.7033
			Bias	−0.0296	0.0633	0.0370	0.0411	0.0407	0.0108	0.0150	0.0393	0.0097	0.0139
			MSE	0.0476	0.0365	0.0321	0.0316	0.0356	0.0340	0.0331	0.0354	0.0339	0.0330
			RMSE	0.2182	0.1911	0.1791	0.1778	0.1886	0.1843	0.1819	0.1881	0.1841	0.1817
			MRE	0.1039	0.0913	0.0852	0.0847	0.0905	0.0881	0.0870	0.0906	0.0885	0.0873
50	40	S2	Mean	1.6700	1.7428	1.7182	1.7222	1.7276	1.7015	1.7053	1.7273	1.7015	1.7052
			Bias	−0.0194	0.0533	0.0288	0.0328	0.0382	0.0121	0.0159	0.0379	0.0121	0.0158
			MSE	0.0392	0.0337	0.0311	0.0306	0.0325	0.0313	0.0306	0.0330	0.0318	0.0311
			RMSE	0.1981	0.1837	0.1765	0.1749	0.1802	0.1769	0.1749	0.1816	0.1782	0.1762
			MRE	0.0967	0.0865	0.0833	0.0825	0.0856	0.0847	0.0837	0.0864	0.0856	0.0846
50	40	S3	Mean	1.6555	1.7226	1.7006	1.7043	1.7096	1.6857	1.6894	1.7089	1.6852	1.6888
			Bias	−0.0339	0.0332	0.0112	0.0149	0.0202	−0.0037	−0.0000	0.0195	−0.0042	−0.0006
			MSE	0.0442	0.0361	0.0345	0.0338	0.0360	0.0358	0.0349	0.0359	0.0357	0.0348
			RMSE	0.2103	0.1900	0.1858	0.1840	0.1896	0.1892	0.1868	0.1895	0.1890	0.1866
			MRE	0.0974	0.0889	0.0863	0.0856	0.0886	0.0879	0.0868	0.0883	0.0878	0.0867
100	60	S1	Mean	1.6293	1.7153	1.6917	1.6956	1.6961	1.6713	1.6752	1.6977	1.6722	1.6761
			Bias	−0.0601	0.0259	0.0023	0.0062	0.0067	−0.0182	−0.0143	0.0083	−0.0172	−0.0134
			MSE	0.0533	0.0369	0.0339	0.0335	0.0374	0.0374	0.0364	0.0381	0.0380	0.0369
			RMSE	0.2308	0.1922	0.1841	0.1830	0.1933	0.1933	0.1908	0.1951	0.1948	0.1922
			MRE	0.1070	0.0909	0.0871	0.0865	0.0912	0.0903	0.0893	0.0913	0.0911	0.0899
100	60	S2	Mean	1.6359	1.6936	1.6750	1.6784	1.6864	1.6670	1.6701	1.6856	1.6663	1.6694
			Bias	−0.0535	0.0042	−0.0144	−0.0110	−0.0030	−0.0224	−0.0193	−0.0038	−0.0231	−0.0200
			MSE	0.0356	0.0273	0.0270	0.0265	0.0278	0.0282	0.0276	0.0279	0.0284	0.0278
			RMSE	0.1886	0.1653	0.1643	0.1626	0.1666	0.1680	0.1661	0.1671	0.1686	0.1666
			MRE	0.0880	0.0786	0.0772	0.0766	0.0789	0.0785	0.0777	0.0790	0.0789	0.0780
100	60	S3	Mean	1.6679	1.7081	1.6923	1.6949	1.7036	1.6875	1.6900	1.7042	1.6880	1.6905
			Bias	−0.0215	0.0187	0.0029	0.0055	0.0142	−0.0019	0.0006	0.0148	−0.0014	0.0011
			MSE	0.0257	0.0224	0.0219	0.0215	0.0226	0.0224	0.0220	0.0223	0.0221	0.0218
			RMSE	0.1602	0.1497	0.1479	0.1468	0.1502	0.1496	0.1484	0.1493	0.1487	0.1475
			MRE	0.0745	0.0703	0.0691	0.0686	0.0703	0.0699	0.0694	0.0700	0.0697	0.0691
100	75	S1	Mean	1.6669	1.7148	1.6972	1.7001	1.7079	1.6904	1.6930	1.7095	1.6916	1.6942
			Bias	−0.0225	0.0254	0.0078	0.0107	0.0185	0.0010	0.0036	0.0201	0.0022	0.0048
			MSE	0.0312	0.0266	0.0255	0.0251	0.0263	0.0258	0.0254	0.0267	0.0262	0.0258
			RMSE	0.1767	0.1630	0.1596	0.1584	0.1621	0.1608	0.1595	0.1635	0.1618	0.1605
			MRE	0.0858	0.0796	0.0778	0.0772	0.0789	0.0781	0.0775	0.0803	0.0794	0.0788
100	75	S2	Mean	1.6624	1.7001	1.6856	1.6880	1.6966	1.6818	1.6841	1.6960	1.6814	1.6837
			Bias	−0.0270	0.0107	−0.0038	−0.0014	0.0072	−0.0076	−0.0053	0.0066	−0.0080	−0.0057
			MSE	0.0251	0.0218	0.0215	0.0212	0.0219	0.0219	0.0215	0.0220	0.0220	0.0216
			RMSE	0.1584	0.1476	0.1467	0.1456	0.1480	0.1479	0.1467	0.1482	0.1482	0.1470
			MRE	0.0744	0.0691	0.0687	0.0682	0.0693	0.0692	0.0686	0.0697	0.0697	0.0691
100	75	S3	Mean	1.6668	1.6989	1.6861	1.6883	1.6960	1.6830	1.6850	1.6970	1.6840	1.6860
			Bias	−0.0226	0.0095	−0.0033	−0.0011	0.0066	−0.0064	−0.0044	0.0076	−0.0054	−0.0034
			MSE	0.0264	0.0234	0.0232	0.0229	0.0237	0.0236	0.0233	0.0235	0.0235	0.0231
			RMSE	0.1625	0.1529	0.1522	0.1512	0.1538	0.1537	0.1527	0.1533	0.1531	0.1521
			MRE	0.0779	0.0731	0.0730	0.0725	0.0735	0.0737	0.0732	0.0736	0.0737	0.0732

True value: Rényi entropy (α=0.5) = 1.6894.

**Table 5:** Various confidence intervals of entropies measured under Setting I (μ=1.5, λ=2).

*n*	*m*	Sc.	Entropy	MLE (Wald)		IS (Credible)		MCMC (CI)		MCMC (HPD)	
				CP	AL	CP	AL	CP	AL	CP	AL
30	20	S1	Shannon	1.000	4.3300	0.980	0.9727	0.975	0.9632	0.960	0.9487
			Rényi	1.000	3.5685	0.985	1.2658	0.980	1.2561	0.970	1.2279
30	20	S2	Shannon	1.000	3.5037	0.965	0.9205	0.970	0.9157	0.970	0.8999
			Rényi	1.000	2.9531	0.970	1.1801	0.965	1.1763	0.970	1.1482
30	20	S3	Shannon	1.000	3.1541	0.955	0.8709	0.950	0.8750	0.935	0.8618
			Rényi	1.000	2.6849	0.970	1.1030	0.950	1.1092	0.960	1.0845
50	40	S1	Shannon	1.000	2.4827	0.995	0.6977	0.995	0.6909	0.990	0.6798
			Rényi	1.000	2.1159	0.995	0.9066	0.995	0.8982	0.995	0.8785
50	40	S2	Shannon	1.000	2.2528	0.985	0.6599	0.980	0.6511	0.975	0.6431
			Rényi	1.000	1.9353	0.990	0.8460	0.985	0.8366	0.985	0.8210
50	40	S3	Shannon	0.985	2.1081	0.965	0.6322	0.955	0.6290	0.960	0.6215
			Rényi	1.000	1.8197	0.960	0.7990	0.955	0.7946	0.960	0.7815
100	60	S1	Shannon	1.000	2.2700	0.930	0.6079	0.950	0.6324	0.945	0.6227
			Rényi	1.000	1.9410	0.930	0.7958	0.945	0.8278	0.940	0.8109
100	60	S2	Shannon	0.990	1.8672	0.970	0.5600	0.965	0.5556	0.965	0.5483
			Rényi	1.000	1.6167	0.965	0.7164	0.965	0.7110	0.975	0.6995
100	60	S3	Shannon	0.960	1.6873	0.965	0.5187	0.960	0.5193	0.960	0.5134
			Rényi	1.000	1.4657	0.965	0.6548	0.960	0.6548	0.960	0.6449
100	75	S1	Shannon	0.980	1.8272	0.965	0.5263	0.965	0.5293	0.970	0.5227
			Rényi	1.000	1.5688	0.965	0.6839	0.970	0.6907	0.970	0.6792
100	75	S2	Shannon	0.955	1.6156	0.960	0.4906	0.965	0.4842	0.950	0.4797
			Rényi	1.000	1.3987	0.970	0.6291	0.970	0.6207	0.970	0.6128
100	75	S3	Shannon	0.895	1.5021	0.945	0.4631	0.950	0.4625	0.945	0.4576
			Rényi	1.000	1.3055	0.950	0.5845	0.950	0.5834	0.950	0.5765

**Table 6:** Mean, Bias, MSE, RMSE, and MRE of ML and Bayes estimates for Shannon entropy at Setting II (μ=2, λ=1).

*n*	*m*	Sc.	Metric	Pr. MLE	Lindley			IS			MCMC		
					SE	GE	LINEX	SE	GE	LINEX	SE	GE	LINEX
30	20	S1	Mean	2.3650	2.0682	3.5531	2.7740	1.5409	1.4945	1.5093	1.5474	1.4965	1.5123
			Bias	0.8009	0.5040	1.9890	1.2098	−0.0232	−0.0696	−0.0548	−0.0167	−0.0676	−0.0518
			MSE	1.1585	22.4250	100.0158	1.9697	0.0525	0.0643	0.0566	0.0429	0.0562	0.0483
			RMSE	1.0764	4.7355	10.0008	1.4034	0.2292	0.2536	0.2379	0.2071	0.2372	0.2198
			MRE	0.5621	1.1805	1.3009	0.7892	0.1105	0.1199	0.1137	0.1041	0.1145	0.1081
30	20	S2	Mean	2.3041	2.6612	2.6675	2.6419	1.5439	1.5048	1.5158	1.5505	1.5104	1.5211
			Bias	0.7400	1.0970	1.1034	1.0778	−0.0203	−0.0593	−0.0483	−0.0136	−0.0537	−0.0430
			MSE	0.8047	1.3205	1.3752	1.2766	0.0382	0.0448	0.0411	0.0354	0.0413	0.0387
			RMSE	0.8970	1.1491	1.1727	1.1299	0.1955	0.2117	0.2027	0.1881	0.2033	0.1967
			MRE	0.4892	0.7042	0.7086	0.6919	0.0983	0.1038	0.1000	0.0955	0.1021	0.0984
30	20	S3	Mean	2.3427	2.6179	2.5721	2.5456	1.5515	1.5138	1.5242	1.5509	1.5124	1.5230
			Bias	0.7785	1.0537	1.0080	0.9815	−0.0126	−0.0503	−0.0400	−0.0132	−0.0518	−0.0412
			MSE	0.8145	1.2078	1.1261	1.0389	0.0312	0.0369	0.0338	0.0301	0.0365	0.0330
			RMSE	0.9025	1.0990	1.0612	1.0193	0.1765	0.1922	0.1837	0.1734	0.1910	0.1817
			MRE	0.5051	0.6741	0.6447	0.6275	0.0877	0.0953	0.0914	0.0867	0.0948	0.0907
50	40	S1	Mean	2.4154	2.5951	2.5443	2.5305	1.5681	1.5433	1.5495	1.5769	1.5497	1.5563
			Bias	0.8512	1.0310	0.9802	0.9664	0.0039	−0.0208	−0.0147	0.0128	−0.0144	−0.0079
			MSE	0.9080	1.1726	1.0351	0.9993	0.0286	0.0300	0.0288	0.0265	0.0280	0.0268
			RMSE	0.9529	1.0829	1.0174	0.9996	0.1690	0.1732	0.1698	0.1627	0.1674	0.1638
			MRE	0.5445	0.6661	0.6267	0.6178	0.0882	0.0905	0.0886	0.0843	0.0870	0.0851
50	40	S2	Mean	2.3571	2.5219	2.4770	2.4658	1.5459	1.5238	1.5295	1.5510	1.5273	1.5333
			Bias	0.7930	0.9577	0.9129	0.9016	−0.0182	−0.0404	−0.0346	−0.0131	−0.0368	−0.0308
			MSE	0.7691	1.0128	0.9197	0.8945	0.0319	0.0344	0.0329	0.0305	0.0332	0.0316
			RMSE	0.8770	1.0064	0.9590	0.9458	0.1786	0.1855	0.1814	0.1745	0.1822	0.1779
			MRE	0.5117	0.6133	0.5836	0.5768	0.0896	0.0920	0.0903	0.0874	0.0904	0.0886
50	40	S3	Mean	2.3883	2.5223	2.4783	2.4680	1.5503	1.5285	1.5341	1.5511	1.5285	1.5342
			Bias	0.8242	0.9581	0.9142	0.9038	−0.0138	−0.0356	−0.0301	−0.0130	−0.0356	−0.0299
			MSE	0.8247	1.0215	0.9334	0.9088	0.0330	0.0353	0.0339	0.0320	0.0346	0.0331
			RMSE	0.9081	1.0107	0.9661	0.9533	0.1816	0.1879	0.1840	0.1789	0.1860	0.1819
			MRE	0.5293	0.6128	0.5848	0.5781	0.0932	0.0958	0.0941	0.0920	0.0952	0.0934
100	60	S1	Mean	2.4331	2.5774	2.5200	2.5037	1.5512	1.5327	1.5376	1.5687	1.5436	1.5497
			Bias	0.8690	1.0132	0.9558	0.9396	−0.0130	−0.0315	−0.0266	0.0046	−0.0205	−0.0144
			MSE	0.9848	1.1552	1.0107	0.9692	0.0417	0.0436	0.0422	0.0315	0.0334	0.0320
			RMSE	0.9924	1.0748	1.0053	0.9845	0.2041	0.2087	0.2054	0.1776	0.1827	0.1788
			MRE	0.5603	0.6490	0.6111	0.6007	0.1047	0.1056	0.1042	0.0934	0.0945	0.0929
100	60	S2	Mean	2.3716	2.4923	2.4532	2.4446	1.5408	1.5252	1.5291	1.5489	1.5308	1.5352
			Bias	0.8075	0.9282	0.8891	0.8805	−0.0233	−0.0390	−0.0350	−0.0152	−0.0333	−0.0289
			MSE	0.7514	0.9377	0.8626	0.8434	0.0237	0.0249	0.0242	0.0214	0.0229	0.0221
			RMSE	0.8668	0.9683	0.9288	0.9184	0.1539	0.1579	0.1555	0.1464	0.1514	0.1487
			MRE	0.5169	0.5934	0.5684	0.5629	0.0788	0.0810	0.0798	0.0740	0.0767	0.0753
100	60	S3	Mean	2.4161	2.5058	2.4744	2.4670	1.5653	1.5503	1.5538	1.5675	1.5520	1.5556
			Bias	0.8520	0.9416	0.9102	0.9029	0.0012	−0.0138	−0.0103	0.0034	−0.0122	−0.0086
			MSE	0.8110	0.9554	0.8950	0.8789	0.0210	0.0215	0.0210	0.0207	0.0212	0.0207
			RMSE	0.9005	0.9774	0.9460	0.9375	0.1448	0.1467	0.1450	0.1438	0.1458	0.1440
			MRE	0.5447	0.6020	0.5819	0.5773	0.0766	0.0773	0.0765	0.0761	0.0769	0.0761
100	75	S1	Mean	2.4688	2.5804	2.5363	2.5243	1.5835	1.5682	1.5717	1.5900	1.5721	1.5761
			Bias	0.9046	1.0162	0.9721	0.9602	0.0193	0.0041	0.0076	0.0258	0.0080	0.0120
			MSE	0.9409	1.1117	1.0174	0.9897	0.0253	0.0253	0.0249	0.0228	0.0228	0.0224
			RMSE	0.9700	1.0544	1.0087	0.9948	0.1592	0.1590	0.1577	0.1511	0.1511	0.1496
			MRE	0.5783	0.6497	0.6215	0.6139	0.0797	0.0797	0.0790	0.0754	0.0754	0.0746
100	75	S2	Mean	2.4042	2.4907	2.4606	2.4538	1.5510	1.5380	1.5411	1.5533	1.5391	1.5425
			Bias	0.8400	0.9265	0.8964	0.8897	−0.0132	−0.0262	−0.0230	−0.0108	−0.0250	−0.0216
			MSE	0.7773	0.9169	0.8603	0.8460	0.0188	0.0196	0.0191	0.0183	0.0192	0.0187
			RMSE	0.8816	0.9575	0.9275	0.9198	0.1372	0.1400	0.1383	0.1353	0.1386	0.1368
			MRE	0.5370	0.5924	0.5731	0.5688	0.0695	0.0708	0.0699	0.0690	0.0707	0.0698
100	75	S3	Mean	2.4386	2.5103	2.4845	2.4783	1.5713	1.5592	1.5620	1.5742	1.5615	1.5643
			Bias	0.8744	0.9462	0.9204	0.9142	0.0072	−0.0049	−0.0022	0.0101	−0.0026	0.0002
			MSE	0.8333	0.9534	0.9038	0.8905	0.0177	0.0179	0.0176	0.0176	0.0178	0.0175
			RMSE	0.9129	0.9764	0.9507	0.9437	0.1332	0.1339	0.1328	0.1326	0.1333	0.1321
			MRE	0.5591	0.6049	0.5884	0.5845	0.0690	0.0688	0.0684	0.0682	0.0680	0.0676

True value: Shannon entropy = 1.5641. note: The numerical collapse of Shannon entropy values reflects the amplified MLE errors, a phenomenon explicitly noted by Giner and Smyth [34] who stated that the IG distribution is “extremely difficult to treat numerically” and that Newton–Raphson algorithm “cannot in general be guaranteed to converge”, particularly in the extreme tails. It is worth noting that Shannon entropy is highly sensitive to extreme values, making it more vulnerable to estimator instability compared to other entropy measures.

**Table 7:** Mean, Bias, MSE, RMSE, and MRE of ML and Bayes estimates for Rényi entropy estimates (α=0.5) at Setting II (μ=2, λ=1).

*n*	*m*	Sc.	Metric	Pr. MLE	Lindley			IS			MCMC		
					SE	GE	LINEX	SE	GE	LINEX	SE	GE	LINEX
30	20	S1	Mean	2.2171	2.0041	2.5696	2.5626	2.3068	2.2379	2.2334	2.3163	2.2395	2.2343
			Bias	−0.0825	−0.2956	0.2700	0.2629	0.0071	−0.0618	−0.0663	0.0166	−0.0602	−0.0653
			MSE	0.3656	13.2070	0.4980	0.4778	0.1063	0.1190	0.1127	0.0837	0.0993	0.0930
			RMSE	0.6047	3.6341	0.7057	0.6913	0.3260	0.3450	0.3358	0.2893	0.3151	0.3050
			MRE	0.2074	0.4063	0.2074	0.1967	0.1086	0.1127	0.1095	0.1009	0.1061	0.1028
30	20	S2	Mean	2.1739	2.4579	2.4434	2.4370	2.2966	2.2389	2.2339	2.3067	2.2463	2.2411
			Bias	−0.1258	0.1582	0.1437	0.1373	−0.0030	−0.0608	−0.0658	0.0071	−0.0534	−0.0586
			MSE	0.1960	0.1086	0.1021	0.1008	0.0778	0.0861	0.0823	0.0718	0.0814	0.0778
			RMSE	0.4428	0.3296	0.3195	0.3175	0.2790	0.2934	0.2868	0.2679	0.2854	0.2788
			MRE	0.1544	0.1248	0.1147	0.1103	0.0976	0.0996	0.0972	0.0944	0.0983	0.0961
30	20	S3	Mean	2.2044	2.4187	2.3757	2.3716	2.3135	2.2590	2.2536	2.3116	2.2565	2.2511
			Bias	−0.0952	0.1191	0.0760	0.0720	0.0138	−0.0407	−0.0461	0.0119	−0.0432	−0.0485
			MSE	0.1552	0.0837	0.0736	0.0786	0.0666	0.0728	0.0700	0.0636	0.0711	0.0684
			RMSE	0.3940	0.2893	0.2713	0.2803	0.2581	0.2698	0.2646	0.2522	0.2666	0.2615
			MRE	0.1356	0.1067	0.0937	0.0909	0.0879	0.0906	0.0889	0.0857	0.0897	0.0883
50	40	S1	Mean	2.2649	2.4067	2.3683	2.3618	2.3287	2.2898	2.2848	2.3442	2.3005	2.2945
			Bias	−0.0348	0.1070	0.0686	0.0621	0.0290	−0.0098	−0.0148	0.0445	0.0008	−0.0051
			MSE	0.1270	0.0863	0.0592	0.0537	0.0661	0.0661	0.0640	0.0614	0.0612	0.0591
			RMSE	0.3563	0.2937	0.2433	0.2317	0.2571	0.2570	0.2529	0.2479	0.2474	0.2430
			MRE	0.1260	0.1032	0.0896	0.0854	0.0911	0.0913	0.0898	0.0879	0.0874	0.0859
50	40	S2	Mean	2.2167	2.3460	2.3115	2.3063	2.2915	2.2584	2.2546	2.3004	2.2646	2.2603
			Bias	−0.0830	0.0463	0.0119	0.0066	−0.0082	−0.0413	−0.0451	0.0007	−0.0351	−0.0393
			MSE	0.1068	0.0705	0.0638	0.0611	0.0653	0.0680	0.0663	0.0629	0.0660	0.0643
			RMSE	0.3268	0.2656	0.2525	0.2472	0.2555	0.2607	0.2575	0.2509	0.2568	0.2536
			MRE	0.1120	0.0928	0.0875	0.0854	0.0864	0.0879	0.0868	0.0848	0.0864	0.0853
50	40	S3	Mean	2.2395	2.3438	2.3103	2.3054	2.3080	2.2763	2.2723	2.3089	2.2759	2.2718
			Bias	−0.0602	0.0441	0.0106	0.0057	0.0083	−0.0234	−0.0273	0.0093	−0.0238	−0.0279
			MSE	0.1067	0.0755	0.0707	0.0676	0.0720	0.0734	0.0715	0.0686	0.0707	0.0690
			RMSE	0.3267	0.2748	0.2659	0.2601	0.2684	0.2710	0.2674	0.2620	0.2660	0.2626
			MRE	0.1146	0.0986	0.0950	0.0928	0.0941	0.0949	0.0936	0.0926	0.0937	0.0924
100	60	S1	Mean	2.2746	2.3912	2.3492	2.3414	2.3123	2.2812	2.2774	2.3385	2.2954	2.2895
			Bias	−0.0250	0.0916	0.0495	0.0417	0.0126	−0.0185	−0.0223	0.0388	−0.0042	−0.0102
			MSE	0.1578	0.0963	0.0721	0.0661	0.1026	0.1035	0.1008	0.0777	0.0771	0.0742
			RMSE	0.3973	0.3104	0.2686	0.2570	0.3204	0.3217	0.3176	0.2788	0.2776	0.2724
			MRE	0.1392	0.1165	0.1004	0.0955	0.1126	0.1112	0.1095	0.1015	0.0992	0.0971
100	60	S2	Mean	2.2257	2.3227	2.2935	2.2895	2.2836	2.2593	2.2562	2.2973	2.2685	2.2648
			Bias	−0.0740	0.0230	−0.0062	−0.0102	−0.0161	−0.0404	−0.0434	−0.0023	−0.0311	−0.0349
			MSE	0.0748	0.0536	0.0509	0.0490	0.0537	0.0549	0.0538	0.0492	0.0505	0.0495
			RMSE	0.2735	0.2314	0.2255	0.2214	0.2317	0.2343	0.2319	0.2217	0.2247	0.2224
			MRE	0.0946	0.0810	0.0788	0.0772	0.0800	0.0813	0.0806	0.0771	0.0778	0.0770
100	60	S3	Mean	2.2652	2.3353	2.3116	2.3081	2.3161	2.2939	2.2907	2.3196	2.2965	2.2931
			Bias	−0.0344	0.0357	0.0119	0.0084	0.0164	−0.0058	−0.0090	0.0199	−0.0032	−0.0066
			MSE	0.0606	0.0495	0.0472	0.0457	0.0467	0.0465	0.0456	0.0465	0.0462	0.0452
			RMSE	0.2461	0.2224	0.2172	0.2138	0.2162	0.2157	0.2135	0.2155	0.2149	0.2127
			MRE	0.0901	0.0811	0.0794	0.0782	0.0786	0.0786	0.0777	0.0787	0.0786	0.0778
100	75	S1	Mean	2.3068	2.3955	2.3631	2.3573	2.3528	2.3274	2.3234	2.3641	2.3336	2.3286
			Bias	0.0071	0.0958	0.0634	0.0577	0.0531	0.0277	0.0237	0.0644	0.0340	0.0290
			MSE	0.0838	0.0639	0.0551	0.0520	0.0624	0.0601	0.0584	0.0569	0.0541	0.0524
			RMSE	0.2895	0.2528	0.2347	0.2281	0.2497	0.2451	0.2417	0.2386	0.2326	0.2288
			MRE	0.0982	0.0886	0.0819	0.0796	0.0858	0.0843	0.0831	0.0816	0.0795	0.0782
100	75	S2	Mean	2.2515	2.3203	2.2979	2.2947	2.3025	2.2823	2.2795	2.3067	2.2844	2.2812
			Bias	−0.0482	0.0207	−0.0018	−0.0049	0.0028	−0.0174	−0.0201	0.0070	−0.0153	−0.0184
			MSE	0.0529	0.0418	0.0405	0.0393	0.0406	0.0409	0.0402	0.0395	0.0399	0.0392
			RMSE	0.2300	0.2044	0.2012	0.1984	0.2016	0.2022	0.2005	0.1988	0.1999	0.1981
			MRE	0.0806	0.0705	0.0697	0.0688	0.0702	0.0707	0.0701	0.0686	0.0695	0.0690
100	75	S3	Mean	2.2839	2.3397	2.3203	2.3173	2.3236	2.3057	2.3031	2.3283	2.3094	2.3065
			Bias	−0.0157	0.0400	0.0206	0.0176	0.0240	0.0061	0.0034	0.0287	0.0097	0.0068
			MSE	0.0480	0.0421	0.0402	0.0391	0.0400	0.0395	0.0388	0.0400	0.0393	0.0385
			RMSE	0.2190	0.2053	0.2005	0.1978	0.2001	0.1987	0.1969	0.1999	0.1981	0.1962
			MRE	0.0773	0.0727	0.0710	0.0700	0.0711	0.0704	0.0697	0.0711	0.0702	0.0695

True value: Rényi entropy (α=0.5)=2.2997.

**Table 8:** Various confidence intervals of entropies measured under Setting II (μ=2, λ=1).

*n*	*m*	Sc.	Entropy	MLE (Wald)		IS (Credible)		MCMC (CI)		MCMC (HPD)	
				CP	AL	CP	AL	CP	AL	CP	AL
30	20	S1	Shannon	1.000	9.0775	0.915	0.9274	0.990	1.0198	0.990	1.0120
			Rényi	1.000	6.9096	0.920	1.4347	0.995	1.5931	0.995	1.5724
30	20	S2	Shannon	1.000	5.2848	0.980	0.9123	0.995	0.9442	0.995	0.9361
			Rényi	1.000	4.2827	0.985	1.3768	0.990	1.4384	0.990	1.4170
30	20	S3	Shannon	1.000	4.7285	0.985	0.8990	0.995	0.9250	0.995	0.9167
			Rényi	1.000	3.8706	0.980	1.3646	0.995	1.3874	0.995	1.3638
50	40	S1	Shannon	1.000	4.3135	0.970	0.7258	0.995	0.7963	0.995	0.7882
			Rényi	1.000	3.4898	0.955	1.1257	0.990	1.2566	0.990	1.2351
50	40	S2	Shannon	0.955	3.3654	0.940	0.7004	0.950	0.7399	0.950	0.7318
			Rényi	0.995	2.7731	0.975	1.0590	0.965	1.1304	0.965	1.1105
50	40	S3	Shannon	0.895	3.1910	0.940	0.7007	0.960	0.7247	0.955	0.7167
			Rényi	1.000	2.6382	0.940	1.0458	0.955	1.0873	0.955	1.0674
100	60	S1	Shannon	1.000	4.5180	0.835	0.5820	0.975	0.7632	0.965	0.7561
			Rényi	1.000	3.6330	0.830	0.9593	0.980	1.2459	0.960	1.2243
100	60	S2	Shannon	0.895	2.9126	0.885	0.5529	0.975	0.6527	0.975	0.6448
			Rényi	1.000	2.4063	0.910	0.8627	0.975	1.0163	0.975	0.9972
100	60	S3	Shannon	0.670	2.5535	0.960	0.5865	0.965	0.6091	0.960	0.6028
			Rényi	1.000	2.1228	0.960	0.8802	0.965	0.9168	0.975	0.9015
100	75	S1	Shannon	0.930	3.2680	0.905	0.5569	0.960	0.6554	0.945	0.6476
			Rényi	1.000	2.6619	0.885	0.8912	0.970	1.0603	0.950	1.0397
100	75	S2	Shannon	0.725	2.4843	0.930	0.5267	0.960	0.5800	0.945	0.5732
			Rényi	1.000	2.0556	0.940	0.8090	0.975	0.8963	0.975	0.8792
100	75	S3	Shannon	0.500	2.2820	0.955	0.5352	0.970	0.5529	0.960	0.5469
			Rényi	1.000	1.8972	0.965	0.7988	0.965	0.8283	0.960	0.8151

**Table 9:** Growth Hormone Therapy Data: Parameter, CI, Shannon entropy estimates and Rényi entropy estimates at α=0.5.

Scheme		*m*	μ		λ		Shannon *H*	Rényi $H_{\alpha }$
			Estimate	95% CI	Estimate	95% CI		
0% removal	S1	35	5.3057	[4.403, 6.208]	20.1311	[10.699, 29.563]	2.2443	2.5331
	S2	35	5.3057	[4.403, 6.208]	20.1311	[10.699, 29.563]	2.2443	2.5331
	S3	35	5.3057	[4.403, 6.208]	20.1311	[10.699, 29.563]	2.2443	2.5331
10% removal	S1	31	5.1840	[4.298, 6.070]	21.5931	[10.749, 32.437]	2.1888	2.4694
	S2	31	5.6278	[4.555, 6.700]	19.0071	[9.806, 28.209]	2.3420	2.6419
	S3	31	5.6823	[4.735, 6.630]	25.2381	[12.980, 37.496]	2.2577	2.5331
20% removal	S1	28	5.3686	[4.325, 6.413]	19.5151	[9.130, 29.901]	2.2706	2.5633
	S2	28	5.9031	[4.666, 7.140]	18.3109	[9.234, 27.388]	2.4168	2.7257
	S3	28	5.9890	[4.993, 6.985]	29.6103	[14.640, 44.581]	2.2710	2.5383
30% removal	S1	25	4.9556	[4.088, 5.823]	24.4811	[10.666, 38.296]	2.0819	2.3493
	S2	25	6.1370	[4.710, 7.564]	17.5208	[8.645, 26.397]	2.4811	2.7993
	S3	25	6.3098	[5.234, 7.385]	33.2309	[15.560, 50.901]	2.2994	2.5623

Note: CI = Confidence Interval (95%); Rényi entropy with α=0.5. S1: Removal at end; S2: Removal at middle; S3: Removal at beginning.

**Table 10:** Distribution comparison results for dataset I.

Dist.	AIC	AICC	BIC	HQIC	CAIC	AD Stat	AD *p*	CvM Stat	CvM *p*	KS Stat	KS *p*
IG	153.9581	154.2508	157.5264	155.2814	159.5264	0.2120	0.9868	0.0361	0.9539	0.0679	0.9790
GomLin	159.9374	160.2301	163.5058	161.2607	165.5058	0.7537	0.5148	0.1087	0.5456	0.1057	0.6704
T2HTW	162.4722	163.0722	167.8248	164.4572	170.8248	0.6748	0.5793	0.1069	0.5539	0.1172	0.5420
PLin	163.5180	163.8106	167.0863	164.8413	169.0863	0.9423	0.3885	0.1505	0.3894	0.1306	0.4061
W	162.4283	162.7210	165.9967	163.7516	167.9967	0.8742	0.4299	0.1432	0.4123	0.1307	0.4053
PRay	162.4283	162.7210	165.9967	163.7516	167.9967	0.8742	0.4298	0.1432	0.4123	0.1307	0.4052
EpW	163.1794	163.4721	166.7478	164.5028	168.7478	0.9227	0.3999	0.1484	0.3957	0.1307	0.4048
Perk	162.7648	163.0575	166.3332	164.0882	168.3332	0.9644	0.3760	0.1769	0.3179	0.1452	0.2836
Gam	162.7488	163.0415	166.3172	164.0722	168.3172	0.9872	0.3636	0.1848	0.2997	0.1473	0.2680
PMuth	166.5134	166.8061	170.0818	167.8368	172.0818	1.2760	0.2402	0.2105	0.2484	0.1473	0.2680
GExp	162.6551	162.9478	166.2235	163.9784	168.2235	1.0246	0.3442	0.1971	0.2737	0.1498	0.2508
Chen	176.6565	176.9492	180.2249	177.9798	182.2249	2.2509	0.0674	0.3652	0.0891	0.1831	0.0918

**Table 11:** Radiation Therapy Data: Parameter, CI, Shannon entropy estimates and Rényi entropy estimates at α=0.5.

Scheme		*m*	μ		λ		Shannon *H*	Rényi $H_{\alpha }$
			Estimate	95% CI	Estimate	95% CI		
0% removal	S1	44	2.2348	[1.321, 3.148]	1.1678	[0.680, 1.656]	1.6795	2.3978
	S2	44	2.2348	[1.321, 3.148]	1.1678	[0.680, 1.656]	1.6795	2.3978
	S3	44	2.2348	[1.321, 3.148]	1.1678	[0.680, 1.656]	1.6795	2.3978
10% removal	S1	40	2.1771	[1.174, 3.180]	1.1832	[0.669, 1.697]	1.6568	2.3601
	S2	40	2.4982	[1.331, 3.666]	1.1504	[0.664, 1.637]	1.7769	2.5456
	S3	40	2.4321	[1.495, 3.370]	1.5718	[0.885, 2.259]	1.7774	2.4183
20% removal	S1	35	2.0782	[1.001, 3.155]	1.2091	[0.657, 1.761]	1.6152	2.2932
	S2	35	2.8619	[1.266, 4.458]	1.1272	[0.645, 1.609]	1.8890	2.7248
	S3	35	2.7110	[1.643, 3.779]	1.9181	[1.021, 2.815]	1.8878	2.4984
30% removal	S1	31	2.0098	[0.867, 3.153]	1.2298	[0.644, 1.816]	1.5845	2.2444
	S2	31	3.2307	[1.124, 5.337]	1.1133	[0.632, 1.594]	1.9842	2.8814
	S3	31	2.9690	[1.714, 4.224]	2.0582	[1.036, 3.081]	1.9785	2.5958

Note: CI = Confidence Interval (95%); Rényi entropy with α=0.5. S1: Removal at end; S2: Removal at middle; S3: Removal at beginning.

**Table 12:** Distribution comparison results for dataset II.

Dist.	AIC	AICC	BIC	HQIC	CAIC	AD Stat	AD *p*	CvM Stat	CvM *p*	KS Stat	KS *p*
IG	160.3819	160.7569	163.4926	161.4557	165.4926	0.3774	0.8700	0.0571	0.8354	0.0915	0.9315
GExp	162.1951	162.5701	165.3058	163.2689	167.3058	0.5260	0.7193	0.0765	0.7154	0.1012	0.8657
Gam	164.2165	164.5915	167.3272	165.2903	169.3272	0.6633	0.5891	0.1045	0.5658	0.1235	0.6594
T2HTW	167.5767	168.3509	172.2428	169.1874	175.2428	0.7488	0.5184	0.1173	0.5089	0.1341	0.5545
Perk	170.9981	171.3731	174.1088	172.0719	176.1088	0.9618	0.3773	0.1237	0.4826	0.1378	0.5196
GomLin	170.9932	171.3682	174.1039	172.0670	176.1039	0.9614	0.3775	0.1236	0.4832	0.1379	0.5187
PLin	167.7614	168.1364	170.8721	168.8353	172.8721	0.8777	0.4274	0.1379	0.4304	0.1408	0.4916
W	168.9773	169.3523	172.0879	170.0511	174.0879	0.9770	0.3690	0.1518	0.3855	0.1450	0.4536
PRay	168.9772	169.3522	172.0879	170.0510	174.0879	0.9789	0.3679	0.1525	0.3835	0.1454	0.4500
EpW	169.6924	170.0674	172.8031	170.7662	174.8031	1.0181	0.3473	0.1570	0.3703	0.1458	0.4465
PMuth	171.7277	172.1027	174.8384	172.8015	176.8384	1.2326	0.2552	0.1961	0.2758	0.1573	0.3516
Chen	178.8560	179.2310	181.9667	179.9298	183.9667	1.7578	0.1256	0.2760	0.1581	0.1920	0.1515

## Data Availability

The data are available in the paper.

## Supplementary Materials

The following supporting information can be downloaded at: https://www.mdpi.com/article/10.3390/e28080871/s1.

## Abbreviations

The following abbreviations are used in this manuscript:

AL	Average lengths.
CIs	Confidence intervals.
CP	Coverage probabilities.
EpW	Epanechnikov–Weibull distribution.
Gam	Gamma distribution.
GE	General Entropy loss function.
GExp	Generalized Exponential distribution.
GomLin	Gompertz-Lindley distribution.
HPD	Highest posterior density.
IS	Importance sampling.
MLs	Maximum likelihood estimators
MRE	Mean relative error.
MSE	Mean squared error.
MCMC	Monte Carlo Markov Chain.
Perk	Perks distribution.
PLin	Power Lindley distribution.
PMuth	Power Muth distribution.
PRay	Power Rayleigh distribution.
RMSE	Root mean squared error.
Sc	Scheme.
SE	Squared Error loss function.
T2HTW	Type-II Heavy-Tailed Weibull distribution.
W	Weibull distribution.
K(.)	The Bessel function.
$E_{1}\left(.\right)$	The exponential integral.

## References

[1] Shannon C.E. (1948). A Mathematical Theory of Communication. Bell Syst. Tech. J..

[2] Rényi A. (1961). On measures of entropy and information. Proceedings of the Fourth Berkeley Symposium on Mathematical Statistics and Probability.

[3] Rass S., König S. (2018). Password security as a game of entropies. Entropy.

[4] Eun S.-K., Jung N.-S., Lee J.-J., Bae Y.-J. (2012). Uncertainty of agricultural product prices by information entropy model using probability distribution for monthly prices. J. Korean Soc. Agric. Eng..

[5] Harte D., Vere-Jones D. (2005). The entropy score and its uses in earthquake forecasting. Pure Appl. Geophys..

[6] Kamavaram S., Goseva-Popstojanova K. Entropy as a measure of uncertainty in software reliability. Proceedings of the 13th International Symposium on Software Reliability Engineering.

[7] Tweedie M.C.K. (1941). A Mathematical Investigation of Some Electrophoretic Measurements on Colloids. Master’s Thesis.

[8] Wald A. (1944). On cumulative sums of random variables. Ann. Math. Stat..

[9] Tweedie M.C.K. (1956). Some statistical properties of inverse Gaussian distributions. Va. J. Sci..

[10] Tweedie M.C.K. (1957). Statistical properties of inverse Gaussian distributions–I. Ann. Math. Stat..

[11] Tweedie M.C.K. (1957). Statistical properties of inverse Gaussian distributions–II. Ann. Math. Stat..

[12] Nath G.B. (1977). Estimation of the parameter of the inverse Gaussian distribution from generalized censored samples. Metrika.

[13] Whitmore G.A. (1983). A regression method for censored inverse-Gaussian data. Can. J. Stat..

[14] Anaya K., O’Reilly F. (2001). Test of fit for the inverse Gaussian and gamma distributions under censoring. Commun. Stat.–Theory Methods.

[15] Ismail S.A., Auda H.A. (2006). Bayesian and fiducial inference for the inverse Gaussian distribution via Gibbs sampler. J. Appl. Stat..

[16] Jia J.M., Yan Z.Z., Peng X.Y. (2017). Estimation for inverse Gaussian distribution under first-failure progressive hybrid censored samples. Filomat.

[17] Rostamian S., Nematollahi N. (2019). Estimation of stress–strength reliability in the inverse Gaussian distribution under progressively Type-II censored data. Math. Sci..

[18] Jayalath K.P., Chhikara R.S. (2022). Survival analysis for the inverse Gaussian distribution with the Gibbs sampler. J. Appl. Stat..

[19] Roy S., Pradhan B., Purakayastha A. (2022). On inference and design under progressive type-I interval censoring scheme for inverse Gaussian lifetime model. Int. J. Qual. Reliab. Manag..

[20] Bera S., Jana N. (2023). Estimating reliability parameters for inverse Gaussian distributions under complete and progressively Type-II censored samples. Qual. Technol. Quant. Manag..

[21] Xavier T., Vaisakh K.M., Sreedevi E.P. (2025). Goodness of fit tests for inverse Gaussian distribution in presence and absence of censoring. J. Stat. Comput. Simul..

[22] Kang S.B., Cho Y.S., Han J.T., Kim J. (2012). An estimation of the entropy for a double exponential distribution based on multiply Type-II censored samples. Entropy.

[23] Cho Y., Sun H., Lee K. (2014). An estimation of the entropy for a Rayleigh distribution based on doubly-generalized Type-II hybrid censored samples. Entropy.

[24] Cho Y., Sun H., Lee K. (2015). Estimating the entropy of a Weibull distribution under generalized progressive hybrid censoring. Entropy.

[25] Anis M.Z., De D. (2020). An expository note on Unit–Gompertz distribution with applications. Statistica.

[26] Hashem A.F., Alyami S.A. (2021). Inference on a new lifetime distribution under progressive Type-II censoring for a parallel-series structure. Complexity.

[27] Okasha H., Nassar M. (2022). Product of spacing estimation of entropy for inverse Weibull distribution under progressive Type-II censored data with applications. J. Taibah Univ. Sci..

[28] Shrahili M., El-Saeed A.R., Hassan A.S., Elbatal I., Elgarhy M. (2022). Estimation of entropy for log-logistic distribution under progressive Type-II censoring. J. Nanomater..

[29] Maiti K., Kayal S., Kundu D. (2022). Statistical inference on the Shannon and Rényi entropy measures of generalized exponential distribution under progressive censoring. SN Comput. Sci..

[30] Patra L.K., Bajpai S., Misra N. (2023). Inadmissibility of invariant estimator of function of scale parameter of several exponential distributions. arXiv.

[31] Ahmed E.A., El-Morshedy M., Al-Essa L.A., Eliwa M.S. (2023). Statistical inference on the entropy measures of gamma distribution under progressive censoring: EM and MCMC algorithms. Mathematics.

[32] Gong Q., Yin B. (2024). Statistical inference of entropy functions of generalized inverse exponential model under progressive Type-II censoring test. PLoS ONE.

[33] Folks J.L., Chhikara R.S. (1978). The inverse Gaussian distribution and its statistical application—A review. J. R. Stat. Soc. Ser. B.

[34] Giner G., Smyth G.K. (2016). statmod: Probability calculations for the inverse Gaussian distribution. R J..

[35] Greene W.H. (2003). Econometric Analysis.

[36] Brown L.D., Cai T.T., DasGupta A. (2001). Interval estimation for a binomial proportion. Stat. Sci..

[37] Pandey B.N., Bandyopadhyay P. (2012). Bayesian estimation of inverse Gaussian distribution. arXiv.

[38] Lindley D.V. (1980). Approximate Bayes methods. Bayesian Statistics.

[39] Nassar M.M., Eissa F.H. (2005). Bayesian estimation for the exponentiated Weibull model. Commun. Stat.–Theory Methods.

[40] Kundu D., Pradhan B. (2009). Bayesian inference and life testing plans for generalized exponential distribution. Sci. China Ser. A Math..

[41] Xu A., Tang Y. (2010). Reference analysis for Birnbaum–Saunders distribution. Comput. Stat. Data Anal..

[42] Kim C., Jung J., Chung Y. (2011). Bayesian estimation for the exponentiated Weibull model under Type II progressive censoring. Stat. Pap..

[43] Sultan H., Ahmad S.P. (2015). Bayesian approximation of generalized gamma distribution under three loss functions using Lindley’s approximation. J. Appl. Probab. Stat..

[44] Sana S., Faizan M. (2021). Bayesian estimation using Lindley’s approximation and prediction of generalized exponential distribution based on lower record values. J. Stat. Appl. Probab..

[45] Ren H., Hu X. (2023). Bayesian estimations of Shannon entropy and Rényi entropy of inverse Weibull distribution. Mathematics.

[46] Chen M.H., Shao Q.M. (1999). Monte Carlo estimation of Bayesian credible and HPD intervals. J. Comput. Graph. Stat..

[47] Hastings W.K. (1970). Monte Carlo sampling methods using Markov chains and their applications. Biometrika.

[48] Geman S., Geman D. (1984). Stochastic relaxation, Gibbs distributions, and the Bayesian restoration of images. IEEE Trans. Pattern Anal. Mach. Intell..

[49] Balakrishnan N., Sandhu R.A. (1995). A simple simulational algorithm for generating progressive Type-II censored samples. Am. Stat..

[50] Alizadeh M., Bagheri S.F., Samani E.B., Ghobadi S., Nadarajah S. (2018). Exponentiated power Lindley power series class of distributions: Theory and applications. Commun. Stat.-Simul. Comput..

