# Supplementary material for: A Computational Bayesian Framework for Entropy-Based Inference in the Inverse Gaussian Distribution Under Progressive Type-II Censoring
Source: Entropy (Basel). 2026 Aug 2;28(8):871. doi: 10.3390/e28080871 (PMC13511870; doi:10.3390/e28080871)
Supplement: Supplementary file 1 [file entropy-28-00871-s001.zip › entropy-4389620-supplementary.pdf]

1 S1

This section outlines the steps of Shannon entropy and Rényi entropy, also checking that the steps are corrected.

Shannon entropy:

$$
\begin{aligned}
H &= -\int_0^\infty f(x)\left[\ln\left(\sqrt{\frac{\lambda}{2\pi x^3}}\, e^{-\frac{\lambda(x-\mu)^2}{2\mu^2 x}}\right)\right] dx \\
&= -\int_0^\infty f(x)\left[\ln\left(\left(\frac{\lambda}{2\pi}\right)^{\frac{1}{2}} \cdot x^{-\frac{3}{2}} \cdot e^{-\frac{\lambda(x-\mu)^2}{2\mu^2 x}}\right)\right] dx \\
&= -\int_0^\infty f(x)\left[\frac{1}{2}\ln\left(\frac{\lambda}{2\pi}\right) - \frac{3}{2}\ln(x) - \frac{\lambda(x-\mu)^2}{2\mu^2 x}\right] dx \\
&= -\frac{1}{2}\ln\left(\frac{\lambda}{2\pi}\right) \underbrace{\int_0^\infty f(x)\,dx}_{\text{Integral of pdf }=1} + \frac{3}{2}\int_0^\infty f(x)\ln(x)\,dx + \frac{\lambda}{2\mu^2}\int_0^\infty f(x)\,\frac{(x-\mu)^2}{x}\,dx \\
&= -\frac{1}{2}\ln\left(\frac{\lambda}{2\pi}\right) + \frac{3}{2}\underbrace{\int_0^\infty \ln(x)\,f(x)\,dx}_{\text{Expectation}} + \frac{\lambda}{2\mu^2}\underbrace{\int_0^\infty \frac{(x-\mu)^2}{x}\,f(x)\,dx}_{\text{Expectation}} \\
&= -\frac{1}{2}\ln\left(\frac{\lambda}{2\pi}\right) + \frac{3}{2}\mathbb{E}\left[\ln(x)\right] + \frac{\lambda}{2\mu^2}\mathbb{E}\left[\frac{(x-\mu)^2}{x}\right] \qquad \to ①
\end{aligned}
$$

$$
\begin{aligned}
\mathbb{E}\left[\ln(x)\right] &= \int_0^\infty \ln(x)\,f(x)\,dx \\
&= \int_0^\infty \sqrt{\frac{\lambda}{2\pi x^3}}\, e^{-\left(\frac{\lambda(x-\mu)^2}{2\mu^2 x}\right)} \ln(x)\,dx \\
&= \int_0^\infty \sqrt{\frac{\lambda}{2\pi(\mu y)^3}}\, e^{-\left(\frac{\lambda(\mu y-\mu)^2}{2\mu^2 \mu y}\right)} \ln(\mu y)\,\mu\,dy \\
&= \int_0^\infty \sqrt{\frac{\lambda}{2\pi(\mu y)^3}}\, e^{-\left(\frac{\lambda(y-1)^2}{2\mu y}\right)} \left(\ln(\mu) + \ln(y)\right)\,\mu\,dy \\
&= \int_0^\infty \sqrt{\frac{\lambda}{2\pi(\mu y)^3}}\, e^{-\left(\frac{\lambda(y-1)^2}{2\mu y}\right)} \ln(\mu)\,\mu\,dy + \int_0^\infty \sqrt{\frac{\lambda}{2\pi(\mu y)^3}}\, e^{-\left(\frac{\lambda(y-1)^2}{2\mu y}\right)} \ln(y)\,\mu\,dy \\
&= \ln(\mu)\cdot \underbrace{\int_0^\infty \sqrt{\frac{\lambda}{2\pi x^3}}\, e^{-\left(\frac{\lambda(x-\mu)^2}{2\mu^2 x}\right)}\,dx}_{\text{Integral of pdf }=1} + \int_0^\infty \sqrt{\frac{\lambda}{2\pi(\mu y)^3}}\, e^{-\left(\frac{\lambda(y-1)^2}{2\mu y}\right)} \ln(y)\,\mu\,dy \\
&= \ln(\mu) + \int_0^\infty \sqrt{\frac{\lambda}{2\pi(\mu y)^3}}\, e^{-\left(\frac{\lambda(y-1)^2}{2\mu y}\right)} \ln(y)\,\mu\,dy \\
&= \ln(\mu) + \left(\frac{\lambda}{2\pi\,\mu^3}\right)^{1/2} \cdot \mu \int_0^\infty y^{-3/2}\, e^{-\left(\frac{\lambda(y-1)^2}{2\mu y}\right)} \ln(y)\,dy \\
&= \ln(\mu) + \left(\frac{\lambda}{2\pi\,\mu}\right)^{1/2} \int_0^\infty y^{-3/2}\, e^{-\left(\frac{\lambda(y^2-2y+1)}{2\mu y}\right)} \ln(y)\,dy \\
&= \ln(\mu) + \left(\frac{\lambda}{2\pi\,\mu}\right)^{1/2} \int_0^\infty y^{-3/2}\, e^{-\frac{\lambda}{2\mu}\left(y+\frac{1}{y}-2\right)} \ln(y)\,dy \\
&= \ln(\mu) + \left(\frac{\lambda}{2\pi\,\mu}\right)^{1/2} \int_0^\infty y^{-3/2}\, e^{-\frac{\lambda}{2\mu}\left(y+y^{-1}-2\right)} \ln(y)\,dy \\
&= \ln(\mu) + \left(\frac{\lambda}{2\pi\,\mu}\right)^{1/2} \int_{-\infty}^\infty e^{-3t/2}\, e^{-\frac{\lambda}{2\mu}\left(e^t+e^{-t}-2\right)}\, t\, e^t\,dt
\end{aligned}
$$

$$
\begin{aligned}
&\because (x-\mu) = x^2 - 2x\mu + \mu^2 \\
&\text{let: } x = \mu\, y \\
&dx = \mu\, dy \\
&x = 0 \Rightarrow y = 0 \\
&x = \infty \Rightarrow y = \infty
\end{aligned}
$$

$$
\begin{aligned}
&\text{let: } y = e^t \\
&\ln(y) = t \\
&dy = e^t\, dt \\
&x = 0 \Rightarrow y = -\infty \\
&x = \infty \Rightarrow y = \infty \\
&\cosh(t) = y + \frac{1}{y} \\
&= \frac{e^t + e^{-t}}{2}
\end{aligned}
$$

$$= \ln(\mu) + \left(\frac{\lambda}{2\pi\,\mu}\right)^{1/2} \int_{-\infty}^{\infty} e^{-t/2}\, e^{-\frac{\lambda}{2\mu}(2\cosh(t)-2)}\, t\, dt$$
$$= \ln(\mu) + \left(\frac{\lambda}{2\pi\,\mu}\right)^{1/2} \int_{-\infty}^{\infty} e^{-t/2}\, e^{-\frac{\lambda}{\mu}(\cosh(t)-1)}\, t\, dt$$
$$= \ln(\mu) + \left(\frac{\lambda}{2\pi\,\mu}\right)^{1/2} \int_{-\infty}^{\infty} e^{-t/2}\, e^{-\frac{\lambda}{\mu}\cosh(t)+\frac{\lambda}{\mu}}\, t\, dt$$
$$= \ln(\mu) + \left(\frac{\lambda}{2\pi\,\mu}\right)^{1/2} e^{\frac{\lambda}{\mu}} \int_{-\infty}^{\infty} t\, e^{-t/2}\, e^{-\frac{\lambda}{\mu}\cosh(t)}\, dt$$
$$= \ln(\mu) + \left(\frac{\lambda}{2\pi\,\mu}\right)^{1/2} e^{\frac{\lambda}{\mu}} \left[\int_{-\infty}^{0} t\, e^{-t/2}\, e^{-\frac{\lambda}{\mu}\cosh t}\, dt + \int_{0}^{\infty} t\, e^{-t/2}\, e^{-\frac{\lambda}{\mu}\cosh t}\, dt\right]$$
$$= \ln(\mu) + \left(\frac{\lambda}{2\pi\,\mu}\right)^{1/2} e^{\frac{\lambda}{\mu}} \left[\mathbb{V} + \int_{0}^{\infty} t\, e^{-t/2}\, e^{-\frac{\lambda}{\mu}\cosh t}\, dt\right] \qquad \to (2)$$

$$\mathbb{V} = \int_{-\infty}^{0} t\, e^{-t/2}\, e^{-\frac{\lambda}{\mu}\cosh t}\, dt$$
$$= \int_{\infty}^{0} (-u)\, e^{u/2}\, e^{-\frac{\lambda}{\mu}\cosh u}\, (-du)$$
$$= -\int_{0}^{\infty} u\, e^{u/2}\, e^{-\frac{\lambda}{\mu}\cosh u}\, du$$
$$= -\int_{0}^{\infty} t\, e^{t/2}\, e^{-\frac{\lambda}{\mu}\cosh t}\, dt \qquad \to (3)$$
$$\mathbb{U} = \int_{0}^{\infty} t\, e^{-t/2}\, e^{-\frac{\lambda}{\mu}\cosh t}\, dt \qquad \to (4)$$

let: $-t = u$
$$e^{-t/2} = e^{u/2}$$
$$dt = -du$$
$$t = -\infty \Rightarrow u = \infty$$
$$t = 0 \Rightarrow u = 0$$

From (3) and (4) shown above.

$$\therefore \mathbb{V} + \mathbb{U} = \int_{0}^{\infty} t\, e^{-t/2}\, e^{-\frac{\lambda}{\mu}\cosh t}\, dt - \int_{0}^{\infty} t\, e^{t/2}\, e^{-\frac{\lambda}{\mu}\cosh t}\, dt$$
$$= \int_{0}^{\infty} t e^{-\frac{\lambda}{\mu}\cosh t}(e^{-t/2} - e^{t/2}) dt$$
$$= \int_{0}^{\infty} t e^{-\frac{\lambda}{\mu}\cosh t}\left[-2\sinh\left(\frac{t}{2}\right)\right] dt$$
$$= -2\int_{0}^{\infty} t\sinh\left(\frac{t}{2}\right)\, e^{-\frac{\lambda}{\mu}\cosh t} dt$$

$$\because K_\nu(z) = \int_{0}^{\infty} e^{-z\cosh t}\cosh(\nu t)\, dt$$

Differentiating two sides with respect to order ν:

$$\therefore \frac{\partial K_\nu(z)}{\partial \nu} = \int_{0}^{\infty} t\, e^{-z\cosh t}\, \frac{\partial}{\partial \nu}[\cosh(\nu t)] dt$$
$$= \int_{0}^{\infty} t\, e^{-z\cosh t}\sinh(\nu t)\, dt$$
$$\therefore \left.\frac{\partial K_\nu(z)}{\partial \nu}\right|_{\nu=-\frac{1}{2}} = \int_{0}^{\infty} t\sinh\left(\frac{t}{2}\right)\, e^{-\frac{\lambda}{\mu}\cosh t} dt$$
$$\therefore \mathbb{V} + \mathbb{U} = -2\left.\frac{\partial K_\nu(z)}{\partial \nu}\right|_{\nu=-\frac{1}{2}} \qquad \to (5)$$

Using (5) to substitute in equation (2) shown above.

$$\therefore \mathbb{E}[\ln(x)] = \ln(\mu) + \left(\frac{\lambda}{2\pi\,\mu}\right)^{1/2} e^{\frac{\lambda}{\mu}} \left[-2\left.\frac{\partial K_\nu(z)}{\partial \nu}\right|_{\nu=-\frac{1}{2}}\right]$$

dividing and multiplying by $K_{-\frac{1}{2}}(z) = \sqrt{\dfrac{\pi}{2z}}e^{-z} = \sqrt{\dfrac{\pi\mu}{2\lambda}}e^{-\lambda/\mu}$:

$$\therefore \mathbb{E}\left[\ln(x)\right] = \ln(\mu) + \frac{1}{K_{-\frac{1}{2}}(z)}\left.\frac{\partial K_\nu(z)}{\partial\nu}\right|_{\nu=-\frac{1}{2}}$$

$$= \ln(\mu) + \left.\frac{\partial \ln K\nu(z)}{\partial\nu}\right|_{\nu=-1/2,\, z=\lambda/\mu}$$

$$\because \left.\frac{\partial K_\nu(z)}{\partial\nu}\right|_{\nu=-\frac{1}{2},\, z=\lambda/\mu} = -\sqrt{\frac{\pi}{2x}}\,E_1(2z)\,e^z$$

$$\therefore \left.\frac{\partial \ln K_\nu(z)}{\partial\nu}\right|_{\nu=-1/2,\, z=\lambda/\mu} = -e^{\frac{2\lambda}{\mu}}\;E_1\left(\frac{2\lambda}{\mu}\right)$$

$$\therefore \mathbb{E}\left[\ln(x)\right] = \ln(\mu) - e^{\frac{2\lambda}{\mu}}\;E_1\left(\frac{2\lambda}{\mu}\right) \qquad \to ⑥$$

$$\begin{aligned}
\mathbb{E}\left[\frac{(x-\mu)^2}{x}\right] &= \int_0^\infty \frac{(x-\mu)^2}{x}\,f(x)\,dx \\
&= \int_0^\infty f(x)\cdot\left(x^2-2\mu x+\mu^2\right)/x\,dx \\
&= \int_0^\infty \sqrt{\frac{\lambda}{2\pi x^3}}\left[e^{-\frac{\lambda(x-\mu)^2}{2\mu^2 x}}\right]\left[\frac{x^2-2\mu x+\mu^2}{x}\right]dx \\
&= \left(\frac{\lambda}{2\pi}\right)^{\frac{1}{2}}\int_0^\infty x^{-\frac{5}{2}}\,e^{-\frac{\lambda(x-\mu)^2}{2\mu^2 x}}\left(x^2-2\mu x+\mu^2\right)\,dx \\
&= \left(\frac{\lambda}{2\pi}\right)^{\frac{1}{2}}\left[\int_0^\infty x^{-\frac{1}{2}}\,e^{-\frac{\lambda(x-\mu)^2}{2\mu^2 x}}\,dx - 2\mu\int_0^\infty x^{-\frac{3}{2}}\,e^{-\frac{\lambda(x-\mu)^2}{2\mu^2 x}}\,dx\right. \\
&\qquad\qquad \left. + \mu^2\int_0^\infty x^{-\frac{5}{2}}\,e^{-\frac{\lambda(x-\mu)^2}{2\mu^2 x}}\,dx\right] \\
&= \left(\frac{\lambda}{2\pi}\right)^{\frac{1}{2}}\left[\mathbb{W}-2\mu\,\mathbb{H}+\mu^2\,\mathbb{G}\right] \qquad \to ⑦
\end{aligned}$$

let: $x = \mu\,y$
$dx = \mu\,dy$
$x = 0 \Rightarrow y = 0$
$x = \infty \Rightarrow y = \infty$

$$\begin{aligned}
\mathbb{W} &= \int_0^\infty x^{-\frac{1}{2}}\,e^{-\frac{\lambda(x-\mu)^2}{2\mu^2 x}}\,dx \\
&= \int_0^\infty (\mu\,y)^{-\frac{1}{2}}\,e^{-\frac{\lambda(\mu\,y-\mu)^2}{2\mu^2\mu\,y}}\,\mu\,dy \\
&= \mu^{\frac{1}{2}}\int_0^\infty y^{-\frac{1}{2}}\,e^{-\frac{\lambda(y-1)^2}{2\mu\,y}}\,dy \\
&= \mu^{\frac{1}{2}}\int_0^\infty y^{-\frac{1}{2}}\,e^{-\frac{\lambda}{2\mu}\left(y+\frac{1}{y}-2\right)}\,dy \\
&= \mu^{\frac{1}{2}}\int_0^\infty y^{-\frac{1}{2}}\left[e^{-\frac{\lambda}{2\mu}y}\cdot e^{-\frac{\lambda}{2\mu\,y}}\cdot e^{\frac{\lambda}{\mu}}\right]dy \\
&= \mu^{\frac{1}{2}}\,e^{\frac{\lambda}{\mu}}\int_0^\infty y^{-\frac{1}{2}}\left[e^{-\frac{\lambda\,y}{2\mu}-\frac{\lambda}{2\mu\,y}}\right]dy \;\Rightarrow \int_0^\infty x^{-\frac{1}{2}}\left[e^{-\gamma\,x-\frac{\beta}{x}}\right]dx = \sqrt{\frac{\pi}{\gamma}}e^{-2\sqrt{\beta\gamma}} \\
&= \mu^{\frac{1}{2}}\,e^{\frac{\lambda}{\mu}}\sqrt{\frac{\pi}{\frac{\lambda}{2\mu}}}\,e^{-2\sqrt{\frac{\lambda}{2\mu}\cdot\frac{\lambda}{2\mu}}} \\
&= \mu\sqrt{\frac{2\pi}{\lambda}} \qquad \to ⑧
\end{aligned}$$

note: $x = y$
$\gamma = \dfrac{\lambda}{2\mu},\; \beta = \dfrac{\lambda}{2\mu}$

$$\begin{aligned}
\mathbb{H} &= \int_0^\infty x^{-\frac{3}{2}}\, e^{-\frac{\lambda(x-\mu)^2}{2\mu^2 x}}\, dx \\
&= \int_0^\infty (\mu\, y)^{-\frac{3}{2}}\, e^{-\frac{\lambda(\mu\, y-\mu)^2}{2\mu^2 \mu\, y}}\, \mu\, dy \\
&= \mu^{-\frac{1}{2}} \int_0^\infty y^{-\frac{3}{2}}\, e^{-\frac{\lambda(y-1)^2}{2\mu\, y}}\, dy \\
&= \mu^{-\frac{1}{2}}\, e^{\frac{\lambda}{\mu}} \int_0^\infty y^{-\frac{3}{2}} \left[e^{-\frac{\lambda\, y}{2\mu}-\frac{\lambda}{2\mu\, y}}\right] dy \;\Rightarrow \int_0^\infty x^{\nu-1}\left[e^{-\gamma\, x-\frac{\beta}{x}}\right] dx = 2\left(\frac{\beta}{\gamma}\right)^{\nu/2} K_\nu\left(2\sqrt{\gamma\,\beta}\right) \\
&= \mu^{-\frac{1}{2}}\cdot e^{\frac{\lambda}{\mu}}\cdot 2\left(\frac{\lambda}{2\mu}\Big/\frac{\lambda}{2\mu}\right)^{-\frac{1}{2}/2}\cdot K_{-\frac{1}{2}}\left(2\sqrt{\frac{\lambda}{2\mu}\cdot\frac{\lambda}{2\mu}}\right) \\
&= 2\,\mu^{-\frac{1}{2}}\cdot e^{\frac{\lambda}{\mu}}\cdot K_{-\frac{1}{2}}\left(\frac{\lambda}{\mu}\right) \\
&= \sqrt{\frac{2\pi}{\lambda}} \qquad \rightarrow (9)
\end{aligned}$$

note: $\nu = -1/2$

$$K_{\pm\frac{1}{2}}(z) = \sqrt{\frac{\pi}{2\,z}}\, e^{-z}$$

$$\begin{aligned}
\mathbb{G} &= \int_0^\infty x^{-\frac{5}{2}}\, e^{-\frac{\lambda(x-\mu)^2}{2\mu^2 x}}\, dx \\
&= \int_0^\infty (\mu\, y)^{-\frac{5}{2}}\, e^{-\frac{\lambda(\mu\, y-\mu)^2}{2\mu^2 \mu\, y}}\, \mu\, dy \\
&= \mu^{-\frac{3}{2}} \int_0^\infty y^{-\frac{5}{2}}\, e^{-\frac{\lambda(y-1)^2}{2\mu\, y}}\, dy \\
&= \mu^{-\frac{3}{2}}\, e^{\frac{\lambda}{\mu}} \int_0^\infty y^{-\frac{5}{2}} \left[e^{-\frac{\lambda\, y}{2\mu}-\frac{\lambda}{2\mu\, y}}\right] dy \;\Rightarrow \int_0^\infty x^{\nu-1}\left[e^{-\gamma\, x-\frac{\beta}{x}}\right] dx = 2\left(\frac{\beta}{\gamma}\right)^{\nu/2} K_\nu\left(2\sqrt{\gamma\,\beta}\right) \\
&= \mu^{-\frac{3}{2}}\cdot e^{\frac{\lambda}{\mu}}\cdot 2\left(\frac{\lambda}{2\mu}\Big/\frac{\lambda}{2\mu}\right)^{-\frac{3}{2}/2}\cdot K_{-\frac{3}{2}}\left(2\sqrt{\frac{\lambda}{2\mu}\cdot\frac{\lambda}{2\mu}}\right) \\
&= 2\,\mu^{-\frac{3}{2}}\cdot e^{\frac{\lambda}{\mu}}\cdot K_{-\frac{3}{2}}\left(\frac{\lambda}{\mu}\right) \\
&= 2\,\mu^{-\frac{3}{2}}\cdot e^{\frac{\lambda}{\mu}}\cdot \sqrt{\frac{\pi}{2\left(\frac{\lambda}{\mu}\right)}}\left(1+\frac{1}{\left(\frac{\lambda}{\mu}\right)}\right) e^{-\left(\frac{\lambda}{\mu}\right)} \\
&= 2\,\mu^{-\frac{3}{2}}\sqrt{\frac{\pi\,\mu}{2\lambda}}\left(1+\frac{\mu}{\lambda}\right) \\
&= \mu^{-1}\sqrt{\frac{2\pi}{\lambda}}\left(1+\frac{\mu}{\lambda}\right) \qquad \rightarrow (10)
\end{aligned}$$

note: $\nu = -\frac{3}{2}$

$$\begin{aligned}
&\because z\,K_{\nu-1}(z) - z\,K_{\nu+1}(z) = -2\nu K_\nu(z) \\
&\therefore K_{\nu+1}(z) = K_{\nu-1}(z) + \frac{-2\nu}{z} K_\nu(z) \\
&\therefore K_{\frac{3}{2}}(z) = K_{-\frac{1}{2}}(z) + \frac{1}{z} K_{\frac{1}{2}}(z) \\
&\therefore K_{\frac{3}{2}}(z) = \sqrt{\frac{\pi}{2\,z}}\, e^{-z} + \frac{1}{z}\sqrt{\frac{\pi}{2\,z}}\, e^{-z} \\
&\therefore K_{\frac{3}{2}}(z) = \sqrt{\frac{\pi}{2z}}\left(1+\frac{1}{z}\right) e^{-z} \\
&\because K_{-\nu}(z) = K_\nu(z) \\
&\therefore K_{-\frac{3}{2}}(z) = \sqrt{\frac{\pi}{2z}}\left(1+\frac{1}{z}\right) e^{-z}
\end{aligned}$$

Using (8), (9) and (10) to substitute in equation (7) shown above.

$$\begin{aligned}
\therefore \mathbb{E}\left[\frac{(x-\mu)^2}{x}\right] &= \left(\frac{\lambda}{2\pi}\right)^{\frac{1}{2}}\left[\mathbb{W} - 2\mu\,\mathbb{H} + \mu^2\,\mathbb{G}\right] \\
&= \sqrt{\frac{\lambda}{2\pi}}\left[\mu\sqrt{\frac{2\pi}{\lambda}} - 2\mu\sqrt{\frac{2\pi}{\lambda}} + \mu^2\left(\mu^{-1}\sqrt{\frac{2\pi}{\lambda}}\left(1+\frac{\mu}{\lambda}\right)\right)\right] \\
&= \frac{\mu^2}{\lambda} \qquad \rightarrow (11)
\end{aligned}$$

Then using (6) and (11) to substitute in equation (1) shown above to obtain the Shannon entropy.

$$\therefore H = -\frac{1}{2}\ln\left(\frac{\lambda}{2\pi}\right) + \frac{3}{2}\left[\ln(\mu) - e^{\frac{2\lambda}{\mu}}\ E_1\left(\frac{2\lambda}{\mu}\right)\right] + \frac{\lambda}{2\mu^2}\left(\frac{\mu^2}{\lambda}\right)$$
$$= \frac{1}{2}\ln\frac{2\pi e}{\lambda} + \frac{3}{2}\ln\mu - \frac{3}{2}e^{\frac{2\lambda}{\mu}}E_1\left(\frac{2\lambda}{\mu}\right)$$

note:
$$\frac{1}{2}\ln\frac{2\pi}{\lambda} + \frac{1}{2} = \frac{1}{2}\ln\frac{2\pi}{\lambda} + \frac{1}{2}\ln e$$
$$= \frac{1}{2}\left(\ln\frac{2\pi}{\lambda} + \ln e\right)$$
$$= \frac{1}{2}\ln\left(\frac{2\pi}{\lambda}\cdot e\right)$$
$$= \frac{1}{2}\ln\frac{2\pi e}{\lambda}$$

Rényi entropy:

$$H_\alpha = \frac{1}{1-\alpha}\ln\left(\int_0^\infty \left[\sqrt{\frac{\lambda}{2\pi x^3}}\,e^{-\frac{\lambda(x-\mu)^2}{2\mu^2 x}}\right]^\alpha dx\right)$$
$$= \frac{1}{1-\alpha}\ln\left(\int_0^\infty \left[\left(\frac{\lambda}{2\pi}\right)^{\frac{1}{2}} x^{-\frac{3}{2}}e^{-\frac{\lambda(x-\mu)^2}{2\mu^2 x}}\right]^\alpha dx\right)$$
$$= \frac{1}{1-\alpha}\ln\left(\int_0^\infty \left(\frac{\lambda}{2\pi}\right)^{\frac{\alpha}{2}} x^{-\frac{3\alpha}{2}}e^{-\frac{\alpha\lambda(x-\mu)^2}{2\mu^2 x}}\,dx\right)$$
$$= \frac{1}{1-\alpha}\ln\left(\left(\frac{\lambda}{2\pi}\right)^{\frac{\alpha}{2}}\int_0^\infty x^{-\frac{3\alpha}{2}}e^{-\frac{\alpha\lambda x}{2\mu^2}+\frac{\alpha\lambda}{\mu}-\frac{\alpha\lambda}{2x}}\,dx\right)$$
$$= \frac{1}{1-\alpha}\left(\frac{\alpha}{2}\ln\left(\frac{\lambda}{2\pi}\right) + \ln\left[e^{\frac{\alpha\lambda}{\mu}}\int_0^\infty x^{-\frac{3\alpha}{2}}e^{-\frac{\alpha\lambda x}{2\mu^2}-\frac{\alpha\lambda}{2x}}\,dx\right]\right)$$
$$= \frac{1}{1-\alpha}\left(\frac{\alpha}{2}\ln\left(\frac{\lambda}{2\pi}\right) + \frac{\alpha\lambda}{\mu} + \ln\left[\int_0^\infty x^{-\frac{3\alpha}{2}}e^{-\frac{\alpha\lambda x}{2\mu^2}-\frac{\alpha\lambda}{2x}}\,dx\right]\right)$$
$$= \frac{1}{1-\alpha}\left(\frac{\alpha}{2}\ln\left(\frac{\lambda}{2\pi}\right) + \frac{\alpha\lambda}{\mu} + \ln\left[2\mu^{1-\frac{3\alpha}{2}}K_{1-\frac{3\alpha}{2}}\left(\frac{\alpha\lambda}{\mu}\right)\right]\right)$$
$$= \frac{1}{1-\alpha}\left[\frac{\alpha}{2}\ln\left(\frac{\lambda}{2\pi}\right) + \left(1-\frac{3\alpha}{2}\right)\ln(\mu) + \ln(2) + \frac{\alpha\lambda}{\mu} + \ln K_{1-\frac{3\alpha}{2}}\left(\frac{\alpha\lambda}{\mu}\right)\right]$$

Proof:

To prove that $\lim_{\alpha\to 1} H_\alpha = H$, we follow the following steps:

$$\lim_{\alpha\to 1} f(\alpha) = \lim_{\alpha\to 1}\left[\frac{1}{1-\alpha}\left[\frac{\alpha}{2}\ln\left(\frac{\lambda}{2\pi}\right) + \left(1-\frac{3\alpha}{2}\right)\ln(\mu) + \ln(2) + \frac{\alpha\lambda}{\mu} + \ln K_{1-\frac{3\alpha}{2}}\left(\frac{\alpha\lambda}{\mu}\right)\right]\right]$$

First, using direct substitution: $(\alpha \to 1)$

$$\text{denominator} = 1-\alpha = 1-1 = 0$$

$$\text{numerator} = \frac{1}{2}\ln\left(\frac{\lambda}{2\pi}\right) + \left(1-\frac{3}{2}\right)\ln(\mu) + \ln(2) + \frac{\lambda}{\mu} + \ln\left(K_{1-\frac{3}{2}}\left(\frac{\lambda}{\mu}\right)\right)$$
$$= \frac{1}{2}\ln\left(\frac{\lambda}{2\pi}\right) + \left(1-\frac{3}{2}\right)\ln(\mu) + \ln(2) + \frac{\lambda}{\mu} + \ln\left(\sqrt{\frac{\pi\mu}{2\lambda}}\,e^{-\frac{\lambda}{\mu}}\right)$$
$$= 0$$

$$\therefore \frac{\text{numerator}}{\text{denominator}} = \frac{0}{0}$$

Second, using L'Hôpital's rule:

$$\lim_{\alpha\to 1} f(\alpha) = \lim_{\alpha\to 1} \frac{N(\alpha)}{1-\alpha} = \lim_{\alpha\to 1} \frac{N'(\alpha)}{-1} = N'(1)$$

Differentiate both the numerator and the denominator with respect to α;

$$N(\alpha) = \frac{\alpha}{2}\ln\left(\frac{\lambda}{2\pi}\right) + \left(1-\frac{3\alpha}{2}\right)\ln(\mu) + \ln(2) + \frac{\alpha\lambda}{\mu} + \ln\left(K_{\nu(\alpha)}(z(\alpha))\right)$$

$$N'(\alpha) = \frac{1}{2}\ln\left(\frac{\lambda}{2\pi}\right) + \frac{\lambda}{\mu} - \frac{3}{2}\ln\mu + \frac{d}{d\alpha}\ln\left(K_{\nu(\alpha)}(z(\alpha))\right)$$

Using the chain rule:

$$\frac{d}{d\alpha}\ln K_\nu(z) = \frac{1}{K_\nu(z)}\left(\frac{\partial K_\nu}{\partial \nu}\cdot\frac{d\nu}{d\alpha} + \frac{\partial K_\nu}{\partial z}\cdot\frac{dz}{d\alpha}\right)$$

Let: $\nu(\alpha) = 1 - \dfrac{3\alpha}{2}$

$\therefore \nu'(\alpha) = -\dfrac{3}{2}$

, $z(\alpha) = \dfrac{\alpha\lambda}{\mu}$

$\therefore z'(\alpha) = \dfrac{\lambda}{\mu}$

when: $\alpha = 1$,

$\therefore \nu = -\dfrac{1}{2},\ z = \dfrac{\lambda}{\mu}$

So:

$$\frac{d}{d\alpha}\ln K_\nu(z) = -\frac{3}{2}\cdot\frac{1}{K_\nu(z)}\frac{\partial K_\nu}{\partial \nu} + \frac{\lambda}{\mu}\cdot\frac{1}{K_\nu(z)}\frac{\partial K_\nu}{\partial z}$$

$$\because z\frac{d}{dz}K_\nu(z) + \nu K_\nu(z) = -zK_{\nu-1}(z)$$

$$\frac{d}{dz}K_\nu(z) = -K_{\nu-1}(z) - \frac{\nu}{z}K_\nu(z)$$

$$\therefore \frac{1}{K_\nu(z)}\frac{\partial K_\nu}{\partial z} = -\frac{K_{\nu-1}(z)}{K_\nu(z)} - \frac{\nu}{z},$$

$$= \left[-\frac{K{-3/2}}{K_{-1/2}} - \frac{-1/2}{\lambda/\mu}\right]$$

$$\because \frac{1}{K_\nu(z)}\frac{\partial K_\nu}{\partial \nu}\bigg|_{\alpha=1} = \frac{\partial \ln K\nu(z)}{\partial \nu}\bigg|_{\nu=-1/2,\, z=\lambda/\mu}$$

$$\therefore \frac{d}{d\alpha}\ln K_\nu(z)\bigg|_{\alpha=1} = -\frac{3}{2}\left[\frac{\partial \ln K\nu(z)}{\partial \nu}\bigg|_{\nu=-1/2,\, z=\lambda/\mu}\right] + \frac{\lambda}{\mu}\left[-\frac{K{-3/2}}{K_{-1/2}} - \frac{-1/2}{\lambda/\mu}\right]$$

$$= -\frac{3}{2}\frac{\partial \ln K\nu(z)}{\partial \nu}\bigg|_{\nu=-1/2,\, z=\lambda/\mu} - \frac{\lambda}{\mu} - \frac{1}{2}$$

$$\therefore N'(1) = \frac{1}{2}\ln\left(\frac{\lambda}{2\pi}\right) + \frac{\lambda}{\mu} - \frac{3}{2}\ln\mu - \frac{3}{2}\frac{\partial \ln K\nu(z)}{\partial \nu}\bigg|_{\nu=-1/2,\, z=\lambda/\mu} - \frac{\lambda}{\mu} - \frac{1}{2}$$

$$= \frac{1}{2}\ln\left(\frac{\lambda}{2\pi}\right) - \frac{3}{2}\ln\mu - \frac{3}{2}\frac{\partial \ln K\nu(z)}{\partial \nu}\bigg|_{\nu=-1/2,\, z=\lambda/\mu} - \frac{1}{2}$$

$$\therefore \lim_{\alpha\to 1} f(\alpha) = \frac{N'(1)}{-1} = -\left[\frac{1}{2}\ln\left(\frac{\lambda}{2\pi}\right) - \frac{3}{2}\ln\mu - \frac{3}{2}\frac{\partial \ln K\nu(z)}{\partial \nu}\bigg|_{\nu=-1/2,\, z=\lambda/\mu} - \frac{1}{2}\right]$$

$$= \frac{1}{2}\ln\frac{2\pi}{\lambda} + \frac{3}{2}\ln\mu + \frac{1}{2} + \frac{3}{2}\frac{\partial \ln K_\nu(z)}{\partial \nu}\bigg|_{\nu=-1/2,\, z=\lambda/\mu}$$

$$= \frac{1}{2}\ln\frac{2\pi e}{\lambda} + \frac{3}{2}\ln\mu - \frac{3}{2}e^{\frac{2\lambda}{\mu}}E_1\left(\frac{2\lambda}{\mu}\right)$$

$$\therefore \lim_{\alpha\to 1} H_\alpha = H$$

The integral $K_\nu(z) = \int_0^\infty e^{-z\cosh t}\cosh(\nu t)\, dt$ is expressed in terms of the modified Bessel function of the second kind via its integral representation (Edition (2007), Eq. 8.432-1, p. 917). The evaluation at half-integer order employs the closed form $K_{\pm\frac{1}{2}}(z)$ (Edition, 2007, Eq. 8.469-3, p. 925), we also used the following forms $z\,K_{\nu-1}(z) - z\,K_{\nu+1}(z) = -2\nu K_\nu(z)$ (Edition, 2007, Eq. 8.486-10, p. 929), $z\frac{d}{dz}K_\nu(z) + \nu K_\nu(z) = -zK_{\nu-1}(z)$ (Edition, 2007, Eq. 8.486-12, p. 929) and $K_{-\nu}(z) = K_\nu(z)$ (Edition, 2007, Eq. 8.486-16, p. 929), we also used the following equations $\int_0^\infty x^{\nu-1}\left[e^{-\gamma x - \frac{\beta}{x}}\right] dx = 2\left(\frac{\beta}{\gamma}\right)^{\nu/2} K_\nu\left(2\sqrt{\gamma\beta}\right)$ (Edition, 2007, Eq. 3.471-9, p. 368) and $\int_0^\infty x^{-\frac{1}{2}}\left[e^{-\gamma x - \frac{\beta}{x}}\right] dx = \sqrt{\frac{\pi}{\gamma}}e^{-2\sqrt{\beta\gamma}}$ (Edition, 2007, Eq. 3.471-15, p. 369).

2 S2

This section outlines the steps involved in solving Lindley derivatives. Bayesian estimators of the entropies (Shannon and Rényi) under the squared error loss function (SE), the general entropy loss function (GE) and the LINEX loss function, respectively:

♦ Bayesian estimators of the Shannon entropy under the SE loss function. In that case, $u(\mu,\lambda) = H$, thus;

$$\hat{u}_{S.\mu} = \left(\frac{3\lambda}{\mu^2}\right) e^{\frac{2\lambda}{\mu}} E_1\left(\frac{2\lambda}{\mu}\right), \qquad \hat{u}_{S.\lambda} = \frac{1}{\lambda} - \left(\frac{3}{\mu}\right) e^{\frac{2\lambda}{\mu}} E_1\left(\frac{2\lambda}{\mu}\right),$$

$$\hat{u}_{S.\mu\mu} = \frac{3}{2\mu^2}\left[\frac{2\lambda}{\mu} - \left(\frac{4\lambda}{\mu} + \left(\frac{2\lambda}{\mu}\right)^2\right) e^{\frac{2\lambda}{\mu}} E_1\left(\frac{2\lambda}{\mu}\right)\right], \hat{u}_{S.\lambda\lambda} = \frac{-1}{\lambda^2}\left[1 - \frac{3\lambda}{\mu} + 6\left(\frac{\lambda}{\mu}\right)^2 e^{\frac{2\lambda}{\mu}} E_1\left(\frac{2\lambda}{\mu}\right)\right],$$

$$\hat{u}_{S.\mu\lambda} = \hat{u}_{S.\lambda\mu} = \frac{3}{2\lambda\mu}\left[\left(\frac{2\lambda}{\mu} + \left(\frac{2\lambda}{\mu}\right)^2\right) e^{\frac{2\lambda}{\mu}} E_1\left(\frac{2\lambda}{\mu}\right) - \frac{2\lambda}{\mu}\right].$$

♦ Bayesian estimators of the Rényi entropy under the SE loss function. In that case, $u(\mu,\lambda) = H_\alpha$, thus;

$$\hat{u}_{R.\mu} = \frac{1}{1-\alpha}\left[\left(1 - \frac{3\alpha}{2}\right)\frac{1}{\mu} - \frac{\alpha\lambda}{\mu^2} - \frac{\alpha\lambda}{\mu^2}\left(\frac{K'_{1-\frac{3\alpha}{2}}\left(\frac{\alpha\lambda}{\mu}\right)}{K_{1-\frac{3\alpha}{2}}\left(\frac{\alpha\lambda}{\mu}\right)}\right)\right],$$

$$\hat{u}_{R.\mu\mu} = \frac{1}{1-\alpha}\left[\left(\frac{3\alpha}{2} - 1\right)\frac{1}{\mu^2} + \frac{\alpha\lambda}{\mu^3} - \frac{2\alpha\lambda}{\mu^3}\frac{K'_{1-\frac{3\alpha}{2}}\left(\frac{\alpha\lambda}{\mu}\right)}{K_{1-\frac{3\alpha}{2}}\left(\frac{\alpha\lambda}{\mu}\right)} + \frac{\alpha^2\lambda^2}{\mu^4}\left[\frac{K''_{1-\frac{3\alpha}{2}}\left(\frac{\alpha\lambda}{\mu}\right)}{K_{1-\frac{3\alpha}{2}}\left(\frac{\alpha\lambda}{\mu}\right)} - \left(\frac{K'_{1-\frac{3\alpha}{2}}\left(\frac{\alpha\lambda}{\mu}\right)}{K_{1-\frac{3\alpha}{2}}\left(\frac{\alpha\lambda}{\mu}\right)}\right)^2\right]\right],$$

$$\hat{u}_{R.\lambda} = \frac{1}{1-\alpha}\left[\frac{\alpha}{2\lambda} + \frac{\alpha}{\mu} - \frac{\alpha}{\mu}\left(\frac{K'_{1-\frac{3\alpha}{2}}\left(\frac{\alpha\lambda}{\mu}\right)}{K_{1-\frac{3\alpha}{2}}\left(\frac{\alpha\lambda}{\mu}\right)}\right)\right],$$

$$\hat{u}_{R.\lambda\lambda} = \frac{1}{1-\alpha}\left[-\frac{\alpha}{2\lambda^2} - \left(\frac{\alpha}{\mu}\right)^2\left[\frac{K''_{1-\frac{3\alpha}{2}}\left(\frac{\alpha\lambda}{\mu}\right)}{K_{1-\frac{3\alpha}{2}}\left(\frac{\alpha\lambda}{\mu}\right)} - \left(\frac{K'_{1-\frac{3\alpha}{2}}\left(\frac{\alpha\lambda}{\mu}\right)}{K_{1-\frac{3\alpha}{2}}\left(\frac{\alpha\lambda}{\mu}\right)}\right)^2\right]\right],$$

$$\hat{u}_{R.\mu\lambda} = \hat{u}_{R.\lambda\mu} = \frac{1}{1-\alpha}\left[-\frac{\alpha}{\mu^2} - \frac{\alpha}{\mu^2}\left(\frac{K'_{1-\frac{3\alpha}{2}}\left(\frac{\alpha\lambda}{\mu}\right)}{K_{1-\frac{3\alpha}{2}}\left(\frac{\alpha\lambda}{\mu}\right)}\right) - \frac{\alpha^2\lambda}{\mu^3}\left[\frac{K''_{1-\frac{3\alpha}{2}}\left(\frac{\alpha\lambda}{\mu}\right)}{K_{1-\frac{3\alpha}{2}}\left(\frac{\alpha\lambda}{\mu}\right)} - \left(\frac{K'_{1-\frac{3\alpha}{2}}\left(\frac{\alpha\lambda}{\mu}\right)}{K_{1-\frac{3\alpha}{2}}\left(\frac{\alpha\lambda}{\mu}\right)}\right)^2\right]\right].$$

- Bayesian estimators of the Shannon entropy under the GE loss function. In that case, $u(\mu,\lambda) = H^{-q}$, thus;

$$\hat{u}_{S.\mu} = -qH^{-q-1}\left[\frac{3\lambda}{\mu^2} e^{\frac{2\lambda}{\mu}} E_1\left(\frac{2\lambda}{\mu}\right)\right], \qquad \hat{u}_{S.\lambda} = -qH^{-q-1}\left[\frac{1}{\lambda} - \frac{3}{\mu} e^{\frac{2\lambda}{\mu}} E_1\left(\frac{2\lambda}{\mu}\right)\right],$$

$$\hat{u}_{S.\mu\mu} = qH^{-q-2}\left[(q+1)\left\{\frac{3\lambda}{\mu^2} e^{\frac{2\lambda}{\mu}} E_1\left(\frac{2\lambda}{\mu}\right)\right\}^2 - \frac{3H}{2\mu^2}\left\{\frac{2\lambda}{\mu} - \left(\frac{4\lambda}{\mu} + \left(\frac{2\lambda}{\mu}\right)^2\right) e^{\frac{2\lambda}{\mu}} E_1\left(\frac{2\lambda}{\mu}\right)\right\}\right],$$

$$\hat{u}_{S.\lambda\lambda} = qH^{-q-2}\left[\frac{(q+1)}{\lambda^2}\left\{1 - \frac{3\lambda}{\mu} e^{\frac{2\lambda}{\mu}} E_1\left(\frac{2\lambda}{\mu}\right)\right\}^2 - \frac{H}{\lambda^2}\left\{-1 + \frac{3\lambda}{\mu} - 6\left(\frac{\lambda}{\mu}\right)^2 e^{\frac{2\lambda}{\mu}} E_1\left(\frac{2\lambda}{\mu}\right)\right\}\right],$$

$$\hat{u}_{S.\mu\lambda} = qH^{-q-2}\left[\frac{3\,(q+1)\,\lambda}{\mu^2} e^{\frac{2\lambda}{\mu}} E_1\left(\frac{2\lambda}{\mu}\right)\left\{1 - \frac{3\lambda}{\mu} e^{\frac{2\lambda}{\mu}} E_1\left(\frac{2\lambda}{\mu}\right)\right\}\right.$$

$$-\frac{3H}{2\lambda\mu}\left\{\left(\frac{2\lambda}{\mu}+\left(\frac{2\lambda}{\mu}\right)^2\right)e^{\frac{2\lambda}{\mu}}E_1\left(\frac{2\lambda}{\mu}\right)-\frac{2\lambda}{\mu}\right\}\Bigg].$$

- Bayesian estimators of the Rényi entropy under the GE loss function. In that case, $u(\mu,\lambda)=H_\alpha^{-q}$, thus;

$$\hat{u}_{R.\mu}=-q\,H_\alpha^{-q-1}\left[\frac{1}{1-\alpha}\left[\left(1-\frac{3\alpha}{2}\right)\frac{1}{\mu}-\frac{\alpha\lambda}{\mu^2}-\frac{\alpha\lambda}{\mu^2}\left(\frac{K'_{1-\frac{3\alpha}{2}}\left(\frac{\alpha\lambda}{\mu}\right)}{K_{1-\frac{3\alpha}{2}}\left(\frac{\alpha\lambda}{\mu}\right)}\right)\right]\right],$$

$$\hat{u}_{R.\mu\mu}=\frac{(q^2+q)\,H_\alpha^{-q-2}}{[1-\alpha]^2}\left[\left(1-\frac{3\alpha}{2}\right)\frac{1}{\mu}-\frac{\alpha\lambda}{\mu^2}-\frac{\alpha\lambda}{\mu^2}\left(\frac{K'_{1-\frac{3\alpha}{2}}\left(\frac{\alpha\lambda}{\mu}\right)}{K_{1-\frac{3\alpha}{2}}\left(\frac{\alpha\lambda}{\mu}\right)}\right)\right]^2$$
$$-\frac{q\,H_\alpha^{-q-1}}{1-\alpha}\left[\left(\frac{3\alpha}{2}-1\right)\frac{1}{\mu^2}+\frac{\alpha\lambda}{\mu^3}-\frac{2\alpha\lambda}{\mu^3}\left(\frac{K'_{1-\frac{3\alpha}{2}}\left(\frac{\alpha\lambda}{\mu}\right)}{K_{1-\frac{3\alpha}{2}}\left(\frac{\alpha\lambda}{\mu}\right)}\right)\right.$$
$$\left.+\frac{\alpha^2\lambda^2}{\mu^4}\left[\frac{K''_{1-\frac{3\alpha}{2}}\left(\frac{\alpha\lambda}{\mu}\right)}{K_{1-\frac{3\alpha}{2}}\left(\frac{\alpha\lambda}{\mu}\right)}-\left(\frac{K'_{1-\frac{3\alpha}{2}}\left(\frac{\alpha\lambda}{\mu}\right)}{K_{1-\frac{3\alpha}{2}}\left(\frac{\alpha\lambda}{\mu}\right)}\right)^2\right]\right],$$

$$\hat{u}_{R.\lambda}=-q\,H_\alpha^{-q-1}\left[\frac{1}{1-\alpha}\left[\frac{\alpha}{2\lambda}+\frac{\alpha}{\mu}-\frac{\alpha}{\mu}\left(\frac{K'_{1-\frac{3\alpha}{2}}\left(\frac{\alpha\lambda}{\mu}\right)}{K_{1-\frac{3\alpha}{2}}\left(\frac{\alpha\lambda}{\mu}\right)}\right)\right]\right],$$

$$\hat{u}_{R.\lambda\lambda}=\frac{(q^2+q)\,H_\alpha^{-q-2}}{[1-\alpha]^2}\left[\frac{\alpha}{2\lambda}+\frac{\alpha}{\mu}-\frac{\alpha}{\mu}\left(\frac{K'_{1-\frac{3\alpha}{2}}\left(\frac{\alpha\lambda}{\mu}\right)}{K_{1-\frac{3\alpha}{2}}\left(\frac{\alpha\lambda}{\mu}\right)}\right)\right]^2$$
$$-\frac{q\,H_\alpha^{-q-1}}{1-\alpha}\left[-\frac{\alpha}{2\lambda^2}-\left(\frac{\alpha}{\mu}\right)^2\left[\frac{K''_{1-\frac{3\alpha}{2}}\left(\frac{\alpha\lambda}{\mu}\right)}{K_{1-\frac{3\alpha}{2}}\left(\frac{\alpha\lambda}{\mu}\right)}-\left(\frac{K'_{1-\frac{3\alpha}{2}}\left(\frac{\alpha\lambda}{\mu}\right)}{K_{1-\frac{3\alpha}{2}}\left(\frac{\alpha\lambda}{\mu}\right)}\right)^2\right]\right],$$

$$\hat{u}_{R.\mu\lambda}=\hat{u}_{R.\lambda\mu}=\frac{(q^2+q)\,H_\alpha^{-q-2}}{(1-\alpha)^2}\left[\left(1-\frac{3\alpha}{2}\right)\frac{1}{\mu}-\frac{\alpha\lambda}{\mu^2}-\frac{\alpha\lambda}{\mu^2}\left(\frac{K'_{1-\frac{3\alpha}{2}}\left(\frac{\alpha\lambda}{\mu}\right)}{K_{1-\frac{3\alpha}{2}}\left(\frac{\alpha\lambda}{\mu}\right)}\right)\right]$$
$$\left[\frac{\alpha}{2\lambda}+\frac{\alpha}{\mu}-\frac{\alpha}{\mu}\left(\frac{K'_{1-\frac{3\alpha}{2}}\left(\frac{\alpha\lambda}{\mu}\right)}{K_{1-\frac{3\alpha}{2}}\left(\frac{\alpha\lambda}{\mu}\right)}\right)\right]-\frac{q\,H_\alpha^{-q-1}}{1-\alpha}\left[-\frac{\alpha}{\mu^2}\right.$$
$$\left.-\frac{\alpha}{\mu^2}\left(\frac{K'_{1-\frac{3\alpha}{2}}\left(\frac{\alpha\lambda}{\mu}\right)}{K_{1-\frac{3\alpha}{2}}\left(\frac{\alpha\lambda}{\mu}\right)}\right)-\frac{\alpha^2\lambda}{\mu^3}\left[\frac{K''_{1-\frac{3\alpha}{2}}\left(\frac{\alpha\lambda}{\mu}\right)}{K_{1-\frac{3\alpha}{2}}\left(\frac{\alpha\lambda}{\mu}\right)}-\left(\frac{K'_{1-\frac{3\alpha}{2}}\left(\frac{\alpha\lambda}{\mu}\right)}{K_{1-\frac{3\alpha}{2}}\left(\frac{\alpha\lambda}{\mu}\right)}\right)^2\right]\right].$$

▲ Bayesian estimators of the Shannon entropy under the LINEX loss function. In that case, $u(\mu,\lambda)=e^{-H\,p}$, thus;

$$\hat{u}_{S.\mu}=-pe^{-pH}\left[\frac{3\lambda}{\mu^2}\,e^{\frac{2\lambda}{\mu}}E_1\left(\frac{2\lambda}{\mu}\right)\right],\qquad \hat{u}_{S.\lambda}=-pe^{-pH}\left[\frac{1}{\lambda}-\frac{3}{\mu}\,e^{\frac{2\lambda}{\mu}}E_1\left(\frac{2\lambda}{\mu}\right)\right],$$

$$\hat{u}_{S.\mu\mu}=pe^{-pH}\left[p\left\{\frac{3\lambda}{\mu^2}\,e^{\frac{2\lambda}{\mu}}\,E_1\left(\frac{2\lambda}{\mu}\right)\right\}^2-\frac{3}{2\mu^2}\left\{\frac{2\lambda}{\mu}-\left[\frac{4\lambda}{\mu}+\left(\frac{2\lambda}{\mu}\right)^2\right]e^{\frac{2\lambda}{\mu}}E_1\left(\frac{2\lambda}{\mu}\right)\right\}\right],$$

$$\hat{u}_{S.\lambda\lambda}=pe^{-pH}\left[\frac{p}{\lambda^2}\left\{1-\frac{3\lambda}{\mu}e^{\frac{2\lambda}{\mu}}E_1\left(\frac{2\lambda}{\mu}\right)\right\}^2-\frac{1}{\lambda^2}\left\{-1+\frac{3\lambda}{\mu}-6\left(\frac{\lambda}{\mu}\right)^2e^{\frac{2\lambda}{\mu}}E_1\left(\frac{2\lambda}{\mu}\right)\right\}\right],$$

$$\hat{u}_{S.\mu\lambda}=pe^{-pH}\left[\frac{3\,p\,\lambda}{\mu^2}e^{\frac{2\lambda}{\mu}}E_1\left(\frac{2\lambda}{\mu}\right)\left\{1-\frac{3\lambda}{\mu}e^{\frac{2\lambda}{\mu}}E_1\left(\frac{2\lambda}{\mu}\right)\right\}\right.$$
$$\left.-\frac{3}{2\lambda\mu}\left\{\left(\frac{2\lambda}{\mu}+\left(\frac{2\lambda}{\mu}\right)^2\right)e^{\frac{2\lambda}{\mu}}E_1\left(\frac{2\lambda}{\mu}\right)-\frac{2\lambda}{\mu}\right\}\right].$$

▲ Bayesian estimators of the Rényi entropy under the LINEX loss function. In that case, $u(\mu,\lambda) = e^{-(H_\alpha)\,p}$, thus;

$$\hat{u}_{R.\mu} = -p\,e^{-p\,H_\alpha}\left[\frac{1}{1-\alpha}\left[\left(1-\frac{3\alpha}{2}\right)\frac{1}{\mu}-\frac{\alpha\lambda}{\mu^2}-\frac{\alpha\lambda}{\mu^2}\left(\frac{K'_{1-\frac{3\alpha}{2}}\left(\frac{\alpha\lambda}{\mu}\right)}{K_{1-\frac{3\alpha}{2}}\left(\frac{\alpha\lambda}{\mu}\right)}\right)\right]\right],$$

$$\hat{u}_{R.\mu\mu} = \frac{p^2\,e^{-p\,H_\alpha}}{[1-\alpha]^2}\left[\left(1-\frac{3\alpha}{2}\right)\frac{1}{\mu}-\frac{\alpha\lambda}{\mu^2}-\frac{\alpha\lambda}{\mu^2}\left(\frac{K'_{1-\frac{3\alpha}{2}}\left(\frac{\alpha\lambda}{\mu}\right)}{K_{1-\frac{3\alpha}{2}}\left(\frac{\alpha\lambda}{\mu}\right)}\right)\right]^2 - \frac{p\,e^{-p\,H_\alpha}}{1-\alpha}\left[\left(\frac{3\alpha}{2}-1\right)\frac{1}{\mu^2}+\frac{\alpha\lambda}{\mu^3}\right.$$

$$\left. -\frac{2\alpha\lambda}{\mu^3}\left(\frac{K'_{1-\frac{3\alpha}{2}}\left(\frac{\alpha\lambda}{\mu}\right)}{K_{1-\frac{3\alpha}{2}}\left(\frac{\alpha\lambda}{\mu}\right)}\right)+\frac{\alpha^2\lambda^2}{\mu^4}\left[\frac{K''_{1-\frac{3\alpha}{2}}\left(\frac{\alpha\lambda}{\mu}\right)}{K_{1-\frac{3\alpha}{2}}\left(\frac{\alpha\lambda}{\mu}\right)}-\left(\frac{K'_{1-\frac{3\alpha}{2}}\left(\frac{\alpha\lambda}{\mu}\right)}{K_{1-\frac{3\alpha}{2}}\left(\frac{\alpha\lambda}{\mu}\right)}\right)^2\right]\right],$$

$$\hat{u}_{R.\lambda} = -p\,e^{-p\,H_\alpha}\left[\frac{1}{1-\alpha}\left[\frac{\alpha}{2\lambda}+\frac{\alpha}{\mu}-\frac{\alpha}{\mu}\left(\frac{K'_{1-\frac{3\alpha}{2}}\left(\frac{\alpha\lambda}{\mu}\right)}{K_{1-\frac{3\alpha}{2}}\left(\frac{\alpha\lambda}{\mu}\right)}\right)\right]\right],$$

$$\hat{u}_{R.\lambda\lambda} = \frac{p^2\,e^{-p\,H_\alpha}}{[1-\alpha]^2}\left[\frac{\alpha}{2\lambda}+\frac{\alpha}{\mu}-\frac{\alpha}{\mu}\left(\frac{K'_{1-\frac{3\alpha}{2}}\left(\frac{\alpha\lambda}{\mu}\right)}{K_{1-\frac{3\alpha}{2}}\left(\frac{\alpha\lambda}{\mu}\right)}\right)\right]^2$$

$$-\frac{p\,e^{-p\,H_\alpha}}{1-\alpha}\left[-\frac{\alpha}{2\lambda^2}-\left(\frac{\alpha}{\mu}\right)^2\left[\frac{K''_{1-\frac{3\alpha}{2}}\left(\frac{\alpha\lambda}{\mu}\right)}{K_{1-\frac{3\alpha}{2}}\left(\frac{\alpha\lambda}{\mu}\right)}-\left(\frac{K'_{1-\frac{3\alpha}{2}}\left(\frac{\alpha\lambda}{\mu}\right)}{K_{1-\frac{3\alpha}{2}}\left(\frac{\alpha\lambda}{\mu}\right)}\right)^2\right]\right],$$

$$\hat{u}_{R.\mu\lambda} = \hat{u}_{R.\lambda\mu} = \frac{p^2\,e^{-p\,H_\alpha}}{(1-\alpha)^2}\left[\left(1-\frac{3\alpha}{2}\right)\frac{1}{\mu}-\frac{\alpha\lambda}{\mu^2}-\frac{\alpha\lambda}{\mu^2}\left(\frac{K'_{1-\frac{3\alpha}{2}}\left(\frac{\alpha\lambda}{\mu}\right)}{K_{1-\frac{3\alpha}{2}}\left(\frac{\alpha\lambda}{\mu}\right)}\right)\right]\left[\frac{\alpha}{2\lambda}+\frac{\alpha}{\mu}-\frac{\alpha}{\mu}\frac{K'_{1-\frac{3\alpha}{2}}\left(\frac{\alpha\lambda}{\mu}\right)}{K_{1-\frac{3\alpha}{2}}\left(\frac{\alpha\lambda}{\mu}\right)}\right]$$

$$-\frac{p\,e^{-p\,H_\alpha}}{1-\alpha}\left[-\frac{\alpha}{\mu^2}-\frac{\alpha}{\mu^2}\left(\frac{K'_{1-\frac{3\alpha}{2}}\left(\frac{\alpha\lambda}{\mu}\right)}{K_{1-\frac{3\alpha}{2}}\left(\frac{\alpha\lambda}{\mu}\right)}\right)-\frac{\alpha^2\lambda}{\mu^3}\left[\frac{K''_{1-\frac{3\alpha}{2}}\left(\frac{\alpha\lambda}{\mu}\right)}{K_{1-\frac{3\alpha}{2}}\left(\frac{\alpha\lambda}{\mu}\right)}-\left(\frac{K'_{1-\frac{3\alpha}{2}}\left(\frac{\alpha\lambda}{\mu}\right)}{K_{1-\frac{3\alpha}{2}}\left(\frac{\alpha\lambda}{\mu}\right)}\right)^2\right]\right].$$

The derivatives of the maximum likelihood estimators equations. The log-likelihood function is expressed as:

$$\begin{aligned}\ell(\mu,\lambda|x) &\propto \frac{m}{2}\ln\left(\frac{\lambda}{2\pi}\right)+\sum_{i=1}^{m}\ln\left[\frac{1}{x_i}\right]-\sum_{i=1}^{m}\frac{\lambda(x_i-\mu)^2}{2\mu^2 x_i}+\sum_{i=1}^{m}R_i\\ &\times \ln\left(1-\left(\Phi\left[\sqrt{\frac{\lambda}{x_i}}\left(\frac{x_i}{\mu}-1\right)\right]+e^{\frac{2\lambda}{\mu}}\Phi\left[-\sqrt{\frac{\lambda}{x_i}}\left(\frac{x_i}{\mu}+1\right)\right]\right)\right)\end{aligned}$$

For μ, which is given by:

$$\ell_\mu = -\sum_{i=1}^{m}\frac{\lambda(\mu-x_i)}{\mu^3}+\sum_{i=1}^{m}R_i\frac{\Phi\sqrt{\lambda}\left[\frac{\sqrt{x_i}}{\mu^2}\left(e^{\frac{2\lambda}{\mu}}-1\right)+e^{\frac{2\lambda}{\mu}}\left(\frac{2\lambda\sqrt{x_i}}{\mu^3}-\frac{2\lambda}{\mu^2\sqrt{x_i}}\right)\right]}{\left(1-\left(\Phi\left[\sqrt{\frac{\lambda}{x_i}}\left(\frac{x_i}{\mu}-1\right)\right]+e^{\frac{2\lambda}{\mu}}\Phi\left[-\sqrt{\frac{\lambda}{x_i}}\left(\frac{x_i}{\mu}+1\right)\right]\right)\right)} \quad (1)$$

$B_i = \frac{\lambda(x_i-\mu)^2}{2\mu^2 x_i}$ | $B_{i,\mu\mu\lambda} = 3\mu^{-4}x_i - 2\mu^{-3}$

$B_{i,\mu} = \frac{\lambda(\mu-x_i)}{\mu^3}$ | $B_{i,\mu\lambda} = -\mu^{-3}x_i + \mu^{-2}$

$B_{i,\mu\mu} = 3\mu^{-4}\lambda x_i - 2\mu^{-3}\lambda$ | $B_{i,\mu\lambda\lambda} = 0$

$B_{i,\mu\mu\mu} = -12\mu^{-5}\lambda x_i + 6\mu^{-4}\lambda$ | $B_{i,\mu\lambda\mu} = 3\mu^{-4}x_i - 2\mu^{-3}$

$$A_i = 1-\left(\Phi\left[\sqrt{\frac{\lambda}{x_i}}\left(\frac{x_i}{\mu}-1\right)\right]+e^{\frac{2\lambda}{\mu}}\Phi\left[-\sqrt{\frac{\lambda}{x_i}}\left(\frac{x_i}{\mu}+1\right)\right]\right)$$

$$
\begin{aligned}
A_{i,\mu} &= \Phi\sqrt{\lambda}\left[\frac{\sqrt{x_i}}{\mu^2}\left(e^{\frac{2\lambda}{\mu}}-1\right)+e^{\frac{2\lambda}{\mu}}\left(\frac{2\lambda\sqrt{x_i}}{\mu^3}-\frac{2\lambda}{\mu^2\sqrt{x_i}}\right)\right] \\
&= \frac{\Phi\sqrt{\lambda}\,\sqrt{x_i}\,e^{\frac{2\lambda}{\mu}}}{\mu^2}+\frac{2\Phi\lambda^{3/2}\,\sqrt{x_i}\,e^{\frac{2\lambda}{\mu}}}{\mu^3}-\frac{2\Phi\lambda^{3/2}\,e^{\frac{2\lambda}{\mu}}}{\mu^2\sqrt{x_i}}-\frac{\Phi\sqrt{\lambda}\,\sqrt{x_i}}{\mu^2}
\end{aligned}
$$

$$
\begin{aligned}
A_{i,\mu\mu} &= -\frac{4\Phi\,\lambda^{5/2}\,\sqrt{x_i}\,e^{\frac{2\lambda}{\mu}}}{\mu^5}-\frac{8\Phi\,\lambda^{3/2}\,\sqrt{x_i}\,e^{\frac{2\lambda}{\mu}}}{\mu^4}-\frac{2\Phi\,\sqrt{\lambda}\,\sqrt{x_i}\,e^{\frac{2\lambda}{\mu}}}{\mu^3}+\frac{4\Phi\,\lambda^{5/2}\,e^{\frac{2\lambda}{\mu}}}{\sqrt{x_i}\,\mu^4}+\frac{4\Phi\,\lambda^{3/2}\,e^{\frac{2\lambda}{\mu}}}{\sqrt{x_i}\,\mu^3} \\
&\quad+\frac{2\Phi\,\sqrt{\lambda}\,\sqrt{x_i}}{\mu^3}
\end{aligned}
$$

$$
\begin{aligned}
A_{i,\mu\mu\mu} &= \frac{4\Phi\,\lambda^{3/2}\,\sqrt{x_i}\,e^{\frac{2\lambda}{\mu}}}{\mu^5}+\frac{6\Phi\,\sqrt{\lambda}\,\sqrt{x_i}\,e^{\frac{2\lambda}{\mu}}}{\mu^4}-\frac{6\Phi\,\sqrt{\lambda}\,\sqrt{x_i}}{\mu^4}-\frac{12\Phi\,\lambda^{3/2}\,e^{\frac{2\lambda}{\mu}}}{\sqrt{x_i}\,\mu^4}+\frac{32\Phi\,\lambda^{3/2}\,\sqrt{x_i}\,e^{\frac{2\lambda}{\mu}}}{\mu^5} \\
&\quad-\frac{24\Phi\,\lambda^{5/2}\,e^{\frac{2\lambda}{\mu}}}{\sqrt{x_i}\,\mu^5}+\frac{36\Phi\,\lambda^{5/2}\,\sqrt{x_i}\,e^{\frac{2\lambda}{\mu}}}{\mu^6}-\frac{8\Phi\,\lambda^{7/2}\,e^{\frac{2\lambda}{\mu}}}{\sqrt{x_i}\,\mu^6}+\frac{8\Phi\,\lambda^{7/2}\,\sqrt{x_i}\,e^{\frac{2\lambda}{\mu}}}{\mu^7}
\end{aligned}
$$

$$
\begin{aligned}
A_{i,\mu\mu\lambda} &= -\frac{10\Phi\,\sqrt{x_i}\,\lambda^{3/2}\,e^{\frac{2\lambda}{\mu}}}{\mu^5}-\frac{8\Phi\,\sqrt{x_i}\,\lambda^{5/2}\,e^{\frac{2\lambda}{\mu}}}{\mu^6}-\frac{12\Phi\,\sqrt{x_i}\,\lambda^{1/2}\,e^{\frac{2\lambda}{\mu}}}{\mu^4}-\frac{16\Phi\,\sqrt{x_i}\,\lambda^{3/2}\,e^{\frac{2\lambda}{\mu}}}{\mu^5} \\
&\quad-\frac{\Phi\,\sqrt{x_i}\,\lambda^{-1/2}\,e^{\frac{2\lambda}{\mu}}}{\mu^3}-\frac{4\Phi\,\sqrt{x_i}\,\lambda^{1/2}\,e^{\frac{2\lambda}{\mu}}}{\mu^4}+\frac{10\Phi\,\lambda^{3/2}\,e^{\frac{2\lambda}{\mu}}}{\sqrt{x_i}\,\mu^4}+\frac{8\Phi\,\lambda^{5/2}\,e^{\frac{2\lambda}{\mu}}}{\sqrt{x_i}\,\mu^5} \\
&\quad+\frac{6\Phi\,\lambda^{1/2}\,e^{\frac{2\lambda}{\mu}}}{\sqrt{x_i}\,\mu^3}+\frac{8\Phi\,\lambda^{3/2}\,e^{\frac{2\lambda}{\mu}}}{\sqrt{x_i}\,\mu^4}+\frac{\Phi\,\sqrt{x_i}\,\lambda^{-1/2}}{\mu^3}
\end{aligned}
$$

$$
\begin{aligned}
A_{i,\mu\lambda} &= \frac{\Phi\,\sqrt{x_i}\,e^{\frac{2\lambda}{\mu}}}{2\,\mu^2\sqrt{\lambda}}+\frac{5\Phi\,\sqrt{x_i}\,\sqrt{\lambda}\,e^{\frac{2\lambda}{\mu}}}{\mu^3}+\frac{4\Phi\,\lambda^{3/2}\,\sqrt{x_i}\,e^{\frac{2\lambda}{\mu}}}{\mu^4}-\frac{3\Phi\,\sqrt{\lambda}\,e^{\frac{2\lambda}{\mu}}}{\mu^2\sqrt{x_i}}-\frac{4\Phi\,\lambda^{3/2}\,e^{\frac{2\lambda}{\mu}}}{\mu^3\sqrt{x_i}} \\
&\quad-\frac{\Phi\,\sqrt{x_i}}{2\,\mu^2\sqrt{\lambda}}
\end{aligned}
$$

$$
\begin{aligned}
A_{i,\mu\lambda\lambda} &= \frac{16\Phi\,\lambda^{5/2}e^{\frac{2\lambda}{\mu}}}{\mu^6\sqrt{x_i}}-\frac{16\Phi\,\lambda^{5/2}\sqrt{x_i}\,e^{\frac{2\lambda}{\mu}}}{\mu^7}+\frac{56\Phi\,\lambda^{3/2}e^{\frac{2\lambda}{\mu}}}{\mu^5\sqrt{x_i}}-\frac{72\Phi\,\lambda^{3/2}\sqrt{x_i}\,e^{\frac{2\lambda}{\mu}}}{\mu^6} \\
&\quad+\frac{39\Phi\,\sqrt{\lambda}\,e^{\frac{2\lambda}{\mu}}}{\mu^4\sqrt{x_i}}-\frac{71\Phi\,\sqrt{\lambda}\,\sqrt{x_i}\,e^{\frac{2\lambda}{\mu}}}{\mu^5}+\frac{3\Phi\,e^{\frac{2\lambda}{\mu}}}{\sqrt{\lambda}\,\mu^3\sqrt{x_i}}-\frac{10\Phi\,\sqrt{x_i}\,e^{\frac{2\lambda}{\mu}}}{\sqrt{\lambda}\,\mu^4} \\
&\quad+\frac{\Phi\,\sqrt{x_i}\,e^{\frac{2\lambda}{\mu}}}{2\lambda^{3/2}\,\mu^3}-\frac{\Phi\,\sqrt{x_i}}{2\lambda^{3/2}\,\mu^3}
\end{aligned}
$$

$$
\begin{aligned}
A_{i,\mu\lambda\mu} &= \frac{8\Phi\,\lambda^{5/2}\,e^{\frac{2\lambda}{\mu}}}{\mu^5\sqrt{x_i}}-\frac{8\Phi\,\lambda^{5/2}\,\sqrt{x_i}\,e^{\frac{2\lambda}{\mu}}}{\mu^6}+\frac{18\Phi\,\lambda^{3/2}\,e^{\frac{2\lambda}{\mu}}}{\mu^4\sqrt{x_i}}-\frac{26\Phi\,\lambda^{3/2}\,\sqrt{x_i}\,e^{\frac{2\lambda}{\mu}}}{\mu^5} \\
&\quad+\frac{6\Phi\,\sqrt{\lambda}\,e^{\frac{2\lambda}{\mu}}}{\mu^3\sqrt{x_i}}-\frac{16\Phi\,\sqrt{\lambda}\,\sqrt{x_i}\,e^{\frac{2\lambda}{\mu}}}{\mu^4}-\frac{\Phi\,\sqrt{x_i}\,e^{\frac{2\lambda}{\mu}}}{\sqrt{\lambda}\,\mu^3}+\frac{\Phi\,\sqrt{x_i}}{\sqrt{\lambda}\,\mu^3}
\end{aligned}
$$

For λ, which is given by:

$$
\begin{aligned}
\ell_\lambda &= \frac{m}{2\lambda}-\sum_{i=1}^{m}\frac{(x_i-\mu)^2}{2\mu^2 x_i}+\sum_{i=1}^{m}R_i \\
&\times\left(\frac{\Phi\left(\frac{1}{2\sqrt{\lambda}}\left(\frac{\sqrt{x_i}}{\mu}-\frac{1}{\sqrt{x_i}}\right)+e^{\frac{2\lambda}{\mu}}\left(\frac{\sqrt{x_i}}{\mu}\left(\frac{-2\sqrt{\lambda}}{\mu}-\frac{1}{2\sqrt{\lambda}}\right)+\frac{1}{\sqrt{x_i}}\left(\frac{2\sqrt{\lambda}}{\mu}+\frac{1}{2\sqrt{\lambda}}\right)\right)\right)}{\left(1-\left(\Phi\left[\sqrt{\frac{\lambda}{x_i}}\left(\frac{x_i}{\mu}-1\right)\right]+e^{\frac{2\lambda}{\mu}}\Phi\left[-\sqrt{\frac{\lambda}{x_i}}\left(\frac{x_i}{\mu}+1\right)\right]\right)\right)}\right)
\end{aligned} \tag{2}
$$

$$C = \frac{m}{2}\ln\left(\frac{\lambda}{2\pi}\right)$$

$$C_\lambda = \frac{m}{2\lambda}$$

$$C_{\lambda\lambda} = -\frac{m}{2\lambda^2}$$

$$C_{\lambda\lambda\lambda} = \frac{m}{\lambda^3}$$

$$C_{\lambda\mu} = C_{\lambda\lambda\mu} = C_{\lambda\mu\mu} = C_{\lambda\mu\lambda} = 0$$

$$B_{i,\lambda} = \frac{(x_i-\mu)^2}{2\mu^2 x_i}$$

$$B_{i,\lambda\lambda} = B_{i,\lambda\lambda\lambda} = B_{i,\lambda\lambda\mu} = B_{i,\lambda\mu\lambda} = 0$$

$$B_{i,\lambda\mu} = -x_i\mu^{-3} + \mu^{-2}$$

$$B_{i,\lambda\mu\mu} = 3x_i\mu^{-4} - 2\mu^{-3}$$

$$\begin{aligned}
A_{i,\lambda} &= \Phi\left(\frac{1}{2\sqrt{\lambda}}\left[\frac{\sqrt{x_i}}{\mu} - \frac{1}{\sqrt{x_i}}\right] + e^{\frac{2\lambda}{\mu}}\left[\frac{\sqrt{x_i}}{\mu}\left(\frac{-2\sqrt{\lambda}}{\mu} - \frac{1}{2\sqrt{\lambda}}\right) + \frac{1}{\sqrt{x_i}}\left(\frac{2\sqrt{\lambda}}{\mu} + \frac{1}{2\sqrt{\lambda}}\right)\right]\right) \\
&= \frac{\Phi\sqrt{x_i}}{2\mu\sqrt{\lambda}} - \frac{\Phi}{2\sqrt{x_i}\sqrt{\lambda}} - \frac{2\Phi\sqrt{x_i}\sqrt{\lambda}\,e^{\frac{2\lambda}{\mu}}}{\mu^2} - \frac{\Phi\sqrt{x_i}\,e^{\frac{2\lambda}{\mu}}}{2\mu\sqrt{\lambda}} + \frac{2\Phi\sqrt{\lambda}\,e^{\frac{2\lambda}{\mu}}}{\mu\sqrt{x_i}} + \frac{\Phi\,e^{\frac{2\lambda}{\mu}}}{2\sqrt{\lambda}\sqrt{x_i}}
\end{aligned}$$

$$\begin{aligned}
A_{i,\lambda\lambda} &= -\frac{1}{4}\lambda^{-\frac{3}{2}}\Phi\,x_i^{\frac{1}{2}}\mu^{-1} + \frac{1}{4}\lambda^{-\frac{3}{2}}\Phi\,x_i^{-\frac{1}{2}} - \lambda^{-\frac{1}{2}}\Phi\,x_i^{\frac{1}{2}}\mu^{-2}e^{\frac{2\lambda}{\mu}} - 4\lambda^{\frac{1}{2}}\Phi\,x_i^{\frac{1}{2}}\mu^{-3}e^{\frac{2\lambda}{\mu}} \\
&- \frac{1}{4}\lambda^{-\frac{3}{2}}\Phi\,x_i\mu^{-1}e^{\frac{2\lambda}{\mu}} - \lambda^{-\frac{1}{2}}\Phi\,x_i\mu^{-2}e^{\frac{2\lambda}{\mu}} + 4\lambda^{\frac{1}{2}}\Phi\,x_i\mu^{-2}e^{\frac{2\lambda}{\mu}} + 2\lambda^{-\frac{1}{2}}\Phi\,x_i^{-\frac{1}{2}}\mu^{-1}e^{\frac{2\lambda}{\mu}} \\
&- \frac{1}{4}\lambda^{-\frac{3}{2}}\Phi\,x_i^{-\frac{1}{2}}e^{\frac{2\lambda}{\mu}}
\end{aligned}$$

$$\begin{aligned}
A_{i,\lambda\lambda\lambda} &= -\frac{3}{8}\lambda^{-\frac{5}{2}}\Phi\,x_i^{-\frac{1}{2}} - \Phi\,x_i^{\frac{1}{2}}\mu^{-2}\left(-\frac{1}{2}\lambda^{-\frac{3}{2}}e^{\frac{2\lambda}{\mu}} + \frac{2\lambda^{-\frac{1}{2}}}{\mu}e^{\frac{2\lambda}{\mu}}\right) - 4\Phi\,x_i^{\frac{1}{2}}\mu^{-3}\left(-\frac{1}{2}\lambda^{-\frac{1}{2}}e^{\frac{2\lambda}{\mu}} + \frac{2\lambda^{\frac{1}{2}}}{\mu}e^{\frac{2\lambda}{\mu}}\right) \\
&- \frac{1}{4}\Phi\,x_i^{\frac{1}{2}}\mu^{-1}\left(-\frac{3}{2}\lambda^{-\frac{5}{2}}e^{\frac{2\lambda}{\mu}} + \frac{2\lambda^{-\frac{3}{2}}}{\mu}e^{\frac{2\lambda}{\mu}}\right) - \Phi\,x_i\mu^{-2}\left(-\frac{1}{2}\lambda^{-\frac{3}{2}}e^{\frac{2\lambda}{\mu}} + \frac{2\lambda^{-\frac{1}{2}}}{\mu}e^{\frac{2\lambda}{\mu}}\right) \\
&+ 4\Phi\,x_i\mu^{-2}\left(\frac{1}{2}\lambda^{-\frac{1}{2}}e^{\frac{2\lambda}{\mu}} + \frac{2\lambda^{\frac{1}{2}}}{\mu}e^{\frac{2\lambda}{\mu}}\right) + 2\Phi\,x_i^{-\frac{1}{2}}\mu^{-1}\left(-\frac{1}{2}\lambda^{-\frac{3}{2}}e^{\frac{2\lambda}{\mu}} + \frac{2\lambda^{-\frac{3}{2}}}{\mu}e^{\frac{2\lambda}{\mu}}\right) \\
&- \frac{1}{4}\Phi\,x_i^{-\frac{1}{2}}\left(-\frac{3}{2}\lambda^{-\frac{5}{2}}e^{\frac{2\lambda}{\mu}} + \frac{2\lambda^{-\frac{3}{2}}}{\mu}e^{\frac{2\lambda}{\mu}}\right)
\end{aligned}$$

$$\begin{aligned}
A_{i,\lambda\lambda\mu} &= \frac{1}{4}\lambda^{-\frac{3}{2}}\Phi\,x_i^{\frac{1}{2}}\mu^{-2} - \lambda^{-\frac{1}{2}}\Phi\,x_i^{\frac{1}{2}}\left(-2\mu^{-3}e^{\frac{2\lambda}{\mu}} - 2\lambda\mu^{-4}e^{\frac{2\lambda}{\mu}}\right) \\
&- 4\lambda^{\frac{1}{2}}\Phi\,x_i^{\frac{1}{2}}\left(-3\mu^{-4}e^{\frac{2\lambda}{\mu}} - 2\lambda\mu^{-5}e^{\frac{2\lambda}{\mu}}\right) - \frac{1}{4}\lambda^{-\frac{3}{2}}\Phi\,x_i\left(\mu^{-2}e^{\frac{2\lambda}{\mu}} - 2\lambda\mu^{-3}\right) \\
&- \lambda^{-\frac{1}{2}}\Phi\,x_i\left(-2\mu^{-3}e^{\frac{2\lambda}{\mu}} - 2\lambda\mu^{-4}e^{\frac{2\lambda}{\mu}}\right) + 4\lambda^{\frac{1}{2}}\Phi\,x_i\left(-2\mu^{-3}e^{\frac{2\lambda}{\mu}} - 2\lambda\mu^{-4}e^{\frac{2\lambda}{\mu}}\right) \\
&+ 2\lambda^{-\frac{1}{2}}\Phi\,x_i\left(\mu^{-2}e^{\frac{2\lambda}{\mu}} - 2\lambda\mu^{-3}e^{\frac{2\lambda}{\mu}}\right) + \frac{1}{2}\lambda^{-\frac{3}{2}}\Phi\,x^{-\frac{1}{2}}\mu^{-2}e^{\frac{2\lambda}{\mu}}
\end{aligned}$$

$$\begin{aligned}
A_{i,\lambda\mu} &= \frac{-1}{2}\lambda^{-\frac{1}{2}}\Phi\,x_i^{\frac{1}{2}}\mu^{-2} + 4\Phi\,x_i^{\frac{1}{2}}\lambda^{\frac{3}{2}}\mu^{-4}e^{\frac{2\lambda}{\mu}} + 4\Phi\,x_i^{\frac{1}{2}}\lambda^{\frac{1}{2}}\mu^{-3}e^{\frac{2\lambda}{\mu}} + \Phi\,x_i\lambda^{\frac{1}{2}}\mu^{-3}e^{\frac{2\lambda}{\mu}} \\
&+ \frac{1}{2}\Phi\,x_i\lambda^{-\frac{1}{2}}\mu^{-2}e^{\frac{2\lambda}{\mu}} - 4\Phi\,x_i^{-\frac{1}{2}}\lambda^{\frac{3}{2}}\mu^{-3}e^{\frac{2\lambda}{\mu}} - 3\Phi\,x_i^{-\frac{1}{2}}\lambda^{\frac{1}{2}}\mu^{-2}e^{\frac{2\lambda}{\mu}}
\end{aligned}$$

$$\begin{aligned}
A_{i,\lambda\mu\mu} &= \mu^{-3}\lambda^{-\frac{1}{2}}\Phi\,x_i^{\frac{1}{2}} + 4\Phi\,x_i^{\frac{1}{2}}\lambda^{\frac{3}{2}}\left(-4\mu^{-5}e^{\frac{2\lambda}{\mu}} - 2\lambda\mu^{-6}e^{\frac{2\lambda}{\mu}}\right) + 4\Phi\,x_i^{\frac{1}{2}}\lambda^{\frac{1}{2}}\left(-3\mu^{-4}e^{\frac{2\lambda}{\mu}} - 2\lambda\mu^{-5}e^{\frac{2\lambda}{\mu}}\right) \\
&+ \Phi\,x_i\lambda^{\frac{1}{2}}\left(-3\mu^{-4}e^{\frac{2\lambda}{\mu}} - 2\lambda\mu^{-5}e^{-\frac{2\lambda}{\mu}}\right) + \frac{1}{2}\Phi\,x_i\lambda^{-\frac{1}{2}}\left(-2\mu^{-3}e^{\frac{2\lambda}{\mu}} - 2\lambda\mu^{-4}e^{\frac{2\lambda}{\mu}}\right) \\
&- 4\Phi\,x_i^{-\frac{1}{2}}\lambda^{\frac{3}{2}}\left(-3\mu^{-4}e^{\frac{2\lambda}{\mu}} - 2\lambda\mu^{-5}e^{\frac{2\lambda}{\mu}}\right) - 3\Phi\,x_i^{-\frac{1}{2}}\lambda^{\frac{1}{2}}\left(-2\mu^{-3}e^{\frac{2\lambda}{\mu}} - 2\lambda\mu^{-4}e^{\frac{2\lambda}{\mu}}\right)
\end{aligned}$$

$$A_{i,\lambda\mu\lambda} = \frac{1}{4}\lambda^{-\frac{3}{2}}\Phi\,x_i^{\frac{1}{2}}\mu^{-2} + 4\Phi\,x_i^{\frac{1}{2}}\mu^{-4}\left(\frac{3}{2}\lambda^{\frac{1}{2}}e^{\frac{2\lambda}{\mu}} + \frac{2\lambda^{\frac{3}{2}}}{\mu}e^{\frac{2\lambda}{\mu}}\right)$$

$$
\begin{aligned}
&+ \quad 4\Phi\, x_i^{\frac{1}{2}}\mu^{-3}\left(\frac{1}{2}\lambda^{-\frac{1}{2}}e^{\frac{2\lambda}{\mu}} + \frac{2\lambda^{\frac{1}{2}}}{\mu}e^{\frac{2\lambda}{\mu}}\right) + \Phi\, x_i\mu^{-3}\left(\frac{1}{2}\lambda^{-\frac{1}{2}}e^{\frac{2\lambda}{\mu}} + \frac{2\lambda^{\frac{1}{2}}}{\mu}e^{\frac{2\lambda}{\mu}}\right)\\
&+ \quad \frac{1}{2}\Phi\, x_i\mu^{-2}\left(\frac{-1}{2}\lambda^{-\frac{3}{2}}e^{\frac{2\lambda}{\mu}} + \frac{2\lambda^{-\frac{1}{2}}}{\mu}e^{\frac{2\lambda}{\mu}}\right) - 4\Phi\, x_i^{-\frac{1}{2}}\mu^{-3}\left(\frac{3}{2}\lambda^{\frac{1}{2}}e^{\frac{2\lambda}{\mu}} + \frac{2\lambda^{\frac{3}{2}}}{\mu}e^{\frac{2\lambda}{\mu}}\right)\\
&- \quad 3\Phi\, x_i^{-\frac{1}{2}}\mu^{-2}\left(\frac{1}{2}\lambda^{-\frac{1}{2}}e^{\frac{2\lambda}{\mu}} + \frac{2\lambda^{\frac{1}{2}}}{\mu}e^{\frac{2\lambda}{\mu}}\right)
\end{aligned}
$$

Based on the above,

$$
\begin{aligned}
\ell_{\mu} &= \textstyle\sum_{i=1}^m B_{i,\mu} + \sum_{i=1}^m R_i \frac{A_{i,\mu}}{A_i}\\
\ell_{\mu\mu} &= \textstyle\sum_{i=1}^m B_{i,\mu\mu} + \sum_{i=1}^m R_i \left(\frac{A_{i,\mu\mu}A_i - A_{i,\mu}^2}{A_i^2}\right)\\
\ell_{\mu\mu\mu} &= \textstyle\sum_{i=1}^m B_{i,\mu\mu\mu} + \sum_{i=1}^m R_i \left[\frac{A_{i\mu\mu\mu}A_i^2 - 3A_{i,\mu}A_{i,\mu\mu}A_i + 2A_{i\mu}^3}{A_i^3}\right]\\
\ell_{\mu\mu\lambda} &= \textstyle\sum_{i=1}^m B_{i,\mu\mu\lambda} + \sum_{i=1}^m R_i \left[\frac{A_{i,\mu\mu\lambda}A_i^2 - A_iA_{i,\mu\mu}A_{i,\lambda} - 2A_iA_{i,\mu}A_{i,\mu\lambda} + 2A_{i,\mu}^2A_{i,\lambda}}{A_i^3}\right]\\
\ell_{\mu\lambda} &= \textstyle\sum_{i=1}^m B_{i,\mu\lambda} + \sum_{i=1}^m R_i \left(\frac{A_{i,\mu\lambda}A_i - A_{i,\mu}A_{i,\lambda}}{A_i^2}\right)\\
\ell_{\mu\lambda\lambda} &= \textstyle\sum_{i=1}^m B_{i,\mu\lambda\lambda} + \sum_{i=1}^m R_i \left[\frac{A_{i,\mu\lambda\lambda}A_i^2 - 2A_iA_{i,\mu\lambda}A_{i,\lambda} - A_iA_{i,\mu}A_{i,\lambda\lambda} + 2A_{i,\mu}A_{i,\lambda}^2}{A_i^3}\right]\\
\ell_{\mu\lambda\mu} &= \textstyle\sum_{i=1}^m B_{i,\mu\lambda\mu} + \sum_{i=1}^m R_i \left[\frac{A_{i,\mu\lambda\mu}A_i^2 - 2A_iA_{i,\mu}A_{i,\mu\lambda} - A_iA_{i,\mu\mu}A_{i,\lambda} + 2A_{i,\mu}^2A_{i,\lambda}}{A_i^3}\right]
\end{aligned}
$$

and

$$
\begin{aligned}
\ell_{\lambda} &= C_{\lambda} - \textstyle\sum_{i=1}^m B_{i,\lambda} + \sum_{i=1}^m R_i \frac{A_{i,\lambda}}{A_i}\\
\ell_{\lambda\lambda} &= C_{\lambda\lambda} - \textstyle\sum_{i=1}^m B_{i,\lambda\lambda} + \sum_{i=1}^m R_i \left(\frac{A_{i,\lambda\lambda}A_i - A_{i,\lambda}^2}{A_i^2}\right)\\
\ell_{\lambda\lambda\lambda} &= C_{\lambda\lambda\lambda} - \textstyle\sum_{i=1}^m B_{i,\lambda\lambda\lambda} + \sum_{i=1}^m R_i \left[\frac{A_{i,\lambda\lambda\lambda}A_i^2 - 3A_{i,\lambda}A_{i,\lambda\lambda}A_i + 2A_{i,\lambda}^3}{A_i^3}\right]\\
\ell_{\lambda\lambda\mu} &= C_{\lambda\lambda\mu} - \textstyle\sum_{i=1}^m B_{i,\lambda\lambda\mu} + \sum_{i=1}^m R_i \left[\frac{A_{i,\lambda\lambda\mu}A_i^2 - A_{i,\lambda\lambda}A_{i,\mu}A_i - 2A_{i,\lambda}A_{i,\lambda\mu}A_i + 2A_{i,\mu}A_{i,\lambda}^2}{A_i^3}\right]\\
\ell_{\lambda\mu} &= C_{\lambda\mu} - \textstyle\sum_{i=1}^m B_{i,\lambda\mu} + \sum_{i=1}^m R_i \left(\frac{A_{i,\lambda\mu}A_i - A_{i,\lambda}A_{i,\mu}}{A_i^2}\right)\\
\ell_{\lambda\mu\mu} &= C_{\lambda\mu\mu} - \textstyle\sum_{i=1}^m B_{i,\lambda\mu\mu} + \sum_{i=1}^m R_i \left[\frac{A_{i,\lambda\mu\mu}A_i^2 - A_{i,\lambda}A_{i,\mu\mu}A_i - 2A_{i,\lambda\mu}A_{i,\mu}A_i + 2A_{i,\lambda}A_{i,\mu}^2}{A_i^3}\right]\\
\ell_{\lambda\mu\lambda} &= C_{\lambda\mu\lambda} - \textstyle\sum_{i=1}^m B_{i,\lambda\mu\lambda} + \sum_{i=1}^m R_i \left[\frac{A_{i,\lambda\lambda\mu}A_i^2 - A_{i,\lambda\lambda}A_{i,\mu}A_i - 2A_{i,\lambda\mu}A_{i,\lambda}A_i + 2A_{i,\lambda}^2A_{i,\mu}}{A_i^3}\right]
\end{aligned}
$$

Then

$$
\begin{aligned}
\hat{\ell}_{\mu\mu\mu} &= \sum_{i=1}^m B_{i,\mu\mu\mu} + \sum_{i=1}^m R_i \left[\frac{A_{i,\mu\mu\mu}A_i^2 - 3A_{i,\mu}A_{i,\mu\mu}A_i + 2A_{i,\mu}^3}{A_i^3}\right]\\
\hat{\ell}_{\mu\mu\lambda} &= \sum_{i=1}^m B_{i,\mu\mu\lambda} + \sum_{i=1}^m R_i \left[\frac{A_{i,\mu\mu\lambda}A_i^2 - A_iA_{i,\mu\mu}A_{i,\lambda} - 2A_iA_{i,\mu}A_{i,\mu\lambda} + 2A_{i,\mu}^2A_{i,\lambda}}{A_i^3}\right]\\
\hat{\ell}_{\mu\lambda\mu} &= \sum_{i=1}^m B_{i,\mu\lambda\mu} + \sum_{i=1}^m R_i \left[\frac{A_{i,\mu\lambda\mu}A_i^2 - 2A_iA_{i,\mu}A_{i,\mu\lambda} - A_iA_{i,\mu\mu}A_{i,\lambda} + 2A_{i,\mu}^2A_{i,\lambda}}{A_i^3}\right]\\
\hat{\ell}_{\mu\lambda\lambda} &= \sum_{i=1}^m R_i \left[\frac{A_{i,\mu\lambda\lambda}A_i^2 - 2A_iA_{i,\mu\lambda}A_{i,\lambda} - A_iA_{i,\mu}A_{i,\lambda\lambda} + 2A_{i,\mu}A_{i,\lambda}^2}{A_i^3}\right]\\
\hat{\ell}_{\lambda\lambda\lambda} &= C_{\lambda\lambda\lambda} + \sum_{i=1}^m R_i \left[\frac{A_{i,\lambda\lambda\lambda}A_i^2 - 3A_{i,\lambda}A_{i,\lambda\lambda}A_i + 2A_{i,\lambda}^3}{A_i^3}\right]\\
\hat{\ell}_{\lambda\lambda\mu} &= \sum_{i=1}^m R_i \left[\frac{A_{i,\lambda\lambda\mu}A_i^2 - A_{i,\lambda\lambda}A_{i,\mu}A_i - 2A_{i,\lambda\mu}A_{i,\lambda}A_i + 2A_{i,\lambda}^2A_{i,\mu}}{A_i^3}\right]\\
\hat{\ell}_{\lambda\mu\lambda} &= \sum_{i=1}^m R_i \left[\frac{A_{i,\lambda\lambda\mu}A_i^2 - A_{i,\lambda\lambda}A_{i,\mu}A_i - 2A_{i,\lambda\mu}A_{i,\lambda}A_i + 2A_{i,\lambda}^2A_{i,\mu}}{A_i^3}\right]\\
\hat{\ell}_{\lambda\mu\mu} &= -\sum_{i=1}^m B_{i,\lambda\mu\mu} + \sum_{i=1}^m R_i \left[\frac{A_{i,\lambda\mu\mu}A_i^2 - A_{i,\lambda}A_{i,\mu\mu}A_i - 2A_{i,\lambda\mu}A_{i,\mu}A_i + 2A_{i,\lambda}A_{i,\mu}^2}{A_i^3}\right]
\end{aligned}
$$
